# Prognostic factors for liver, blood and kidney adverse events from glucocorticoid sparing immune-suppressing drugs in immune-mediated inflammatory diseases: a prognostic systematic review

**DOI:** 10.1136/rmdopen-2023-003588

**Published:** 2024-01-10

**Authors:** Joanna Leaviss, Christopher Carroll, Munira Essat, Danielle van der Windt, Matthew J Grainge, Tim Card, Richard Riley, Abhishek Abhishek

**Affiliations:** 1 SCHARR, The University of Sheffield, Sheffield, Yorkshire, UK; 2 School of Medicine, Keele University, Keele, UK; 3 Academic Unit of Lifespan and Population Health, School of Medicine, University of Nottingham, Nottingham, UK; 4 Division of Epidemiology and Public Health, University of Nottingham, Nottingham, UK; 5 Institute of Applied Health Research, College of Medical and Dental Sciences, University of Birmingham, Birmingham, UK; 6 National Institute for Health and Care Research (NIHR) Birmingham Biomedical Research Centre, Birmingham, UK; 7 Academic Rheumatology, University of Nottingham, Nottingham, UK

**Keywords:** autoimmune diseases, anti-inflammatory agents, non-steroidal, immune system diseases

## Abstract

**Background:**

Immune-suppressing drugs can cause liver, kidney or blood toxicity. Prognostic factors for these adverse-events are poorly understood.

**Purpose:**

To ascertain prognostic factors associated with liver, blood or kidney adverse-events in people receiving immune-suppressing drugs.

**Data sources:**

MEDLINE, Web of Science, EMBASE and the Cochrane library (01 January 1995 to 05 January 2023), and supplementary sources.

**Data extraction and synthesis:**

Data were extracted by one reviewer using a modified CHARMS-PF checklist and validated by another. Two independent reviewers assessed risk of bias using Quality in Prognostic factor Studies tool and assessed the quality of evidence using a Grading of Recommendations Assessment, Development and Evaluation-informed framework.

**Results:**

Fifty-six studies from 58 papers were included. High-quality evidence of the following associations was identified: elevated liver enzymes (6 studies) and folate non-supplementation (3 studies) are prognostic factors for hepatotoxicity in those treated with methotrexate; that mercaptopurine (vs azathioprine) (3 studies) was a prognostic factor for hepatotoxicity in those treated with thiopurines; that mercaptopurine (vs azathioprine) (3 studies) and poor-metaboliser status (4 studies) were prognostic factors for cytopenia in those treated with thiopurines; and that baseline elevated liver enzymes (3 studies) are a prognostic factor for hepatotoxicity in those treated with anti-tumour necrosis factors. Moderate and low quality evidence for several other demographic, lifestyle, comorbidities, baseline bloods/serologic or treatment-related prognostic factors were also identified.

**Limitations:**

Studies published before 1995, those with less than 200 participants and not published in English were excluded. Heterogeneity between studies included different cut-offs for prognostic factors, use of different outcome definitions and different adjustment factors.

**Conclusions:**

Prognostic factors for target-organ damage were identified which may be further investigated for their potential role in targeted (risk-stratified) monitoring.

**PROSPERO registration number:**

CRD42020208049.

**WHAT IS ALREADY KNOWN ON THIS TOPIC**

- Steroid sparing disease modifying anti-rheumatic drugs (DMARDs) are extensively used for treating inflammatory conditions, and, while effective, they can cause hepatitis, cytopenia and acute kidney injury.
- Three monthly monitoring blood tests are recommended to detect these adverse events early, with more frequent monitoring in those at greater risk of toxicity. Prognostic factors that may require closer monitoring are poorly understood.

**WHAT THIS STUDY ADDS**

- This extensive systematic review ascertained prognostic factors for myelotoxicity, hepatotoxicity and nephrotoxicity due to many non-biological DMARDs and anti-tumour necrosis factor-alpha agents in a broad range of inflammatory conditions.

**HOW THIS STUDY MIGHT AFFECT RESEARCH, PRACTICE OR POLICY**

- Several prognostic factors for target organ damage were identified that may require more frequent monitoring when present.

## Introduction

Rheumatoid arthritis (RA), inflammatory bowel disease (IBD), psoriasis (PsO)+/−arthritis (PsA), ankylosing spondylitis (AS) and systemic lupus erythematosus (SLE) affect over 4% of adults and are usually treated with immune-suppressing drugs such as methotrexate (MTX), azathioprine (AZA) and anti-tumour necrosis factor (TNF)-alpha.^1–6^ Although effective, these medicines can cause drug-induced hepatitis, acute kidney injury and/or cytopenia. Fortnightly-to-monthly blood testing is recommended when newly starting these treatments and regular testing is recommended thereafter.^7–10^ The intended purpose of blood test monitoring is to facilitate the detection of an asymptomatic adverse event, allowing treatment to be stopped before any substantial damage occurs. Many guidelines recommend fixed blood testing intervals, for example, 3 monthly^7^ while others recommend more frequent testing in the presence of prognostic factors associated with an increased risk of adverse events.^8–10^ However, these prognostic factors are either not specified^8^ or mentioned anecdotally.^9 10^ This systematic review therefore aimed to determine which prognostic factors predict the likelihood of these specified adverse events, and thus to aid decisions on testing frequency.

The review question was: ‘Which patient and treatment factors predict liver, blood and kidney related adverse-events, and related dose adjustments or discontinuations, in patients exposed to named, non-biologic and/or biologic immune suppressing drugs for longer than 3 months?’.

## Methods

This systematic review of prognostic factor studies was conducted in accordance with PROGnosis RESearch Strategy framework (focusing on prognostic factor research)^11^ and the guidance by Cochrane prognosis methods group^12^ and is reported in accordance with the Preferred Reporting Items for Systematic review and Meta-Analysis guidelines.^13^ The inclusion and exclusion criteria using the PICOTS system (Population, Index prognostic factor, Comparator prognostic factors, Outcome, Timing, Setting) 12 are presented in supplementary material (online supplemental methods). A protocol for this review was registered with and is published in the CRD PROSPERO database. The only alteration from the published protocol was the application of the following limitations on included studies: 1995 onwards and greater than 200 participants in the entire study.

10.1136/rmdopen-2023-003588.supp1 Supplementary data

### Data sources and searches

A search strategy was developed in consultation with an information specialist and the project team. Thesaurus and free-text terms for the relevant populations were combined with terms for the interventions and validated study design filters for adverse events and prognostic studies, trials, observational cohort and case–control studies.^14^ The following bibliographical databases were interrogated up to 31 December 2020: MEDLINE, Web of Science, EMBASE and the Cochrane library, from 1 January 1995 to 31 December 2020, without any language restrictions (online supplemental methods). Bibliographies of included studies and relevant systematic reviews were reviewed manually to identify any additional relevant studies. We excluded studies published before 1995 as inflammatory conditions were mainly treated with corticosteroids and the outcomes of patients from that era may not be relevant to the 21st century. To ensure the review was as current as possible, an update search was conducted on 5 January 2023: the same bibliographical databases were interrogated with the same search strategies but restricted to 1 January 2020 onwards.

### Study selection

Three reviewers (ME, CC and JL) independently screened 10% of the sample of the titles and abstracts of citations retrieved by the original searches to compare results for accuracy and clarity of the application of the criteria. Each reviewer then screened 30% of the remaining titles and abstracts each to identify articles that satisfied the inclusion criteria and were considered for full-text screening. At the full-text screening stage, two of the three reviewers independently made a judgement on inclusion of each of the full papers (CC, ME and JL); any disagreements on inclusion were resolved by discussion and, where necessary, consultation with another reviewer (AA). For the update search, the same process was followed, but the screening was conducted by two reviewers (CC and JL), with disagreements resolved as above.

### Data extraction and quality assessment

The following data were extracted based on a modified version of the [Checklist for cricial appraisal and data extraction for systematic reviews of prediction modelling studies for prognostic factors] CHARMS-PF checklist^12^: location; population and sample size; outcomes to be predicted; start and end of follow-up period; index and comparator prognostic factors; missing data; analysis; and results (estimates and corresponding SEs/SD or CI). The effect sizes of interest (eg, HRs), cut points and adjustment factors were also extracted. HRs were prioritised over rate ratios and ORs. We did not transform from one reporting scale to another. Crude (unadjusted), and estimates additionally adjusted for other patient characteristics were extracted with the latter estimates prioritised for the evidence synthesis. All data were extracted by the lead reviewers (JL and CC) and validated by at least one other reviewer (AA, TC, DvdW and MG). Any disagreements were resolved by consensus or referral to AA. No attempt was made to contact the authors of included studies to enquire about missing or incomplete data. Where estimates and 95% CI could be calculated from raw data this was calculated by one reviewer using Stata, and where only p values and group sample sizes were available the Campbell collaboration effect size calculator was used.^15^

The Quality in Prognostic factor Studies (QUIPS) tool^16^ was used to appraise risk of bias. Judgements of high, low or unclear risk of bias for each domain were independently made by two reviewers (AA and JL). Any disagreements were resolved by consensus or referral to DvdW. Review findings were synthesised using an approach informed by the Grading of Recommendations Assessment, Development and Evaluation (GRADE) framework to assess the quality of the evidence (certainty in the evidence) for each prognostic factor–outcome combination.^17^ Evidence from randomised controlled trials (RCTs) were considered to be high quality as a starting point, and those from observational studies were considered to be low quality. The quality of evidence was then upgraded for large effect size (up one or two levels depending on the magnitude of the effect size), dose response (up one level) and downgraded one level each for high risk of bias, imprecision, inconsistency and for single study. The evidence for each outcome was assessed using this framework independently by JL and AA, with disagreements resolved through discussion. Any uncertainties were discussed with DvdW.

### Data synthesis

Given the range of conditions, treatments and time points being reviewed, the studies were heterogeneous. Study characteristics and outcome data were tabulated and presented in a narrative synthesis. Also, inadequate reporting of prognostic studies, the limitations of indirect estimation methods and the uncertainties occasioned by conversion of different estimates of effect (HRs, risk ratios and ORs) indicated that the pooling of the data in a quantitative meta-analysis was not appropriate.

### Small study bias

Visual assessment of potential small study bias with funnel plots was planned to be performed if effect estimates from more than 10 studies for a prognostic factor were identified for a drug and adverse event outcome pair. However, this was not found to be the case in any instance.

### Role of the funding source

The sponsors were not involved in the design or conduct of the study, nor in the analysis of the data or the decision to submit the manuscript.

### Patient and public involvement

Patients with personal lived experiences of inflammatory conditions were involved in prioritising the broad area of research as being of relevance to them.

Patients advised that the systematic review should include all common inflammatory conditions.

Patients and the public members have advised that the results be shared as infographics and brief video on a study website.

## Results

**Figure 1:**
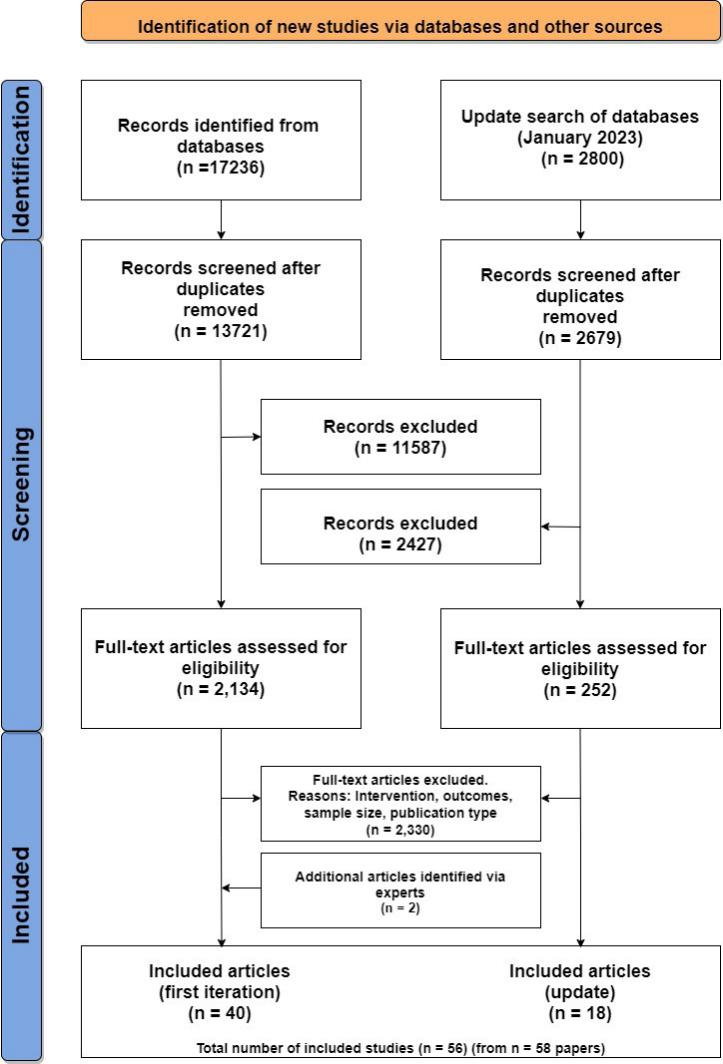
Preferred Reporting Items for Systematic review and Meta-Analysis (PRISMA) 2020 flow diagram (from Page *et al*).^13^ For more information, visit: http://www.prisma-statement.org/

After de-duplication 16 400 titles and abstracts were reviewed. From them 2386 full-text articles were assessed for eligibility of which 54 studies reported in 56 manuscripts were eligible. Two further studies were suggested by experts. Finally, 56 studies, reported in 58 manuscripts were included in this review (figure 1). Characteristics of study populations, interventions and outcomes are presented in online supplemental tables S1 and S2.

### Conventional DMARDs

They were evaluated in 43 studies (45 articles).^18–62^ The mean age of participants ranged from 25.7 years^56^ to 68.8 years.^19^ Most studies did not report drug naivety, however, nine and four studies, respectively, reported that all included patients were MTX^22 23 32 33 36–40^ and thiopurine naïve^47 48 51 52^, respectively. Thiopurines were evaluated in 18 studies, mainly in populations with IBD,^45–62^ however, one study also included patients with SLE or RA.^52^ Other conventional disease modifying anti-rheumatic drugs (DMARDs) were studied in RA,^18–23 25–30 32–34 36 38–40 42 44^ five in mixed populations of either RA, AS or Ps/PsA and SLE^24 31 35 37 41^ and one study in a PsO only population.^43^

For thiopurines there were nine retrospective cohort studies,^45 48–50 52 54 56–58^ six case–control studies,^47 55 59–62^ two prospective cohort studies,^46 53^ and one RCT.^51^ Other conventional DMARDs were studied in 13 retrospective cohort studies,^18 20 21 24 25 27 29–32 34 35 37 39 40 44^ 2 case–control studies,^23 36^ 7 prospective cohort studies,^19 33 38 41–43^ 1 RCT reanalysed as a cohort^22^ and 2 additional RCTs.^26 28^ Of those studies on MTX that reported concomitant medication, all participants were taking folic acid in six studies,^20 22 31 38 39 41 42^ while some but not all participants were taking folic acid in five further studies.^18 19 26 30 37^

### Anti-TNFs

Anti-TNFs were evaluated in 10 studies^63–72^ in patients with either RA, AS, PsO or PsA,^64 65 67 69^ AS only,^66^ 2 in PsA only^68 72^ and 3 in IBD.^63 70 71^ Of the 10 studies, 1 used an RCT design,^68^ 1 a prospective cohort design^72^ and the remaining were retrospective cohort studies.^63–67 69–71^ Two studies only evaluated infliximab,^68 70^ while the remaining studies evaluated two or more of the following anti-TNF drugs, entanercept,^64–67 69 72^ adalimumab,^63–67 69 71^ golimumab,^63–66^ infliximab^63 69^ and certolizumab.^63^ The mean age of participants ranged from 31 years^71^ to 57.5 years.^67^

### Combinations of drug classes

Three studies evaluated populations taking two or more different classes of drugs relevant to the review (online supplemental table S1).^73–75^ One study evaluated the AZA and MTX in populations with IBD and RA, respectively^73^; one study evaluated the use of mesalamine and infliximab and AZA in a population with IBD^74^; and the third study^75^ evaluated non-biological DMARDs or anti-TNFs in patients with PsA. Two studies were prospective cohorts with nested case–control studies,^74 75^ and one study had a retrospective cohort design.^73^ No details of dosing regime, drug naivety or concomitant medications were reported for any study. The mean age of participants ranged from 39 years^74^ to 51.9 years.^73^

#### Characteristics of outcomes

In some studies outcomes were grouped under broad headings of ‘myelotoxicity’ or ‘hepatotoxicity’, while in others outcomes were more specific, for example, ‘leukopenia’, or ‘elevation of transaminases’ (online supplemental table S1). Similar outcomes were grouped together in a clinically relevant outcome group and considered together in evidence synthesis. Differences were also found in how the outcome was recorded. Most studies reported any incidence of the outcome. In some studies, discontinuation of treatment or dose adjustment associated with the outcome was reported (online supplemental material).

#### Quality assessment

**Figure 2:**
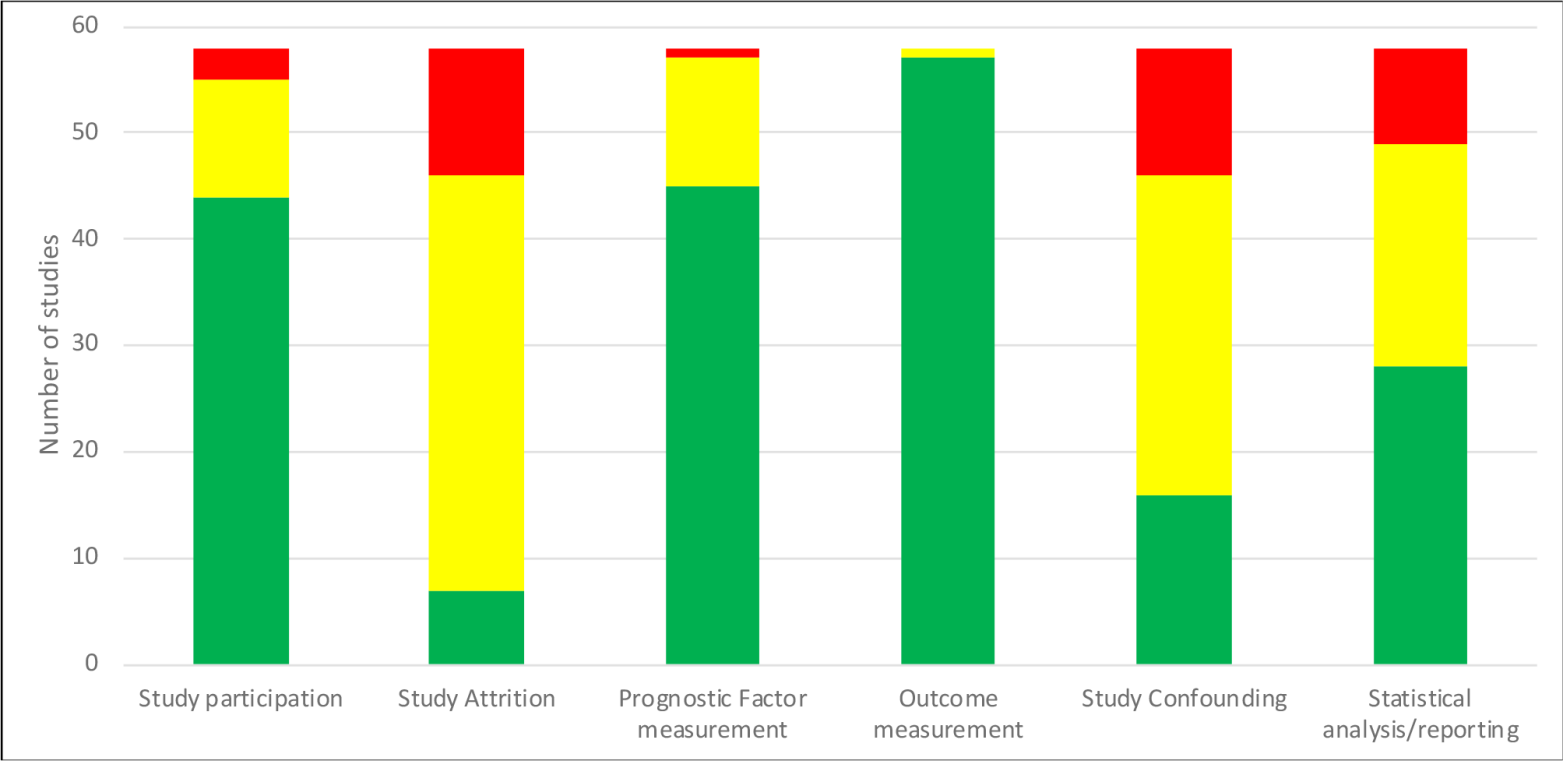
Summary of quality of included studies. Colours represent: Green - low risk of bias; yellow - moderate risk of bias; red - high risk of bias.

The results of the quality assessments of all 58 included articles (56 studies) using the QUIPS (Quality In Prognosis) tool are presented in figure 2 (details in online supplemental table S3) and a summary of findings for each domain is summarised below in a narrative synthesis.

### Study participation

Participants in most studies met the criteria for the specific inflammatory diseases of interest with baseline characteristics adequately described. However, where retrospective analysis of a cohort using data sets (eg, from one institution) were used, it was often unclear how many eligible participants were screened for inclusion. In these cases, it was often not possible to tell whether only participants who met the criteria for inclusion and had data for all relevant outcomes were included in the analyses, and if so, how many potentially eligible participants were excluded due to a lack of data. This presents a risk of selection bias across these studies.

### Study attrition

A lack of clear reporting of attrition and missing data in the retrospective cohort studies meant that it was not possible to judge whether there was any, and if so how much data was missing for each variable of interest. This level of detail was rarely reported, leading to the risk of bias from potentially missing data. Furthermore, in these cases, it was not possible to judge whether patients whose data was missing differed in characteristics compared with those without missing data.

### Prognostic factor measurement

Definitions of key prognostic factors were in the main well reported, however some studies had many potential prognostic factors (any demographic or clinical characteristic included in a multivariable analysis), and in these cases there was generally a lack of detail for how these were defined. Given the lack of reporting of missing data, any methods of imputation of missing data were rarely reported.

### Outcome measurement

Most of the included studies used clear definitions of each outcome measured, with details of laboratory tests and diagnostic thresholds clearly described. However, the timing of occurrence of the outcome was mostly not reported, or only reported descriptively, precluding evidence-synthesis on the timing of the outcomes.

### Adjustment for other prognostic factors

The following list of other prognostic factors (adjustment variables) was considered by the review authors to require inclusion in multivariable analyses for these studies—age, sex, body mass index (BMI), alcohol intake, concomitant immune-suppressing drugs, disease duration, chronic kidney disease and other comorbidities. While the inclusion of all these variables was not an expectation, the majority (42 studies) were considered to include either no or only a selective limited choice of adjustment (existing prognostic) factors in the multivariable model. Some studies only included variables in their multivariable analysis when the unadjusted effect of those variables was significant. It was also often unclear whether there was missing adjustment factor data and/or how these were handled, with multiple imputation only reported in four studies.^24 34 35 51^

### Statistical analysis

Selective reporting of only significant results occurred in 27 studies and where this was the case, prognostic effect estimates were consequently not available for all variables leading to potential reporting bias.

## Study findings

Several patient and treatment factors were shown to be associated strongly with an increase or decrease in the risk of liver, blood or kidney adverse events in patients taking conventional or biological immune-suppressing drugs . Evidence found to be very low quality is only reported in the supplementary materials (online supplemental tables S4a,b–21a,b). A narrative synthesis of the results prioritising at least low-quality evidence is presented here and details outlined in tables 1–3.

**Table 1:** Summary of GRADE judgements: prognostic factors for hepatotoxicity, cytopenia and nephrotoxicity in those prescribed methotrexate

Prognostic factor	Summary of findings	Quality of evidence	Reason for up or down grading
Hepatotoxicity			
Age	No evidence of increased risk. Evidence from 11 studies^18–20 22 29 32 33 36 38 42 43^ with 7123 participants.	Low	
Alcohol	Evidence of increased risk with excess alcohol consumption from three studies^21 24 27^; but no evidence of association for any alcohol intake from three studies.^20 22 38^	Moderate	Up one each for large effect size, dose response. Down one for inconsistency.
Smoking	No evidence of increased risk. Evidence from seven studies^20–22 24 32 38 43^ with 44 801 participants.	Low	
Diabetes	Evidence of increased risk. Evidence from five studies^20 24 32 37 38^ with 42 600 participants.	Moderate	Up two for large effect size, down one inconsistency.
Comorbidity composite score*	Evidence of increased risk. Evidence from two studies^24 37^ with 42 237 participants with aHR (95% CI) 1.12 (1.01 to 1.24) and aOR (95% CI) 1.90 (1.00 to 3.60), respectively.	Low	
Disease duration	No evidence of increased risk from three studies^22 32 36^ with 925 participants.	Low	
Disease severity	No evidence of increased risk. Evidence from four studies^22 32 36 42^ with 1214 participants.	Low	
Disease type	Evidence of increased risk with psoriasis compared with RA. Evidence from four studies^18 24 37 41^ with 42 324 participants. One of the studies^18^ found no evidence of association for RA compared with PsO.	Moderate	Up two for large effect size, down one inconsistency.
Elevated liver enzymes	Evidence of increased risk. Evidence from six studies^20 22 33 37 38 42^ with 5931 participants.	High	Up two for large effect size.
Liver disease	Evidence of increased risk from four studies^21 22 32 33^ with 5751 participants.	High	Up two for large effect size and dose response, down one inconsistency.
Serology	No evidence of increased risk. Evidence from six studies^20 22 32 36 38 42^ with 2629 participants.	Low	
Inflammatory markers	No evidence of increased risk from three studies^22 32 42^ with 1076 participants.	Low	
Folate supplementation	Evidence of reduced risk. Evidence from three studies^26 30 37^ with 1551 participants.	High	Up two for large effect size.
Leflunomide	Evidence of increased risk when combined with methotrexate. Evidence from two studies^21 36^ with 2242 participants.	Moderate	Up two for large effect size and dose response, down one inconsistency.
Anti-TNF	No evidence of increased risk with anti-TNF combined with methotrexate. Evidence from four studies^21 22 32 37^ 3550 patients.	Low	
Methotrexate dose	No evidence of increased risk. Evidence from seven studies^18 20–22 32 36 37^ with 4843 participants.	Low	
Cytopenia			
Liver disease, elevated liver enzymes	Evidence of increased risk from one study^31^ with 175 participants (HR (95%CI) 5.83 (1.21 to 28.06)).	Low	Up two for large effect size and dose-response, down one inconsistency and single study.
Nephrotoxicity			
NSAIDs	Evidence of increased risk with co-prescription of NSAIDs from one study^40^ with 21 536 participants (aHR (95% CI) 2.04 (1.14 to 3.66)).	Moderate	Up two for large effect size, down one single study.

*Charlson Comorbidity Score used in both studies.

aHR, adjusted HR; aOR, adjusted OR; GRADE, Grading of Recommendations Assessment, Development and Evaluation; NSAIDs, non-steroidal anti-inflammatory drugs; PsO, psoriasis; RA, rheumatoid arthritis; TNF, tumour necrosis factor.

**Table 2:** Summary of GRADE judgements: prognostic factors for hepatotoxicity and cytopenia in those prescribed thiopurines

Prognostic factor	Summary of findings	Quality of evidence	Reason for up or down grading
Hepatotoxicity			
Age	Evidence of increased risk from four studies^48 50 59 62^ including 20 337 participants that age increases the risk of elevated liver enzymes with age >50 years, and ≥60 years associated with hepatotoxicity with aOR (95% CI) 4.5 (2.2 to 9.3)^62^ aHR (95% CI) 2.07 (1.72 to 2.50) in.^48^	Moderate	Up two for large effect size, down one inconsistency.
Male sex	Evidence of increased risk from six studies^48–50 58 59 62^ with 25 400 participants.	Moderate	Up two for large effect size, down one inconsistency.
Smoking	No evidence of increased risk. From three studies^50 58 59^ with 1569 participants.	Moderate	
ALT elevation	Evidence of increased risk from one study^70^ with 305 participants aOR (95% CI) 3.85 (1.80 to 8.25).	Moderate	Up two for large effect size, down one single study.
Other immunosuppressant	No evidence of increased risk from four studies^50 59 62^ and,^58^ including 3181 participants.	Low	
Thiopurine type	Evidence of increased risk from three studies,^48 58 62^ including 21 032 participants that mercaptopurine carries a higher risk than azathioprine with aHR (95% CI) 1.71 (1.34 to 2.17) in,^48^ aHR (95% CI) 1.46 (1.15 to 1.85) in^58^ OR (95% CI) 2.14 (1.06 to 4.26) in.^62^	High	Up two for large effect size.
Dose	No evidence of increased risk from two studies,^59 62^ including 480 participants.	Low	
Cytopenia			
Female sex	Evidence of increased risk for female sex. Evidence from eight studies^46–49 57–60^ with 28 888 participants.	Low	Up one for large effect size in some, down one inconsistency.
Smoking	No evidence for increased risk from four studies^46 47 58 59^ with 2972 participants.	Low	
Disease activity	No evidence for increased risk. Evidence from four studies^47 57–59^ with 2340 participants.	Low	
Mercaptopurine	Evidence of increased risk compared with azathioprine. From four studies^49^ (aHR (95% CI) 5.00 (2.50 to 11.00)),^48^ (aHR (95% CI) 1.86 (1.55 to 2.24)),^58^ (HR (95% CI) 1.02 (0.81 to 1.29)) and^47^ (aHR (95% CI) 2.61 (1.39 to 4.88)), including 25 388 participants.	High	Up two for large effect size.
Poor thiopurine metaboliser	Evidence of increased risk from four studies,^46 52 53 57^ including 2823 participants.^47^ (n=695) showed no evidence of increased risk but they only included patients with wild type TPMT genotype.	High	Up two for large effect size, up one dose response. No downgrade for^47^ due to selection bias on this exposure and outcome.

aHR, adjusted HR; ALT, alanine transferase; aOR, adjusted OR; GRADE, Grading of Recommendations Assessment, Development and Evaluation; TPMT, thiopurine methyltransferase.

**Table 3:** Summary of GRADE judgements: prognostic factors for hepatotoxicity and renal function in those prescribed anti-TNF alpha

Prognostic factor	Summary of findings	Quality of evidence	Reason for grading up or down
Hepatotoxicity			
BMI	Evidence for increased risk. Evidence from two studies^66 75^ with 1424 participants.	Low	
Comorbidities	Evidence for increased risk from two studies^66 75^ with 1424 participants.	Moderate	Inconsistency downgrade one, large effect size upgrade two.
Liver disease, elevated liver enzymes	Evidence for increased risk from three studies^64–66^ with 731 participants.	High	Large effect.
Serology	No evidence for risk from two studies^66 75^ with 1424 participants.	Low	
Other drugs	No evidence of increased risk with co-prescription of NSAIDs, Statins, TB prophylaxis from three studies^64 66 75^ with 1424 participants.	Low	

BMI, body mass index; GRADE, Grading of Recommendations Assessment, Development and Evaluation; NSAIDs, non-steroidal anti-inflammatory drugs; TB, tuberculosis; TNF, tumour necrosis factor.

Tables 1–3 summarise the GRADE judgements for prognostic factors for hepatotoxicity, cytopenia and nephrotoxicity by drug type (MTX, thiopurines and anti-TNFs, respectively). The results are presented below by adverse event type.

### Hepatotoxicity

#### Methotrexate

There was high-quality evidence that baseline elevated liver enzymes^20 22 33 37 38 42^ are associated with hepatotoxicity, and folate supplementation^26 30 37^ is associated with reduced risk of hepatotoxicity. There was moderate-quality evidence that increased risk of hepatotoxicity is associated with excessive alcohol consumption,^20–22 24 27 38^ diabetes,^20 24 32 37 38^ pre-existing liver disease,^21 22 32 33^ PsO±PsA (compared with RA)^18 24 37 41^ and concurrent leflunomide^21 22 36^ treatment. There was low-quality evidence that Charlson Comorbidity Index^24 37^ was associated with hepatotoxicity. There was low-quality evidence that age,^18–20 22 29 32 33 36 38 42 43^ smoking,^20–22 24 32 38 43^ auto-antibodies associated with RA,^20 22 32 36 38 42^ disease severity/activity,^22 32 36 42^ inflammatory markers,^22 32 42^ disease duration,^22 32 36^ MTX dose^18 20–22 32 36 37^ and concomitant anti-TNF-alpha drugs^21 22 32 37^ were not associated with hepatotoxicity.

#### Anti-TNF

There was high-quality evidence of baseline liver enzyme elevations^64–66^ being associated with hepatotoxicity. There was low-quality and moderate-quality evidence from two studies of an increased risk of hepatotoxicity with increased BMI and comorbidities, respectively.^66 75^ There was low-quality evidence of no increased risk of hepatotoxicity with positive antinuclear antibody or rheumatoid factor (ANA or RF).^66 75^ Prescription of other non-steroid sparing drugs were not associated with hepatotoxicity, low-quality evidence from three studies.^65 66 75^

#### Thiopurines

There was moderate-quality evidence that pre-existing alanine transferase elevation,^70^ high-quality evidence that use of mercaptopurine rather than AZA^48 58 62^ increased the risk of hepatotoxicity. There was moderate-quality evidence that age^48 50 59 62^ and male sex^48–50 58 59 62^ increased and smoking^50 58 59^ did not increase the risk of hepatotoxicity.

There was low-quality evidence that disease activity,^48 58 74^ concomitant biological and non-biological immune suppressing drug therapy,^50 58 59 62^ AZA dose^59 62^ *were* not associated with hepatotoxicity.

There was moderate-quality evidence from one study that included patients with IBD treated with different drugs (5-aminosalicylates, corticosteroids, AZA, anti-TNF-alpha or none) that liver steatosis was associated with hepatotoxicity.^74^

### Cytopenia (including neutropenia)

#### Methotrexate

There was low-quality evidence that chronic liver disease^31^ increased cytopenia risk.

#### Anti-TNF

There was low-quality evidence for no association between increasing age, sex or inflammatory disease type and neutropenia.^63 67^ There was low-quality evidence of increased risk of cytopenia from previous neutropenia, and reduced risk with increased baseline neutrophil count.^67^ There was moderate-quality evidence that leucopaenia or low neutrophil count at baseline were associated with increased risk of neutropenia in those prescribed conventional DMARDs +/− biologics.^23^

#### Thiopurines

There was high-quality evidence that mercaptopurine use was associated with an increased risk of cytopenia compared with AZA.^47–49 58^ There was high-quality evidence that poor thiopurine metabolisers (based on thiopurine methyltransferase/nudix hydrolase (TPMT/NUDT) genotype±enzyme intermediate or low activity^46 52 53 57^) had increased risk of cytopenia. There was low-quality evidence that female sex^46–49 57–60^ was associated, and current smoking^46 47 58 59^ and disease activity^47 57–59^ were not associated with cytopenia.

There was moderate-quality evidence from one study that included patients with autoimmune-rheumatic disease treated with different drugs that low baseline leucopaenia and low neutrophil count were associated with neutropenia.

### Nephrotoxicity

There was moderate-quality evidence that concomitant use of non-steroidal anti-inflammatory drugs (NSAIDs)^40^ and risk factors for renal function decline^72^ were prognostic for nephrotoxicity in patients prescribed MTX and anti-TNF-alpha, respectively.

### Composite toxicity (treatment discontinuation with cytopenia, acute kidney injury or elevated liver enzymes)

There was moderate-quality evidence that epilepsy and blood test abnormalities in the first few months of shared care prescription^34^ was associated with composite toxicity in patients prescribed leflunomide and moderate-quality evidence that chronic kidney disease stage 3^35^ was associated with composite toxicity in patients prescribed mycophenolate mofetil.

## Discussion

The review retrieved 56 studies published in 58 papers from 1995 to January 2023 that reported potential prognostic factors for common adverse events (liver, blood and kidney) in patients with a range of conditions who were prescribed immune-suppressing drugs. Most of these were designed as retrospective cohort studies. The most consistent finding was that, across drug types, baseline elevated liver enzymes were associated with increased risk of subsequent hepatotoxicity after adjusting for many other prognostic factors. The largest quantity of evidence related to prognostic factors associated with an increased risk of hepatotoxicity, with much of this from low or moderate quality evidence. The main reasons for downgrading evidence was single-study, imprecision and inconsistency. Factors shown to increase risk included BMI, age, comorbidities and the specific drug prescribed or use of concomitant drugs. These findings varied by drug type (anti-TNFs, MTX or thiopurines). Conversely, there was strong evidence that supplementation of folates was shown to reduce risk in patients prescribed MTX. Several factors were shown to predict an increased risk of cytopenia. These included previous neutropenia, comorbidities and poor metaboliser based on TPMT/NUDT genotype±enzyme intermediate or low activity. Little evidence was identified for prognostic factors for nephrotoxicity, and the quality was low, but included concomitant use of NSAIDs in those prescribed MTX.

The review was broad, with a focus on identifying risk factors for liver, blood and kidney adverse events. The strengths of this systematic review are its comprehensive inclusion of evidence that spans all relevant immune-suppressing drugs prescribed to patients with a range of conditions and up-to-date searches which retrieved evidence as recently as January 2023. Furthermore, it was conducted and reported following international guidelines by a team of highly experienced reviewers and clinicians. The limitations are that, while the search was sensitive and extensive, some relevant studies might still have been missed given the large number of therapies and populations; and despite only including studies with at least 200 participants overall, some included studies still had small numbers of participants in trial arms with relevant therapies resulting in low event rates for the outcomes of interest. Studies of certain populations may have been disproportionately excluded due to generally lower sample sizes, for example, SLE. Several studies included a small number of patients with SLE alongside patients with other inflammatory conditions. Thus, while it may be possible to extrapolate the results of the review to SLE, this should be done with caution and with a low degree of certainty. Furthermore, due to the heterogeneity in the included studies, the results of the review are pooled by drug type/adverse event without disaggregating by condition. Any differences between conditions have therefore not been explored. The evidence base itself was extensive and the risk of bias was generally low or moderate according to QUIPS assessments. However, the included studies’ available data and analyses for the outcomes of interest were relatively limited, with the result that quality of the evidence was assessed as low or very low according to the GRADE criteria, except for findings for prognostic factors for elevated liver enzymes. Prognostic factor findings were mainly assessed as very low quality, and this was in the main due to data being derived from small single studies, or where this was from multiple studies there was heterogeneity in outcomes, study designs, cut points used to describe prognostic factors and populations studied which also prevented meta-analysis. We did not identify any studies where prognostic factors of combination therapy were specifically addressed and the findings of this study should be extrapolated to combination therapies with caution.

## Conclusion

Patients prescribed immune-suppressing drugs are, in general, at higher risk of liver, blood and kidney adverse events if they have a prior history of or baseline blood test abnormalities, if they have comorbidities, or if they have tested positive for indicators of poor metaboliser activity for thiopurines. Identifying patients at the earliest opportunity who are at increased risk due to these factors could potentially help to reduce the risk of adverse events and ensure blood test monitoring is appropriately adjusted.

## Data Availability

All data relevant to the study are included in the article or uploaded as supplementary information. Extracted data from included studies is provided in the supplementary files.
