# Supplementary material for: Prognostic factors for liver, blood and kidney adverse events from glucocorticoid sparing immune-suppressing drugs in immune-mediated inflammatory diseases: a prognostic systematic review
Source: RMD Open. 2024 Jan 10;10(1):e003588. doi: 10.1136/rmdopen-2023-003588 (PMC10806492; doi:10.1136/rmdopen-2023-003588)
Supplement: Supplementary data [file rmdopen-2023-003588supp001.pdf]

Table of Contents

Supplementary methods: Search strategy 3
Supplementary methods: Study selection criteria........ 4
Table S1: Characteristics of included studies 5
Table S2a: Outcome characteristics and frequency of events for included studies - thiopurines: 17
Table S2b: Outcome characteristics and frequency of events for included studies – Anti-TNFs: 21
Table S2c: Outcome characteristics and frequency of events for included studies – DMARDs: 23
Table S2d: Outcome characteristics and frequency of events for included studies – various drugs 28
Table S3: Summary of QUIPs ratings for included studies by drug class 30
Supplementary results: differences in outcomes. 32
Table S4a: Prognostic factors for cytopenia and nephrotoxicity in those prescribed methotrexate 34
Table S4b. Summary of GRADE judgements: Prognostic factors for cytopenia and nephrotoxicity in those prescribed methotrexate 35
Table S5a: Demographic prognostic factors for hepatotoxicity in those prescribed methotrexate 37
Table S5b. Summary of GRADE judgements: Demographic prognostic factors for hepatotoxicity in those prescribed methotrexate 39
Table S6a: Lifestyle prognostic factors for hepatotoxicity in those prescribed methotrexate 40
Table S6b. Summary of GRADE judgements. Lifestyle prognostic factors for hepatotoxicity in those prescribed methotrexate 41
Table S7a: Comorbid prognostic factors for hepatotoxicity in those prescribed methotrexate 41
Table S7b. Summary of GRADE judgements: Comorbid prognostic factors for hepatotoxicity in those prescribed methotrexate 42
Table S8a: Liver, renal and serologic prognostic factors for hepatotoxicity in those prescribed 44
Table S8b: Liver, renal and serologic prognostic factors for hepatotoxicity in those prescribed methotrexate 45
Table S9a: Disease severity and activity related prognostic factors for hepatotoxicity in those prescribed methotrexate 47
Table S9b. Summary of GRADE judgments: Disease severity and activity related prognostic factors for hepatotoxicity in those prescribed methotrexate 48
Table S10a: Treatment related prognostic factors for hepatotoxicity or nephrotoxicity in those prescribed methotrexate 49
Table S10b. Summary of GRADE judgements: Treatment related prognostic factors for hepatotoxicity or nephrotoxicity in those prescribed methotrexate 51
Table S11a: Prognostic factors for hepatotoxicity and renal function in those prescribed anti-TNF alpha 52
Table S11b. Summary of GRADE judgements: prognostic factors for hepatotoxicity and renal function in those prescribed anti-TNF alpha 55
Table S12a. Prognostic factors for cytopenia 57
Table S12b. Summary of GRADE judgements: prognostic factors for cytopenia and neutropenia in patients prescribed anti-TNF alpha. 58
Table S13a: Prognostic factors for composite toxicity (myelotoxicity and/or hepatotoxicity) in people with inflammatory bowel disease prescribed thiopurines. 59
Table S13b: Summary of GRADE judgements: Prognostic factors for composite toxicity (myelotoxicity and/or hepatotoxicity) in people with inflammatory bowel disease prescribed thiopurines. 59
Table S14a: Prognostic factors for cytopenia in those prescribed thiopurines........ 60

Table S14b. Summary of GRADE judgements: Prognostic factors for cytopenia in inflammatory bowel disease prescribed thiopurines 63
Table S15a: Prognostic factors for hepatotoxicity in those prescribed thiopurines 65
Table S15b. Summary of GRADE judgements: Prognostic factors for hepatotoxicity in inflammatory bowel disease patients prescribed thiopurines 67
Table S16a: Prognostic factors for either cytopenia, nephrotoxicity, or elevated liver enzymes* in people with inflammatory bowel disease prescribed azathioprine 69
Table S16b: Prognostic factors for either cytopenia, nephrotoxicity, or elevated liver enzymes in people with inflammatory bowel disease prescribed azathioprine 69
Table S17a: Prognostic factors for acute kidney injury in inflammatory bowel disease patients prescribed thiopurines 70
Table S17b. Summary of GRADE judgements: Prognostic factors for acute kidney injury in inflammatory bowel disease patients prescribed thiopurines 70
Table S18a: Prognostic factors for either cytopenia, acute kidney injury, or elevated liver enzymes toxicity in people with autoimmune rheumatic diseases prescribed Leflunomide 71
Table S18b: Prognostic factors for either cytopenia, acute kidney injury, or elevated liver enzymes toxicity in people with autoimmune rheumatic diseases prescribed Leflunomide 71
Table S19a: Prognostic factors for either cytopenia, acute kidney injury, or elevated liver enzymes in people with autoimmune rheumatic diseases prescribed Mycophenolate mofetil 73
Table 19b: Prognostic factors for either cytopenia, acute kidney injury, or elevated liver enzymes in people with autoimmune rheumatic diseases prescribed Mycophenolate mofetil 73
Table S20a: Prognostic factors for hepatotoxicity (alanine transaminase increase) in inflammatory bowel disease patients prescribed different drugs*. 74
Table S20b. Summary of GRADE judgements: Prognostic factors for hepatotoxicity (alanine transaminase increase) in inflammatory bowel disease patients prescribed different drugs 74
Table S21a: Prognostic factors for neutropenia in Rheumatoid Arthritis (RA) patients prescribed any disease modifying anti-rheumatic drugs including biologics 75
Table S21b. Summary of GRADE judgements: Prognostic factors for neutropenia in RA patients prescribed any DMARDs including biologics 75
Table S22. Summary of prognostic factors for liver, blood and kidney adverse events in patients taking immune-supressing or biologic drugs 76

Supplementary methods: Search strategy

EMBASE MEDLINE Ovid search 5th January 2023*

1 exp Arthritis, Rheumatoid/ or rheumatoid arthritis.mp. or inflammatory bowel disease*.mp. or exp Inflammatory Bowel Diseases/ or exp Crohns disease/ or exp Colitis, Ulcerative/ or exp Psoriasis/ or (psoriasis adj2 arthriti*).mp. or exp Spondylitis, Ankylosing/ or ankylosing spond#litis.mp. or exp Lupus Erythematosus, Systemic/ or systemic lupus.mp. or reactive arthritis.mp. or Arthritis, Reactive/

2 limit 1 to (embase or medline)

3 prognosis.sh. or diagnosed.tw. or cohort:.mp. or predictor:.tw. or death.tw. or exp models, statistical/

4 limit 3 to (embase or medline)

5 methotrexate.mp. or exp Methotrexate/ or azathioprine.mp. or exp Azathioprine/ or 6-mercaptopurine.mp. or sulfasalazine.mp. or mycophenolate mofetil.mp. or exp Mycophenolic Acid/ or exp Sulfasalazine/ or leflunomide.mp. or exp Leflunomide/ or etanercept.mp. or exp Etanercept/ or adalimumab.mp. or exp Adalimumab/ or infliximab.mp. or exp Infliximab/ or golimumab.mp. or certolizumab.mp. or (mesalazine or olsalazine or balsalazide).mp. or exp Mesalamine/ or 5-aminosalicylic acid.mp.

6 limit 5 to (embase or medline)

7 (safe or safety or side effect* or undesirable effect* or treatment emergent or tolerability or toxicity or adrs or ((adverse or toxic*) adj2 (effect or effects or reaction or reactions or event or events or outcome or outcomes or liver or kidney))).tw.

8 limit 7 to (embase or medline)

9 (ae or co or de).fs.

10 limit 9 to (embase or medline)

11 8 or 10 4493248

12 2 and 4 and 6 and 11

13 limit 12 to yr="2020 - 2023"

*This was an identical update based on the original search conducted September 2020

Supplementary methods: Study selection criteria

	Inclusion criteria	Exclusion criteria
**P**opulation	Adults aged ≥18 years<br>[A] Diagnosed with either rheumatoid arthritis, inflammatory bowel disease, psoriasis +/- arthritis, ankylosing spondylitis, systemic lupus erythematosus, or reactive arthritis, **and**<br>[B] treated with either methotrexate, azathioprine, 6-mercaptopurine, 5-acetyl salicylate, sulfasalazine, mycophenolate mofetil, leflunomide, or anti-TNF-α agents namely etanercept, adalimumab, infliximab, golimumab, certolizumab.	Studies on participants with autoimmune hepatitis, small or large vessel vasculitis, glomerulonephritis, systemic sclerosis, primary Sjogren's syndrome, cancer, multiple-sclerosis, solid-organ transplantation. Studies of cyclosporine, tacrolimus, sirolimus as monotherapy.
**I**ndex / **C**omparator prognostic factors	Patient, disease and treatment factors associated with liver, blood or kidney adverse events.<br><br>There is no single index prognostic factor of interest. Any patient factor e.g. age, sex, comorbidities such as diabetes, lifestyle, or index condition (rheumatoid arthritis, lupus etc.) or concomitant treatment (e.g. methotrexate, azathioprine, sulfasalazine, adalimumab etc.) factors was of interest.<br><br>Comparator will be absence of risk factors of interest or the lowest category of risk factors.	Prognostic factors associated with adverse events that cannot be detected by routine monitoring will be excluded.
**O**utcomes	Adverse Events: liver, blood and kidney adverse events with or without drug withdrawal or interruption due to above	Adverse events that cannot be detected by routine monitoring were excluded.
**T**iming	Where reported, time-points and duration of data collection: 3, 6, 9, 12 months, and annually there-after	Not Applicable
**S**etting, study design	(1) Primary or secondary care;<br>(2) Longitudinal designs including cohort studies (primarily), case-control studies, randomized controlled trials, controlled trials;<br>(3) ≥200 participants*	Case series, case reports, grey literature, conference abstracts
Date	1st January 1995 onwards*	Pre-1995

* A revision was applied to the original protocol due to the scale of the literature encountered: inclusion was limited to only 1995 onwards. A requirement of ≥200 total study participants was also applied in order to address the risk of small study effects.

Table S1: Characteristics of included studies

Study/design<br><br>(Location)	N	Male : female, n (%)	Age (years), mean (SD)/ median (IQR)	Drug	Outcome	Incidence, adjustment or discontinuation	Follow-up/Timing of tests	Details of tests
**MTX**								
**RA**								
Amital 2009<br><br>Retrospective cohort study (Israel)	N = 809 of which n=119 RA, n=690 PsO	RA – 59.9%:40.1%<br>PsO – 48.3%:51.7%	Mean age:<br><br>RA – 59.9 years +/-14.2<br><br>PsO – 52.6 years +/- 15.2	Methotrexate (MTX)	Hepatotoxicity	Incidence	Follow-up:<br>Mean follow-up<br>RA – 843 days,<br>PsO – 883 days	Laboratory liver function tests
Bologna 1996<br><br>Prospective cohort study (Spain)	N = 469 <65years n=416, ≥65years n=53	<65 years: 72/344<br>≥65 years: 14/39	Mean age (SD)<br><br><65 years group: 48.7 +/- 11 years; ≥65 years group: 68.8 +/- 3.1 years	MTX	Elevation of transaminases<br><br>Haematological (macrocytosis, leucopenia, thrombopenia)	Incidence	*Duration of study:* patients treated between 1985 and 1994 were evaluated in November 1994.<br><br>*Timing:* N/R	NR
Curtis 2010<br><br>Retrospective cohort study (USA)	N = 2104	MTX + LEF 19.4%:80.6%<br>MTX 26.0%:74.0%<br>LEF 21.4% :78.6%<br>No MTX or LEF 29.1%:70.9%	Mean age (SD)<br><br>MTX + LEF: 58.8 (12.8)<br>MTX: 59.1 (13.3)<br>LEF: 59.4 (14.4)<br>No MTX or LEF: 56.9 (14.9)	Methotrexate (MTX); Leflunomide (LEF); MTX + LEF or other non-biologic DMARD combinations	Hepatotoxicity	Incidence	*Timing:* Mean time between study visits where blood was drawn approx. 5 months	Laboratory liver function tests
Dirven 2013<br>BeST trial/RCT reanalysed as a cohort (Netherlands)	N = 498	162:336	Mean age 54 years (SD 14)	Methotrexate	Elevated liver enzymes	Incidence (N= measurements)	*Follow-up:*<br>5 years<br><br>*Mean duration of MTX use*: 2.8 years<br><br>*Timing:*<br>Every 3 months	Laboratory tests
Hayashi, 2020<br><br>Retrospective cohort (Japan)	n=502	108 (22%): 394 (78%)	Mean (SD):<br>63 (13) years	MTX	Change in eGFR during the previous 1 year	Incidence	*Follow-up:*<br>1 year observation period (data collected from the most recent 1 year period of exposure).<br><br>*Timing of tests*:<br>NR	WBC count, hemoglobin (Hb), platelet count, serum creatinine (s-Cr) levels, C-reactive protein (CRP) level

**Study/design**<br><br>**(Location)**	**N**	**Male : female, n (%)**	**Age (years), mean (SD)/ median (IQR)**	**Drug**	**Outcome**	**Incidence, adjustment or discontinuation**	**Follow-up/Timing of tests**	**Details of tests**
Hoekstra 2003<br><br>RCT (Netherlands)	N = 411	+folate 85:189; placebo 37:100	Mean age (SD) folate group: 55.4 years (12.7); placebo group: 57.2 years (12.7)	MTX	Severe hepatotoxicity	Incidence, discontinuation	*Follow-up:* 48 weeks	Alanine aminotransferase
Humphreys 2017<br><br>Retrospective cohort study (UK)	N = 11,839	29%:71%	Median age: 61 years (IQR 51-70)	MTX	Transaminitis	Incidence	*Follow-up:* study period was 1987 to 2016, unless patient was censored earlier<br><br>*Timings:* 6 times every twelve months	AST/ALT
Jiang 2012<br><br>RCT (China)	N = 505<br><br>Western Medicine n=25; Chinese Medicine n=254	Western Medicine: 38:216<br>Chinese Medicine: 42:209	Mean age:<br>WM: 47.1 years +/- 12.11<br>CM: 49.8 years +/- 11.49	MTX	Hepatic adverse events	Incidence	*Follow-up:* treatment was for 24 weeks<br><br>*Timings:* Baseline, then every 4 weeks	Liver chemistry
Karlsson Sundbaum 2019, 2021<br><br>*Cavalli 2022<br><br>Retrospective cohort study (Sweden)	N = 358	Discovery cohort (principal cohort for evaluation and anlaysis) n=198; Replication cohort n=160<br><br>70:143<br><br>* Elevated ALT vs no elevated ALT: 33 (9%) vs 97 (30) : 30 (91) vs 218 (70)	Mean (SD) age at diagnosis: 54.8 years (14.3)<br><br>Mean (SD) age at treatment start: 55.9 (13.8)<br><br>*Elevated ALT vs no elevated ALT 55.8 (11.3) vs 56.7 (14.3)	MTX	Elevated liver enzymes<br><br>(within the first 6 months)		*Follow-up:* Mean 225 weeks (SD 129)<br>*Follow-up:* Minimum 6 months of treatment<br><br>*Timings:* Every 14 days for 3 months, followed by monthly testing for 3 months, and every 3 months thereafter	Laboratory ALT tests
Kent 2004<br><br>Retrospective cohort study (USA)	N = 481	147:334	Mean age at diagnosis: 47 years (SD 14)	MTX	Hepatic and haematologic abnormalities	Incidence (at least one elevation of ALT > 1.5 x ULN), discontinuation	*Follow-up:* Mean months monitored 58 (SD 38)<br><br>*Timings:* Mean measurements per year 8.4 (SD 3)	Laboratory tests according to Swedish guidelines, e.g. ALT and liver function tests

Study/design<br><br>(Location)	N	Male : female, n (%)	Age (years), mean (SD)/ median (IQR)	Drug	Outcome	Incidence, adjustment or discontinuation	Follow-up/Timing of tests	Details of tests
Mori 2020<br><br>Retrospective cohort study (Japan)	N = 289	70:219	Mean age 60.4 years (SD 59.0)	MTX	Persistent transaminitis	Incidence, discontinuation	*Follow-up:* Mean follow-up 58.3 months (95% CI: 54.2, 62.5)<br><br>*Timing*: every 4-8 weeks during MTX treatment	Laboratory tests
Sakthiswary 2014<br><br>Retrospective cohort with nested case-control study (Singapore)	N=1105<br><br>46 cases with NAFLD and transaminitis and 92 controls were included in the analysis.	Female<br>Case 37 (80.4%)<br>Control 72 (78.3%)	Mean (SD) Age Cases 54.28±9.50, Controls 60.45±10.650.487	MTX	Non-alcoholic fatty liver disease (NAFLD) with transaminitis	Discontinuation	*Follow-up:* Duration unclear.	NR
Sherbini 2021<br><br>Prospective cohort study (UK)	N=1069	34.7%:65.3%	Mean age (SD):<br><br>59.2 years (13.5)	MTX	Elevated liver enzymes	Incidence	*Follow-up:* 6 and 12 months	LFTs
Suzuki 2021<br><br>Prospective cohort study (Japan)	N = 2860 of which n = 1292 MTX ≥1 year; n= 1001 MTX < 1 year	24 week follow-up patients:<br>MTX ≥1 year 19.6%:80.4%<br>MTX < 1 year 31.1%:68.9%	Mean age (SD) (24 week follow-up patients):<br><br>MTX ≥1 year 58 (11.9) years<br>MTX < 1 year 54.4 (13.8 years)	MTX	Hepatotoxicity	Incidence	*Follow-up:* 6 and 12 months	ALT
Svanstrom 2018<br><br>Retrospective cohort study (Denmark)	N = 17,200	28.7%:71.3% (no concomitant NSAIDs)<br>29.4%:70.6% (concomitant NSAIDs)	Mean age (SD):<br><br>No concomitant NSAIDs 59.8 years (14.9)<br><br>Concomitant NSAIDs 57.8 (14.2)	MTX	Liver toxicity, acute renal failure, cytopenia	Incidence	*Follow-up:* 1 year (median 125 days IQR 63-226)	NR

Study/design (Location)	N	Male : female, n (%)	Age (years), mean (SD)/ median (IQR)	Drug	Outcome	Incidence, adjustment or discontinuation	Follow-up/Timing of tests	Details of tests
Verstappen 2010<br><br>Prospective cohort study (Netherlands)	N = 289	I group 31.5%:68.5%<br>C group 35.0%:65.0%	Mean age (SD):<br><br>I group – 54 years +/- 14<br><br>C group – 52 years +/- 15	MTX	Liver toxicity; Renal toxicity; haematological toxicity	Incidence (events)	*Follow-up:* 2 years<br><br>*Timing:* C-group once every 3 months, I group once every 4 weeks	NR
Yang 2022<br><br>Retrospective cohort study<br><br>(International)	N = 21, 452 (leucopenia)	With outcome event 22.3%:77.7%<br><br>Without outcome event 28.9%:71.1%	Mean age: N/R	MTX	Leucopenia	Incidence	*Eligibility criteria:* At least 365 days of observation prior to MTX	Laboratory tests for AST, ALT, ESR, Hb, creatinine
Nakafero, 2022b<br><br>Retrospective cohort study/ database (Clinical Practice Research Datalink [CPRD]) (UK)	n=1487 (derivation cohort)	508 (34.2%): 979 (65.8%)	57 (13) years	LEF	Drug discontinuation with abnormal blood test results	Incidence	*Follow-up:* 180 days after the first LEF prescription issued by the GP until the earliest of date of outcome, death, transfer out of the practice, date of last data collection from the practice, 5 years or 31 December 2019<br><br>1487 patients with 3140 person-years follow-up<br><br>*Timing of tests*: NR	FBC, UEC, LFT
**PsO**								
Zhu, 2022<br><br>Prospective cohort study (China)	n=1,440<br><br>MTX=309<br>Controls = 1,031	Across genotypes: 68.3–75: 31.7-25	Across genotypes: 46.6 (12.9)-54.1 (15.8) years	MTX	Hepatotoxicity<br><br>-ALT elevation;<br>-Abnormal hepatic function		*Follow-up:* At least 1 year<br><br>*Timing of tests:* Monthly	Liver function tests ALT, AST, direct bilirubin (DBIL), and total bilirubin (TBIL).
**RA; AS; PsO/PsA (mixed rheumatic and/or skin disease population)**								
Gelfand, 2021	N = 40,237	PsO: 54.7%: 45.3%; PsA: 42.7%: 57.3%; RA: 28.4%: 71.6%	Age at cohort entry, mean (SD), years:	MTX	Liver disease	Incidence	*Study period:* 18 years	Not reported

Study/design<br><br>(Location)	N	Male : female, n (%)	Age (years), mean (SD)/ median (IQR)	Drug	Outcome	Incidence, adjustment or discontinuation	Follow-up/Timing of tests	Details of tests
Retrospective cohort study/ database (Denmark)	PsO n=5687, PsA n=6520, RA n=28,030		PsO: 47.7 (17.2); PsA: 50.7 (13.7); RA: 59.7 (14.6)					
Schmajuk 2014<br><br>Retrospective cohort study (USA)	N = 659 of which 379 (57%) RA, 142 (22%) PsO/PsA, other 138 (21%)	638:21	Mean age:<br>71.3 years (SD 6.3)	MTX	Elevated transaminases	Incidence	*Follow-up:*<br>Mean follow-up 227 days (SD 147)	Liver function tests
Tilling 2006<br><br>Prospective cohort study (UK)	N = 619 of which n=550 RA, n=69 PsA	RA 137:413<br>PsA: 40:29	Mean age:<br>RA – 62 years (20-92)<br>PsA – 49 years (9-76)	MTX	Hepatic toxicity	Incidence, discontinuation	*Timing:* Baseline, then weekly until treatment stabilised, then every 2-3 months	FBC, ESR, CRP, LFT, renal profile.
Kwon 2018<br><br>Retrospective cohort study (South Korea)	N = 175	MTX 31:109<br>MTX + TMP-SMX 18:17	Mean age (SD):<br><br>MTX 54.3 years (+/- 13.4)<br>MTX + TMP-SMX 54.3 years (+/- 13.7)	MTX with or without a prophylactic dose of trimethoprim-sulfamethoxazole (TMP-SMX)	Cytopenia	Incidence	*Study duration:* 2005-2017<br><br>*Timing:* N/R	Laboratory tests
**SLE; RA; PsO; IBD; AS**								
Nakafero, 2022a<br><br><br>Retrospective cohort study/ database (Clinical Practice Research Datalink [Aurum]) (UK)	n=992<br><br>SLE (n= 467), RA (n = 248), PsO (n=168), IBD (n = 94), AS (n = 15)	309 (31.2%) : 683 (68.9%)	51.95 (17.12) years	Mycophenolate mofetil (MMF)	Drug discontinuation associated with abnormal or severely abnormal blood-test results	Incidence, adjustment, discontinuation	*Follow-up:*<br>Cohort exit was assigned as the earliest of date of the following outcomes: death, transfer out of the GP practice, last data collection from the GP practice, 5-year follow-up, or 31 December 2019<br><br>992 patients with 1885 person-years of follow-up<br><br>*Timing of tests*:<br>NR	FBC, UEC, LFT
**Anti-TNFs**								
**RA; AS; PsO/PsA (mixed); IBD**								

**Study/design**<br><br>**(Location)**	**N**	**Male : female, n (%)**	**Age (years), mean (SD)/ median (IQR)**	**Drug**	**Outcome**	**Incidence, adjustment or discontinuation**	**Follow-up/Timing of tests**	**Details of tests**
Chiu 2017<br><br>Retrospective cohort with nested case control study (Taiwan)	N = 407	108:260	Mean age, years (+/- SD):<br><br>Cases: 46.6 (+/- 15.9)<br><br>Controls: 52.2 (+/- 15.2)	Etanercept, Adalimumab, Golimumab. + DMARD cohort.	Liver enzyme elevation	Incidence	*Follow-up:* 12 months<br><br>*Timing:* Baseline then 6 months after start of treatment and then every three months	Serum ALT, Hepatitis B virus serostatus
Chiu 2018<br><br>Retrospective cohort study (Taiwan)	N=472	Anti-TNF NR: 305<br>DMARDs: 112:199	Age mean (range):<br>Biologic 52.1 (6.6-85.6)<br>DMARD 50.3 (16.9-82.5)	Etanercept, Adalimumab, Golimumab. DMARDs.	Liver enzyme elevation	Incidence	*At six months, then 3 monthly*	ALT
Hastings 2010<br><br>Retrospective cohort study (UK)	N = 367	106:261	Mean age, years:<br><br>Neutropenia: 56.8<br><br>Without: 57.5	Etanercept, adalimumab, infliximab	Neutropenia	Incidence	*Follow-up:* 12 months<br><br>*Timing:* Baseline, 2 weeks after starting TNF inhibitors, then every month	Neutrophil count, lymphocytes, monocytes, basophils, eosinophils
Madani, 2022<br><br>Retrospective cohort study (Saudi Arabia)	n=250	108 (43.2%): 142 (56.8%)	Median (IQR):<br>33.5 (24-48) years	ADA (38.4% of patients)<br>ETA (38%)<br>IFX (23.6%)	Abnormal laboratory results (timepoints)	Incidence	*Follow-up:*<br>24 months<br><br>*Timing of tests:*<br>Every 3 months	Complete blood count (CBC) with differential counts, liver function tests (LFTs),and renal function
**IBD**								
AlAskar 2020<br><br>Retrospective cohort study (Saudi Arabia)	n=281	Neutropenia vs no neutropenia (M:F)<br><br>45 (46.9%): 51 (53.1%)<br>vs<br>104 (56.2%):<br>81 (43.8%)	Neutropenia vs No neutropenia<br><br>32.90 (10.11)<br>vs<br>32.61 (9.46)	Neutropenia vs No neutropenia<br><br>ADA:<br>58 (60.4%) vs 2 (2.1%)<br>Certolizumab (CTZ):<br>36 (37.5%) vs 0 (0%)<br>IFX:<br>99 (53.5%) vs 4 (2.2%)<br>Golimumab:<br>81 (43.8%) vs 1 (0.5%)	Neutropenia	Incidence	*Follow-up:*<br>Up to 11 years<br><br>*Timing of tests*:<br>NR	Neutrophil count<br>WBC count

Study/design<br><br>(Location)	N	Male : female, n (%)	Age (years), mean (SD)/ median (IQR)	Drug	Outcome	Incidence, adjustment or discontinuation	Follow-up/Timing of tests	Details of tests
Shelton 2015<br><br>Retrospective cohort with nested case control study (USA)	N = 1753	Male:<br>56.3% (case), 68.7% (control)	Age (years):<br><br>Case: 32<br><br>Control 31	IFX, ADA, CTZ	Liver enzyme elevation	Incidence	*Follow-up:* Mean duration of follow-up of controls – 167 weeks<br><br>Mean time to first ALT elevation – 29 weeks<br><br>*Timing*: Prior to Anti-TNF initiation, then every 3-4 months	ALT levels
Parisi 2016<br><br>Retrospective cohort study with nested case control (UK)	N = 305 of which 176 treated with infliximab, 129 other drugs for IBD	164:141	Mean age: 40 +/- 14.7 years	IFX	Liver enzyme elevation	Incidence	*Follow-up*: 5 years<br><br>*Timing:* Baseline, every 8 weeks for infliximab, every 3-6 months for the remainder of the population	Full blood count and liver biochemistry
**AS**								
Choi 2020<br><br>Retrospective cohort study)<br>(South Korea)	N = 363	78:8 (elevated)<br><br>198:79 (normal)	Age at onset of TNFi:<br><br>Elevated: 37.1 years (13.1)<br><br>Normal: 37.9 (13.7)	ADA, IFX, ETA, GOL	Liver enzyme elevation	Incidence	*Study period:* 14 years<br><br>*Median duration of TNFi exposure before elevated AST/ALT*: 3.72 months	ALT, AST, alkaline phosphatase, bilirubin, antinuclear antibody, human leukocyte antigen-B27 erythrocyte sedimentation rate, C-reactive protein
Swart 2022<br><br>Prospective cohort study<br>(Netherlands)	N = 211	67.3%/32.7%	Mean age (years): 42.56 +/- 11.24	ADA, ETA	Decline in renal function	Level of renal decline over time	*Study period:* 9 years<br><br>*Treatment period*: 2 years<br><br>*Median follow-up time:* 156 (36-286) weeks	Laboratory data
**PsA**								

Study/design<br><br>(Location)	N	Male : female, n (%)	Age (years), mean (SD)/ median (IQR)	Drug	Outcome	Incidence, adjustment or discontinuation	Follow-up/Timing of tests	Details of tests
Kavanaugh 2007 (RCT) (USA)	N = 200	51%:49% (placebo) 71%:29% (IFX)	Mean age – years (SD):<br><br>Placebo: 46.5 (11.3)<br><br>Infliximab: 47.1 (12.8)	IFX	Liver enzyme elevation	Incidence	*Follow-up*: 1 year<br><br>*Timing:* Baseline, week 2, 6, and every 8 weeks until week 54	Laboratory tests for ALT/AST
**Thiopurines (AZA, 6-MP, 5-ASA, SZS)**								
**IBD**								
Achit 2022<br><br>Retrospective cohort / database study (France)	n=94,363<br><br>Without AKI n=93,670<br><br>With AKI n=693	Without AKI: 44,700(48%): 48,970 (52%)<br><br>With AKI: 207 (30%): 686 (70%)	Without AKI: 37.24 (17.23)<br><br>With AKI: 43.38 (20.81)	5-ASA	Acute Kidney Injury (AKI)	Incidence	*Follow-up:* Without AKI: mean 5.40 (SD 2.03) years<br><br>With AKI: mean 2.95 (SD 2.07) years<br><br>*Timing of tests*: NR	Occurrence of this event was identified in the database by using the ICD-10 codes for AKIs: N00, N01, N04, N10, N141, N142, N144, N17.
Banerjee 2020<br><br>Prospective cohort study (India)	n=935<br><br>AZA no leucopenia (n=854)<br><br>AZA-induced leucopenia (n=81)	AZA no leucopenia vs AZA-induced leucopenia (M:F)<br><br>517 (60.5%) vs 337 (39.5%): 48 (59.3%) vs 33 (40.7%)	AZA no leucopenia: 35.97 (12.79)<br><br>AZA-induced leucopenia: 33.96 (13.01)	AZA	Leukopenia	Incidence; discontinuation	*Follow-up:* Median 17 months (range 1-144)<br><br>*Timing of tests*: NR	White blood cell (WBC) count
Broekman 2017<br><br>TOPIC trial Case control nested in a RCT (Netherlands)	N = 695 (Cases 45, Controls 650)	301:385	Median years (IQR):<br>Leukopenia: 43 (28-51)<br>No leukopenia: 40 (26-53)	AZA, 6-MP	Leukopenia	Incidence; discontinuation; reduction; recovery without intervention	*Follow-up:* 20 weeks<br><br>*Timing:* Baseline, week 1, 2, 4, 6, 8, 20	WBC count
Calafat 2019<br><br>ENEIDA registry<br><br>Retrospective cohort study (Spain)	N = 19,630 of which 1888 (9.6%) began treatment aged 60 or over; and 15,477 aged between 18 and 50 years.	18-50 years of age: 7999:7478<br>Elderly: 1025:863	Median age at beginning of treatment – years (IQR):<br><br>Elderly: 66 (62-71);<br><br>18-50: 33 (26-40)	AZA, 6-MP	Myelotoxicity (anaemia; leukopenia; bone marrow suppression; lymphopenia); hepatotoxicity	Incidence	*Follow-up:* up to 10 years<br><br>*Timing*: not reported	Not reported

**Study/design**<br><br>**(Location)**	**N**	**Male : female, n (%)**	**Age (years), mean (SD)/ median (IQR)**	**Drug**	**Outcome**	**Incidence, adjustment or discontinuation**	**Follow-up/Timing of tests**	**Details of tests**
Chaparro 2013<br><br>ENEIDA registry Retrospective cohort study (Spain)	N = 3931	2044:1877	Mean age – years (SD): 43 (14)	AZA, 6-MP	Leukopenia; bone marrow suppression; hepatotoxicity	Incidence; Discontinuation	*Follow-up:* Median 44 months (range 0-420)<br><br>*Timing:* not reported	Not reported
Cheng 2022<br><br><br>Retrospective cohort (USA)	n=336	208 (61.9%): 128 (38.1%)	Mean: 40.2 (SD 13.3) years.	At baseline: 42% used 5-ASA, 6.3% immune-modulator, 4.5%, biologic.	Elevated liver enzyme	Incidence	*Follow-up:* Median: 49.9 (IQR: 19.9–93.5) months<br><br>*Timing of tests*: NR	Liver function tests
Coenen 2015 (TOPIC trial)<br><br>RCT (Netherlands)	N = 796 randomised	354:429	Mean age: 41.0 years (SD 15.8)	AZA, 6-MP	Haematologic adverse drug reactions (ADRs)	Incidence	*Follow-up:* 20 weeks<br><br>*Timings:* 1 week prior to start of study, then weeks 1, 2, 4, 6, 8 and 20	Blood sampling for TPMT genotyping and enzyme measurements - Leukocytes, thrombocytes, haemoglobin, liver enzymes (ALT/AST)
Fangbin 2012<br><br>Prospective cohort with cross-sectional analysis (China)	N = 199	133:66	Mean age – 31.80 +/- 13.40 years	AZA, 6-MP	Leukopenia; hepatotoxicity	Discontinuation	*Follow-up:* 48 weeks<br><br>*Timing:* Full blood count: weekly for 4 weeks then once per month<br><br>Liver function tests: once per month then every 3 months until they were measured 3 times	WBC count; Neutrophil count; Aspartate aminotransferase or alanine aminotransferase level; Serum amylase level
Houwen 2021<br><br><br>Retrospective cohort study, database registry	n=37,360	Allopurinol: 66% : 34%<br><br>No allopurinol: 45% : (55%	Allopurinol: 59 (16.9)<br><br>No allopurinol: 48 (19.5)	AZA, 6-MP	Hepatotoxicity<br><br>Myelotoxicity (includes leukopenia and thrombocyto-penia)	Incidence	Follow-up, mean (SD), years: Allopurinol: 3.6 (5.5)	NR

**Study/design (Location)**	**N**	**Male : female, n (%)**	**Age (years), mean (SD)/ median (IQR)**	**Drug**	**Outcome**	**Incidence, adjustment or discontinuation**	**Follow-up/Timing of tests**	**Details of tests**
(Netherlands)							No allopurinol: 3.5 (4.1)<br><br>*Timing of tests*: NR	
Kakuta 2018<br><br>Case control study (Japan)	N = 2627, of which 1291 had thiopurine usage	1647:980 (all) 819:472 (thiopurines)	Mean age – 40.6 years (range 3-89)	SZS, 5-ASA, Infliximab (IFX), Adalimumab (ADA), 6-MP, or AZA	Leukopenia; Liver dysfunction; Pancreatitis	Discontinuation or modification	*Study duration*: 2 years	Not reported
Kim 2010<br><br>Retrospective cohort study (South Korea)	N = 286	187:99	Mean age (SD) 25.7 (9.3)	AZA, 6-MP	Leukopenia	Discontinuation or reduction	*Follow-up: 12 months*<br><br>*Timing:* 1-2 week intervals for the first month, then 1 month later, then every 3 months	Complete blood counts and liver function tests
Kim 2017 Retrospective cohort study (South Korea)	N = 267	181:86	Median age at start of treatment 32 (14-71)	AZA	Leukopenia	Discontinuation or reduction	*Study duration*: 14 years	WBC count
Kreijne 2020<br><br>Retrospective cohort study (Netherlands)	N = 1132	500:632	Median age 37 years (range 26-49)	AZA, 6-MP	Myelotoxicity; hepatotoxicity	Discontinuation, dose reduction or additional therapy with allopurinol + reduced thiopurine dose	*Median follow-up* (cessation or censoring): 3.3 years (IQR 1.7-5.6)<br><br>Baseline = 1 year after initial thiopurine treatment	Laboratory toxicity as described for myelotoxicity and hepatotoxicity
Labidi 2020<br><br>Retrospective cohort with nested case-control study (Tunisia)	N = 210	98:112	Mean age of disease onset 29.8 +/- 11.4 years	AZA, 6-MP	Myelotoxicity; hepatotoxicity	Incidence	*Median follow-up*: 28.5 +/- 20 months<br><br>*Timing:* Baseline, 1, 2, 4, 12 weeks after commencement of treatment then quarterly.	Full blood count and liver function tests.
Ribaldone 2019<br><br>Retrospective cohort with nested	N = 200	116:84	Mean age at diagnosis (range): UC – 33 years	AZA	Leukopenia; pancreatitis; hepatitis	Discontinuation	Not reported	Genotyping TPMT

**Study/design (Location)**	**N**	**Male : female, n (%)**	**Age (years), mean (SD)/ median (IQR)**	**Drug**	**Outcome**	**Incidence, adjustment or discontinuation**	**Follow-up/Timing of tests**	**Details of tests**
case control study (Italy)			(13-59); CD – 30.5 (16-67)					
Wong 2017<br><br>TOPIC trial/RCT with nested case control study (Netherlands)	N = 270	117:153	Median age 40 years (range 18-81)	AZA, 6-MP	Hepatotoxicity	Incidence	*Follow-up:* 20 weeks<br><br>*Timing:*<br>1 week before initiation, and 1,2,4,6,8 and 20 weeks after initiation	Blood samples for 6-MMPR and 6-TGN collected 1 week after thiopurine initiation
**CD**								
Park 2016<br><br>Retrospective cohort with nested case control study (South Korea)	N = 964	693:271	Median age (range) at start of thiopurine therapy: early 27 (12-46); late 25 (15-74); no case: 24 (13-64)	AZA, 6-MP	Leukopenia	Early (before 8 weeks) and late (after 8 weeks) incidence	*Study period*: 23 years<br><br>*Timing:* Every two weeks for the first 8 weeks, then monthly for the following 1-2 months, then every 2-3 months	Blood leukocyte levels, DNA
**RA; IBD, SLE**								
Dickson 2022<br><br>Retrospective cohort study (USA)	n=1403	446 (32%): 957 (68%)	46.1 (15.8) years	AZA	Myelotoxicity	Discontinuation	*Follow-up:* Median: 19.1 (3.1–61.7) months<br><br>*Timing of tests:* NR	Not reported (NR)
**Cohorts prescribed different classes of drugs**								
**RA**								
Fragoulis 2018<br><br>Prospective cohort with nested case control study (UK)	N = 771	neutropaenia 11:47, no neutropenia 253:460	Mean age (SD):<br><br>Neutropaenia 57.7 (14.1);<br><br>No neutropaenia 58.8 (13.4)	MTX,<br>SZS,<br>Hydroxychloroquine<br>LEF,<br>AZA<br><br>Corticosteroids,<br>Anti-TNF-alpha<br>Abatacept<br>Tocilizumab<br>Rituximab	Neutropaenia	Incidence, discontinuation	*Follow-up:* median 18 months (range 6-48 months)<br><br>*Timings:*<br>Baseline then every 6 months	Laboratory tests: full blood count, biochemistry profile, acute phase reactants, rheumatoid factor, anti-Cyclic Citrullinated Peptide
**RA and IBD**								

Study/design<br><br>(Location)	N	Male : female, n (%)	Age (years), mean (SD)/ median (IQR)	Drug	Outcome	Incidence, adjustment or discontinuation	Follow-up/Timing of tests	Details of tests
Fraser 2022<br><br>Retrospective cohort study / database (UK)	n=4,163<br><br>n=3001 RA patients (MTX)<br><br>n=1162 IBD patients (AZA)	328,712 (46.8%): 373,553 (53.2%)<br><br>[overall study population, n=702,265]	51.9 (18.8) years [overall study population, n=702,265]	MTX<br><br>AZA	Lack of blood test abnormality	Incidence	*Follow-up:* 2 years (study duration)<br><br>Timing of tests: 3 months	ALT, WBC, and eGFR
**IBD**								
Koller 2017<br><br>Prospective cohort with nested case control study (Slovakia)	N = 251	122:129	Median age 39 (IQR 30.0-52.75)	Mesalamine; Antibiotic; Corticosteroids; AZA; anti TNF-alpha; combination of above.	Liver injury	Incidence – n=measurements	*Follow-up*: 1 year<br><br>*Timings:* Baseline, then every 3 months until 1 year. Aminotransferase recorded from records for 3 months prior to study entry.	Blood sampling for serum activities of ALT, AST, GGT, ALP and serum bilirubin concentration.
**PsA**								
Pakchotanon 2020<br><br>Prospective cohort with nested case control study (Canada)	N = 1061	145:111 (cases)<br><br>383:335 (controls)	Mean age at baseline (SD): 42.6 (11.9) (cases) 43.1 (13.5) (controls)	Non-biologic DMARDs, Anti-TNF	Liver abnormalities	Incidence and prevalence	*Timings*: 6 and 12 month intervals	Laboratory tests for liver enzymes

5-ASA=5-acetyl salicylate; AKI=Acute kidney injury; ALT=alanine transaminase; AST=aspartate transaminase; ALP=alkaline phosphatase; Anti-TNF: Anti-Tumour necrosis factor; AS=Ankylosing spondylitis; AZA=Azathioprine; CD=Crohn's disease; DMARD=Disease modifying antirheumatic drugs; eGFR= estimated glomerular filtration rate; FBC=Full blood count; GGT=Gamma glutamyl transferase; IBD=Inflammatory Bowel Disease (IBD); IQR=Interquartile range; LFT=Liver function test; MMF=Mycophenolate mofetil; MP=mercaptopurine; MTX=Methotrexate; SLE=Systemic lupus erythematosus; RA=Rheumatoid arthritis; N=number; PsO=Psoriasis; PsA=Psoriatic arthritis; RCT =Randomized controlled trial; SD=Standard deviation; SLE=Systemic lupus erythematosus; Rheumatoid arthritis (RA); SZS=Sulfasalazine; UC=Ulcerative colitis; UEC=Urea and electrolyte test; WBC=White blood cells

Table S2a: Outcome characteristics and frequency of events for included studies - thiopurines:

Study	Outcome	Definition of outcome	Incidence, adjustment or discontinuation	Follow-up/Timing of tests	Details of tests	Number (%) with outcome
**IBD**						
Achit 2022<br><br>n=94,363<br><br>Without AKI n=93,670<br><br>With AKI n=693	Acute Kidney Injury (AKI)	Patient hospitalization related to AKI	Incidence	*Follow-up:*<br>Without AKI: mean 5.40 (SD 2.03) years<br><br>With AKI: mean 2.95 (SD 2.07) years<br><br>*Timing of tests*:<br>NR	Occurrence of this event was identified in the database by using the ICD-10 codes for AKIs: N00, N01, N04, N10, N141, N142, N144, N17.	Occurrence n=693 (<1%)
Banerjee 2020	Leucopenia	WBC count 3.0 × 109/L.<br>Grades 2, 3 and 4 leucopenia were defined as WBC counts of 2-2.999 × 109/L, 1-1.999 × 109/L and <1 × 109/L respectively.<br>Grade 3 and grade 4 were severe leucopenia.	Incidence; discontinuation	*Follow-up:*<br>Median 17 months (range 1-144)<br><br>*Timing of tests*:<br>NR	WBC count	Leucopenia (n=81, 8.6%)<br><br>No leucopenia (n=854, 91.4%)
Broekman 2017 (TOPIC trial)<br><br>N=695	Leukopenia	*Moderate or severe Leucopenia:*<br>Moderate (grade 2): WBC count between ≥2.0 x 109/L and <3.0 x 109/L;<br>Severe (grade 3): WBC count <2.0 x 109/L	Incidence; discontinuation; reduction; recovery without intervention	*Follow-up:*<br>20 weeks<br><br>*Timing:* Baseline, week 1, 2, 4, 6, 8, 20	WBC count	Occurrence: n=45 (6.5%); comprising moderate n=41 (5.9%); severe n=4 (0.6%)<br><br>Discontinuation: n=11 (24.4%)<br><br>Reduction: n=20 (44.5%)<br><br>Recovery without intervention: n=12 (26.7%)
Calafat 2019<br><br>ENEIDA registry N=19,630	Myelotoxicity (anaemia; leukopenia; bone marrow suppression; lymphopenia); hepatotoxicity	Not reported	Incidence	*Follow-up:* up to 10 years<br><br>*Timing*: not reported	Not reported	Myelotoxicity: elderly group: n=264 (14.0%); 18-50 group: n=1174 (7.6%)<br><br>Hepatoxicity: elderly group: n=169 (9.0%); 18-50 group: n=721 (4.7%)
Chaparro 2013<br><br>ENEIDA registry N=3931	Leukopenia; bone marrow suppression; pancreatitis; hepatotoxicity	Leukopenia: WBC <3 x 109/L<br>Bone marrow suppression: suppression of the bone marrow activity that leads to anaemia, leucopenia, and thrombocytopenia<br>Pancreatitis: rise in serum amylase levels x 3 above the upper limit	Incidence; Discontinuation	*Follow-up:* Median 44 months (range 0-420)<br><br>*Timing:* not reported	Not reported	Occurrence:<br>Leukopenia: n=162 (4.1%)<br>Bone marrow suppression: n=88 (2.2)<br>Hepatotoxicity: n=156 (4%)<br>Pancreatitis: n=150 (4%)

		Hepatitis: rise in alanine transaminase >2 times the upper limit				
Cheng 2022	Elevated liver enzyme (ELE)	An AST or alkaline phosphatase value above the laboratory's ULN or an<br>ALT value above 33 for males and 25 for females per the American College of Gastroenterology guidelines.	Incidence	*Follow-up:*<br>Median: 49.9 (IQR: 19.9–93.5) months<br><br>*Timing of tests*:<br>NR	Liver function tests	ELE at baseline, n = 52/322 (16.2%)<br><br>ELE during follow-up, n = 114/322 (35.4%)
Coenen 2015 (TOPIC trial)	Haematologic ADRs	Haematologic ADRs: ≤ 3.0 x 109/L	Incidence	*Follow-up:* 20 weeks<br><br>*Timings:* 1 week prior to start of study, then weeks 1, 2, 4, 6, 8 and 20	Blood sampling for TPMT genotyping and enzyme measurements - Leukocytes, thrombocytes, haemoglobin, liver enzymes (ALT/AST)	Haematologic ADRs:<br>Intervention group (pre-treatment genotyping) – 29 (7.2%)<br><br>Control – 29 (7.2%)
Fangbin 2012<br><br>N=199	Leukopenia; hepatotoxicity	Leukopenia: WBC count <3.5 x 109/L<br><br>Hepatotoxicity: Aspartate aminotransferase or alanine aminotransferase level >2 times upper level of normal	Discontinuation	*Follow-up:* 48 weeks<br><br>*Timing:*<br>Full blood count: weekly for 4 weeks then once per month<br><br>Liver function tests: once per month then every 3 months until they were measured 3 times	WBC count;<br>Neutrophil count;<br>Aspartate aminotransferase or alanine aminotransferase level;<br>Serum amylase level	Leukopenia: n=36 (18.09%)<br><br>Hepatotoxicity: n=1 (0.50%)
Houwen 2021	Hepatotoxicity<br><br>Myelotoxicity (includes leukopenia and thrombocyto-penia)	Hepatotoxicity: abnormal liver function test [≥3 times the upper limit of normal ranges for either alanine aminotransferase (ALT) and/or aspartate aminotransferase (AST)<br><br>Myelotoxicity: platelet count < 150 000 platelets/µl and/or leukocyte counts < 4000/µl	Incidence	Follow-up, mean (SD), years:<br>Allopurinol: 3.6 (5.5)<br><br>No allopurinol: 3.5 (4.1)<br><br>*Timing of tests*:<br>NR	NR	No allopurinol vs allopurinol<br><br>N events / Incidence rate<br>Hepatotoxicity:<br>1863 / 149 vs 32 / 65<br>Myelosuppression:<br>27 856 / 3957 vs 801 / 4108<br>Leukopenia:<br>10 001 / 971 vs 314 / 842<br>Thrombocytopenia:<br>4726 / 404 vs 186 / 446
Kakuta 2018<br><br>N=1291	Leukopenia; Liver dysfunction; Pancreatitis	Leukopenia: WBC count <3000/µL	Discontinuation or modification	*Study duration*: 2 years	Not reported	Leukopenia: n=236 (18.3%)<br><br>Liver dysfunction: n=47 (3.6%)<br><br>Pancreatitis: n=20 (1.5%)

Kim 2010<br><br>N=286	Leukopenia	Leukopenia: WBC count <3000/mm	Discontinuation or reduction	*Follow-up: 12 months*<br><br>*Timing:* 1-2 week intervals for the first month, then 1 month later, then every 3 months	Complete blood counts and liver function tests	Leukopenia: n=116 (40.6%) (AZA 110/278; 6-MP 6/8)
Kim 2017<br><br>N=331<br><br>N=267	Leukopenia	Leukopenia: WBC count ≤3000/mm	Discontinuation or reduction	*Study duration*: 14 years	WBC count	Leukopenia: n=113/267 (42.3%)
Kreijne 2020<br><br>N=1132	Myelotoxicity; hepatotoxicity	Myelotoxicity: leukopenia and/or thrombocytopenia. Leukopenia – mild (3.0-4.0 x 109/L), moderate (2.0-3.0 x 109/L), severe <2.0 x 109/L). Thrombocytopenia – mild (100-150 x 109/L), moderate (50-100 x 109/L), severe (<50 x 109/L)<br><br>Hepatotoxicity: increase in AP, GGT, ALT or AST – grade 1 between the ULN and 2.5 x ULN, grade 2 between 2.5 and 5.0 x ULN, grade 3 between 5.0 and 20.0 x ULN.	Discontinuation, dose reduction or additional therapy with allopurinol + reduced thiopurine dose	*Median follow-up* (cessation or censoring): 3.3 years (IQR 1.7-5.6)<br><br>Baseline = 1 year after initial thiopurine treatment	Laboratory toxicity as described for myelotoxicity and hepatotoxicity	Myelotoxicity: 370/1132 (33%)<br><br>Hepatotoxicity: 275/1132 (24%)
Labidi 2020<br><br>N = 210	Myelotoxicity; hepatotoxicity	Myelotoxicity: anaemia (haemoglobin level <10 g/dL); and/or neutropenia (absolute neutrophil count <1,500/mm3 and/or lymphopenia – absolute lymphocyte count <1,500/mm3) and/or thrombocytophenia (platelet count <100,000). Severe myelotoxicity: absolute neutrophil count <500/mm3 and/or lymphocyte count <500/mm3 and/or haemoglobin level <5g/dL and/or platelet count <75,000/mm3.<br><br>Hepatotoxicity: increase in alanine transaminase and/or aspartate transaminase and/or alkaline phosphatase and/or	Incidence	*Median follow-up*: 28.5 +/- 20 months<br><br>*Timing:* Baseline, 1, 2, 4, 12 weeks after commencement of treatment then quarterly.	Full blood count and liver function tests.	Myelotoxicity:<br>25/210 (11.9%)<br><br>Hepatotoxicity:<br>8/210 (3.8%)

		gamma glutamate transpeptidase > 2 x ULN or clinical jaundice. Severe hepatotoxicity: liver function test elevation > 5 x ULN.				
Ribaldone 2019<br><br>N = 200	Leukopenia; pancreatitis; hepatitis	Leukopenia: WBC count < 3.0 x 109/L<br><br>Toxic hepatitis: Elevated ALT or AST levels ≥2 x ULN<br><br>Pancreatitis: clinical symptoms plus serum amylase elevation 3 x ULN	Discontinuation	Not reported	Peripheral venous blood sample for genotyping	*Leukopenia*: 6/200 (10%)<br>*Hepatitis*: 24/200 (40%)<br>*Pancreatitis*: 28/200 (47%)
Wong 2017<br><br>N = 270	Hepatotoxicity	Hepatotoxicity: Elevation from baseline of ALT at least 2 x ULN or by a ratio of ALT and ALP ≥ 5	Incidence	*Follow-up:* 20 weeks<br><br>*Timing:*<br>1 week before initiation, and 1,2,4,6,8 and 20 weeks after initiation	Blood samples for 6-MMPR and 6-TGN collected 1 week after thiopurine initiation	*Hepatotoxicity*: 47/270 (17%)
Park 2016<br><br>N = 964	Leukopenia	Leukopenia: WBC count < 3000/mm3	Early (before 8 weeks) and late (after 8 weeks) incidence	*Study period*: 23 years<br><br>*Timing:* Every two weeks for the first 8 weeks, then monthly for the following 1-2 months, then every 2-3 months	Blood leukocyte levels, genomic DNA	Early leukopenia: 66/964<br>Late leukopenia: 264/964
**SLE, RA, IBD**						
Dickson 2022	Myelotoxicity	Discontinuation of azathioprine attributed to myelotoxicity in EHRs. Five ascribed reasons for myelotoxicity: leukopenia, neutropenia, thrombo-cytopenia, pancytopenia, and anemia. We attributed all patients who had multiple reasons cited for discontinuation, which included myelotoxicity as achieving the outcome, even if the chart cited additional reasons	Discontinuation	*Follow-up:*<br>Median: 19.1 (3.1–61.7) months<br><br>*Timing of tests:*<br>NR	NR	AZA-discontinuation due to myelotoxicity:<br>n=70/1385 (5.1%)
5-ASA=5-acetyl salicylate; AKI=Acute kidney injury; ALT=alanine transaminase; AST=aspartate transaminase; ALP=alkaline phosphatase; Anti-TNF: Anti-Tumour necrosis factor; AS=Ankylosing spondylitis; AZA=Azathioprine; CD=Crohn's disease; DMARD=Disease modifying antirheumatic drugs; eGFR= estimated glomerular filtration rate; FBC=Full blood count; GGT=Gamma glutamyl						

transferase; IBD=Inflammatory Bowel Disease (IBD); IQR=Interquartile range; LFT=Liver function test; MMF=Mycophenolate mofetil; MP=mercaptopurine; MTX=Methotrexate; SLE=Systemic lupus erythematosus; RA=Rheumatoid arthritis; N=number; PsO=Psoriasis; PsA=Psoriatic arthritis; RCT =Randomized controlled trial; SD=Standard deviation; SLE=Systemic lupus erythematosus; Rheumatoid arthritis (RA); SZS=Sulfasalazine; UC=Ulcerative colitis; UEC=Urea and electrolyte test; WBC=White blood cells

Table S2b: Outcome characteristics and frequency of events for included studies – Anti-TNFs:

Study	Outcome	Definition of outcome	Incidence, adjustment or discontinuation	Timing of tests	Details of tests	Number (%) with outcome
**Rheumatoid arthritis; Ankylosing spondylitis; Psoriasis/psoriatic arthritis (mixed); IBD**						
Chiu 2017<br><br>N = 407 (n=368 included in case control analysis)	Liver enzyme elevation	Liver enzyme elevation: serum ALT > 2 x ULN within 1 year of starting treatment	Incidence	*Follow-up:* 12 months<br><br>*Timing:* Baseline then 6 months after start of treatment and then every three months	Serum ALT, HBV serostatus	Liver enzyme elevation: 30/368 (8.2%)
Chiu 2018	Liver enzyme elevation	Liver enzyme elevation: serum ALT > 2 x ULN	Incidence	*At six months, then 3 monthly*	ALT	Anti-TNF 50<br>cDMARD 2.3
Hastings 2010<br><br>N = 367	Neutropenia	Neutropenia: neutrophil count <2 x 109/L	Incidence	*Follow-up:* 12 months<br><br>*Timing:* Baseline, 2 weeks after starting TNF inhibitors, then every month	Neutrophil count, lymphocytes, monocytes, basophils, eosinophils	At least one episode of neutropenia: 69/367 (18.8%)
Madani, 2022<br><br>n=250	Abnormal laboratory results (timepoints)	The time points at which patients developed significantly abnormal laboratory results during treatment with one of the TNF-alpha inhibitors.	Incidence	*Follow-up:*<br>24 months<br><br>*Timing of tests:*<br>Every 3 months	Complete blood count (CBC) with differential counts, liver function tests (LFTs), total cholesterol level, low-density lipoprotein (LDL) level, triglyceride (TG) level, and renal function	Incidence for each test at each timepoint: 'The majority of the significantly abnormal laboratory results were found at baseline and 3-6 and 9-12 months'.
**IBD**						
AlAskar 2020<br><br>n=281	Neutropenia	Absolute neutrophil count (ANC) of <1500/mm3. It was classified into mild, moderate, and severe based on an ANC of 1000–1500, 500–1000, and <500/mm3, respectively	Incidence	*Follow-up:*<br>Up to 11 years<br><br>*Timing of tests*:<br>NR	Neutrophil count<br>WBC count	Neutropenia<br>(n=96, 34.2%)<br><br>No neutropenia<br>(n=185, 65.8%)
Shelton 2015<br><br>N = 1753	Liver enzyme elevation	Liver enzyme elevation: at least one elevated ALT ≥ x 2 ULN	Incidence	*Follow-up:* Mean duration of follow-up of controls – 167 weeks<br><br>Mean time to first ALT elevation – 29 weeks	Alanine transaminase levels	Elevated ALT: 102/1753 (6%)

Study	Outcome	Definition of outcome	Incidence, adjustment or discontinuation	Timing of tests	Details of tests	Number (%) with outcome
				Timing: Prior to Anti-TNF initiation, then every 3-4 months		
Parisi 2016<br><br>N = 305 (n=176 infliximab, n=129 no Anti-TNF)	Liver enzyme elevation	Liver enzyme elevation: mild - ALT ≥ 2ULN; moderate - ALT =2-3 ULN; severe - ALT ≥ 3 ULN	Incidence	*Follow-up*: 5 years<br><br>*Timing:* Baseline, every 8 weeks for infliximab, every 3-6 months for the remainder of the population	Full blood count and liver biochemistry	Elevated ALT: Infliximab 69/176 (39.2%)<br><br>Whole cohort: 111/305 (36.4%)
**Ankylosing spondylitis**						
Choi 2020<br><br>N = 363	Liver enzyme elevation	Liver enzyme elevation: serum AST and/or ALT > ULN and consecutively elevated for at least 2 visits	Incidence	*Study period:* 14 years<br><br>*Median duration of TNFi exposure before elevated AST/ALT*: 3.72 months	Laboratory data: ALT, AST, alkaline phosphatase, bilirubin, antinuclear antibody, human leukocyte antigen-B27 erythrocyte sedimentation rate, C-reactive protein	Elevated liver enzymes: 86/363 (23.7%)
Swart 2022<br><br>N=211	Decline in renal function	Estimated glomerular filtration rate (eGFR) using the MDRD formula in ml/min/1.73m^2: 32,788 x (serum creatinine in µmol/l$^{-1.154}$) x (age in years$^{-0.203}$) x 0.742 if female and/or 1.212 if ethnic black	Level of renal decline over time	*Study period:* 9 years<br><br>*Treatment period*: 2 years<br><br>*Median follow-up time:* 156 (36-286) weeks	Laboratory data	N/R
**Psoriatic arthritis**						
Kavanaugh 2007<br><br>N = 200	Liver enzyme elevation	Liver enzyme elevation: AST >150 IU/I and ≥100% increase from baseline for ALT and AST	Incidence	*Follow-up*: 1 year<br><br>*Timing:*<br>Baseline, week 2, 6, and every 8 weeks until week 54	Laboratory tests for ALT/AST	At one year: combined infliximab group 8/191 (4.2%) abnormal ALT; 4/191 (2.1%) abnormal AST.

5-ASA=5-acetyl salicylate; AKI=Acute kidney injury; ALT=alanine transaminase; AST=aspartate transaminase; ALP=alkaline phosphatase; Anti-TNF: Anti-Tumour necrosis factor; AS=Ankylosing spondylitis; AZA=Azathioprine; CD=Crohn's disease; DMARD=Disease modifying antirheumatic drugs; eGFR= estimated glomerular filtration rate; FBC=Full blood count; GGT=Gamma glutamyl transferase; IBD=Inflammatory Bowel Disease (IBD); IQR=Interquartile range; LFT=Liver function test; MMF=Mycophenolate mofetil; MP=mercaptopurine; MTX=Methotrexate; SLE=Systemic lupus erythematosus; RA=Rheumatoid arthritis; N=number; PsO=Psoriasis; PsA=Psoriatic arthritis; RCT =Randomized controlled trial; SD=Standard deviation; SLE=Systemic lupus erythematosus; Rheumatoid arthritis (RA); SZS=Sulfasalazine; UC=Ulcerative colitis; UEC=Urea and electrolyte test; ULN=Upper Limit of Normal; WBC=White blood cells

Table S2c: Outcome characteristics and frequency of events for included studies – DMARDs:

Study (Drug(s)	Outcome	Definition of outcome	Incidence, adjustment or discontinuation	Timing of tests	Details of tests	Number (%) with outcome
**Rheumatoid arthritis**						
Amital 2009 (Methotrexate)<br><br>N = 809	Hepatotoxicity	Hepatotoxicity: normal values for liver function tests were - serum y-glutamyltransferase (GGT) (10–49 U/l); alkaline phosphatase (ALKP) (34–104 U/l); AST (10–37 U/l); and albumin (3.5–5.2 g/dl)	Incidence	Follow-up:<br>Mean follow-up RA – 843 days, PsO – 883 days	Laboratory liver function tests	Abnormal liver function tests:<br><br>ALKP: PsO – 7.27%, RA – 7.70<br>GGT: PsO – 15.21%, 15.70%<br>Albumin: PsO – 1.83%, RA – 2.45%<br>AST: PsO – 9.88%, RA – 7.17%
Bologna 1996 (Methotrexate)<br><br>N = 469	Elevation of transaminases<br><br>Haematological (macrocytosis, leucopenia, thrombopenia)	*Elevation of transaminases:* N/R<br><br>*Haematological:* N/R	Incidence	*Duration of study:* patients treated between 1985 and 1994 were evaluated in November 1994.<br><br>*Timing:* N/R	N/R	*Transaminase elevation:*<br><65 years: 50 (12.5%)<br>≥65 years: 6 (11.8%)<br><br>*Haematological:*<br><65 years: 15 (3.7%)<br>≥65 years: 4 (7.8%)
Curtis 2010 (Methotrexate and/or Leflunomide)<br><br>N = 2104	Hepatotoxicity	*Hepatotoxicity:* Elevation of ALT/AST > ULN	Incidence	*Timing:* Mean time between study visits where blood was drawn approx. 5 months	Laboratory liver function tests	*Transaminase elevations:*<br><br>RA:<br>MTX + LEF: 31% >1 ULN, 5% >2 ULN<br>MTX: 22% >1 ULN, 1% >2 ULN<br>LEF: 17% >1 ULN, 2% >2 ULN<br>No MTX or LEF: 14% >1 ULN, 2% >2 ULN<br><br>PsA:<br>MTX + LEF: - >1 ULN, - >2 ULN<br>MTX: 35% >1 ULN, 0% >2 ULN<br>LEF: - >1 ULN, - >2 ULN<br>No MTX or LEF: 28% >1 ULN, 3% >2 ULN
Dirven 2013 (Methotrexate)<br><br>N = 508	Elevated liver enzymes	*Liver enzyme elevation:*<br><br>Alanine transferase (ALT) 2 x ULN	Incidence (N= measurements)	*Follow-up:*<br>5 years<br><br>*Mean duration of MTX use*: 2.8 years<br><br>*Timing:*<br>Every 3 months	Laboratory tests	*Elevated liver enzymes:*<br><br>ALT 2 x ULN: 140/5592 measurements in 89/498 patients
Hayashi 2020 (Methotrexate)	Change in eGFR during the previous 1 year	The eGFR was calculated by the equation defined by the Japanese Society of Nephrology:	Incidence	*Follow-up:*	White blood cell (WBC) count, hemoglobin (Hb),	The eGFR (mean ± SD) decreased by 1.2 ± 8.1 mL/min/1.73 m2 from 76.1 ± 17.3 mL/min/1.73 m2

Study (Drug(s)	Outcome	Definition of outcome	Incidence, adjustment or discontinuation	Timing of tests	Details of tests	Number (%) with outcome
		eGFR (mL/min/1.73 m2) = 194 × (serum creatinine [mg/dl])−1.094 × (age)−0.287 × 0.739 (if female)		1 year observation period (data collected from the most recent 1 year period of exposure).<br><br>*Timing of tests*:<br>NR	platelet count, serum creatinine<br>(s-Cr) levels, proteinuria (> 0.5 g/gCr and/or ≥ 2 + on dipstick urinalysis), erythrocyte sedimentation rate (ESR),<br>C-reactive protein (CRP) level	
Hoekstra 2018 (Methotrexate)<br><br>N = 411	Severe hepatotoxicity	Severe hepatotoxicity: Alanine aminotransferase (ALT) ≥ 3 x ULN	Incidence, discontinuation	*Follow-up:*<br>48 weeks	Laboratory tests	*Severe hepatotoxicity:* 36/137 (26%) placebo group, 11/274 (4%) folate group<br><br>*Discontinuation:* 126/411 of which due to toxicity 52/137 placebo, 39/274 folate
Humphreys 2017 (Methotrexate)<br><br>N = 11,839	Transaminitis	*Transaminitis:*<br>Alanine transaminase (ALT) or aspartate aminotransferase (AST) levels 3 x ULN or higher	Incidence	*Follow-up:* study period was 1987 to 2016, unless patient was censored earlier<br><br>*Timings:* 6 times every twelve months	Laboratory tests	*Transaminitis:*<br><br>530/11,839 (4.48%)
Jiang 2012 (Methotrexate)<br><br>N = 505	Hepatic adverse events	*Elevated alanine aminotransferase:*<br>Level of alanine aminotransferase over the normal scope	Incidence	*Follow-up:* treatment was for 24 weeks<br><br>*Timings:*<br>Baseline, then every 4 weeks	Blood chemistry	*Hepatic abnormalities – (events/cases):*<br>WM: 54/22<br>CM: 35/14<br><br>*Alanine aminotransferase elevation (events):*<br>WM: 54<br>CM: 34
N = 213	Elevated liver enzymes	*Elevated liver enzymes:*<br><br>Alanine aminotransferase (ALT) > ULN<br>(predictors are for > 1.5 x ULN)		*Follow-up:* Mean 225 weeks (SD 129)<br><br>*Timings:* every 14 days during first 3 months of treatment with MTX, then every 3 months	Laboratory ALT tests	*Elevated liver enzymes:*<br><br>ALT > ULN: 84/213 (39%)<br>ALT > 1.5 x ULN: 44/213 (21%)<br>ALT > 2 x ULN: 32/213 (15%)<br>ALT > 3 x ULN: 13/213 (6)<br><br>*Discontinuation:*<br>7/213 (3%)
Karlsson Sundbaum 2019, 2021<br><br>*Cavalli 2022	Liver enzyme (alanine amino-transferase [ALT]) elevation (within the first 6 months)	ALT elevation: ALT > 1.5 times the ULN (>44 U/I 0.75 µkat/l) in adult females and >66 U/I 1.1 µkat/l in adult males)	Incidence (at least one elevation of ALT > 1.5 x ULN), discontinuation	*Follow-up:*<br>Minimum 6 months of treatment<br><br>*Timing of tests:*	Laboratory tests according to Swedish guidelines, e.g. ALT and liver function tests	*Elevated liver enzymes:*<br>18 (9%) patients met the criteria for at least one elevation of Elevated

**Study (Drug(s)**	**Outcome**	**Definition of outcome**	**Incidence, adjustment or discontinuation**	**Timing of tests**	**Details of tests**	**Number (%) with outcome**
(Methotrexate)		*Within the first 6 months of treatment		Every 14 days for 3 months, followed by monthly testing for 3 months, and every 3 months thereafter		*ALT (n=33, 9%) vs no elevated ALT (n=313, 91%)ALT
Kent 2004 (Methotrexate)<br><br>N = 481	Hepatic and haematologic abnormalities	*Hepatic abnormalities*: AST levels > ULN, usually 31 U/L<br><br>*Abnormal leukocyte* count: < 3.5 x 109/L<br><br>*Abnormal platelet count*: < 150 x 109/L	Incidence, discontinuation	*Follow-up:*<br>Mean months monitored 58 (SD 38)<br><br>*Timings:* Mean measurements per year 8.4 (SD 3)	Laboratory tests	AST abnormalities: 304/481 (63%) of which: 251/304 1-2 x ULN; 27/304 2-3 x ULN; 9/304 3-4 x ULN; 4/304 4-5 x ULN; 1/304 5-6 x ULN; 2/304 6-7 x ULN<br><br>Leukopaenia:<br>51/481 (11%)<br><br>Thrombocytopaenia:<br>9/481 (1.8%)<br><br>Discontinuation: 22/481 permanent discontinuation, 67/481
Mori 2020 (Methotrexate)<br><br>N = 289	Persistent transaminitis	*Persistent transaminitis:*<br>Elevated ALT and/or AST > ULN (30 IU/L) in 5 out of 9 determinations within a 12 month interval	Incidence	*Follow-up:*<br>Mean follow-up 58.3 months (95% CI: 54.2, 62.5)<br><br>*Timing*: every 4-8 weeks during MTX treatment	Serum ALT and AST levels	Persistent transaminitis:<br><br>44/289 (15.2%)
Nakafero 2022b (Leflunomide)	Drug discontinua-tion with abnormal blood test results	A prescription gap of 90 days, with abnormal blood test results (or diagnostic code indicating an abnormal blood test result) within 660 days of the last prescription	Discontinuation	*Follow-up:*<br>180 days after the first LEF prescription issued by the GP until the earliest of date of outcome, death, transfer out of the practice, date of last data collection from the practice, 5 years or 31 December 2019<br><br>1487 patients with 3140 person-years follow-up<br><br>*Timing of tests*: NR	NR	NR (136 outcome events occurred during the follow-up period at a rate of 43.32/1000 person-years (95% CI 36.62, 51.25)
Sakthiswary 2014 (Methotrexate)	Methotrexate induced NAFLD with transaminitis	Imaging evidence of steatosis and elevated transaminase level	Incidence	*Follow-up:* Duration unclear.	Abnormal LFTs that prompt US imaging	*Transaminitis: 112 patients (11.4%). MTX-associated NAFLD + transaminitis: 4.7% (46 patients).*
Sherbini 2021 (Methotrexate)	Elevated liver enzymes	Elevated ALT >1 x ULN	Incidence	*Follow-up:*<br>6 and 12 months	Laboratory values for ALT	*Elevated ALT enzyme:*<br>*>1 x ULN 0-6 months 120 (11.2%); 6-12 months 119 (11.1%); 0-12 months 192 (18%)*

Study (Drug(s)	Outcome	Definition of outcome	Incidence, adjustment or discontinuation	Timing of tests	Details of tests	Number (%) with outcome
						>2 x ULN 0-6 months 19 (1.8%); 17 (1.6%); 0-12 months 33 (3.1%)
Suzuki 2021 (Methotrexate)	Hepatotoxicity	N/R	Incidence	*Follow-up:*<br>6 and 12 months	N/R	*Hepatic function abnormal:*<br>24 week follow-up:<br>MTX ≥1 year – 61 (4.75%)<br>MTX < 1 year – 117 (11.87%)<br><br>52 week follow-up:<br>MTX ≥1 year – 8 (5.93%)<br>MTX < 1 year – 24 (17.14%)<br><br>*Liver disorder:*<br>24 week follow-up:<br>MTX ≥1 year – 19 (1.48%)<br>MTX < 1 year – 32 (3.23%)<br><br>52 week follow-up:<br>MTX ≥1 year – 2 (1.48%)<br>MTX < 1 year – 7 (5%)
Svanstrom 2018 (Methotrexate)<br><br>N = 17,200	Liver toxicity, acute renal failure, cytopenia	Defined using ICD-10 codes	Incidence (events)	*Follow-up:* 1 year (median 125 days IQR 63-226)	Not reported	*Liver toxicity:*<br>No concomitant NSAIDs – 7 events<br>Concomitant NSAIDS – 3 events<br>*Acute renal failure:*<br>No comcomitant NSAIDs – 22 events<br>Concomitant NSAIDs – 27 events<br>*Cytopenia:*<br>No concomitant NSAIDs – 106 events<br>Concomitant NSAIDs – 86 events
Verstappen 2010 (Methotrexate)<br><br>N = 289	Liver toxicity; Renal toxicity; haematological toxicity	*Liver toxicity*: AST or ALT > ULN at follow-up.	Incidence	*Follow-up:* 2 years<br><br>*Timing:* C-group once every 3 months, I group once every 4 weeks	Laboratory tests for AST, ALT, ESR, Hb, creatinine, creatinine clearance	*Liver toxicity:* Total 82/289 (55%). I group 70/149, C group 40/140<br>*Renal toxicity*: 58/289 (38.9%)<br>*Haematological toxicity*: 38/289 (25.5%)
Yang 2022 (Methotrexate)<br>N = 21,452	Leucopenia	*NR*	Incidence	*Eligibility criteria:* At least 365 days of observation prior to MTX	N/R	*Leucopenia:* 85/21,452 (0.4%)
**Psoriasis**						
Zhu, 2022 (Methotrexate)<br>n=1,440<br><br>MTX=309	Hepatotoxicity<br><br>-ALT elevation;<br>-Abnormal hepatic function	Abnormal liver function was considered if there was an elevation above the normal upper limit of any one of the above four enzyme levels: alanine	Incidence	*Follow-up:*<br>At least 1 year<br><br>*Timing of tests:*<br>Monthly	Liver function tests in the present study included measurement	Across genotypes:<br><br>ALT elevation: 17.11%-37.5%<br><br>Abnormal liver function: 17.05%-37.5%

Study (Drug(s)	Outcome	Definition of outcome	Incidence, adjustment or discontinuation	Timing of tests	Details of tests	Number (%) with outcome
Controls = 1,031		transaminase (ALT), aspartate transaminase (AST), direct bilirubin, and total bilirubin			of ALT, AST, direct bilirubin (DBIL), and total bilirubin (TBIL) levels.	
**Rheumatoid arthritis; Ankylosing spondylitis; Psoriasis/psoriatic arthritis (mixed)**						
Gelfand 2021 (Methotrexate)<br><br>N = 40,237	Liver disease	*Mild liver disease*: chronic hepatitis or cirrhosis without portal hypertension<br>*Moderate to severe liver disease*: liver failure, hepatic encephalopathy, portal hypertension, esophageal varices; Cirrhosis; cirrhosis-related hospitalisation	Incidence	*Study period:* 18 years	Not reported	Liver disease events:<br>Mild liver disease:<br>PsO: 169<br>PsA: 102<br>RA: 329<br><br>Moderate to severe liver disease:<br>PsO: 40<br>PsA: 22<br>RA: 108<br><br>Cirrhosis:<br>PsO: 77<br>PsA: 36<br>RA: 100<br><br>Hospitalisations due to cirrhosis:<br>PsO: 30<br>PsA: 14<br>RA: 53
Schmajuk 2014 (Methotrexate)<br><br>N = 659	Elevated transaminases	Mild abnormalities: AST/ALT between ULN and 1.5 x ULN<br><br>Moderate abnormalities: AST/ALT ≥ 1.5 x ULN<br><br>Severe abnormalities: > 10 x ULN	Incidence	*Follow-up:*<br>Mean follow-up 227 days (SD 147)	Liver function tests	Elevated transaminases:<br><br>Mild or moderate – 126/659 (19%<br>Severe - 0
Tilling 2006 (Methotrexate)<br><br>N = 619	Hepatic toxicity	Hepatic toxicity:<br>Abnormal transaminase level (AST and ALT) 3 x ULN on 2 or more occasions	Incidence, discontinuation	*Timing:* Baseline, then weekly until treatment stabilised, then every 2-3 months	Full blood count, renal profile, crythrocyte sedimentation rate, C-reactive protein and liver function tests	*Elevation of transaminase levels:*<br><br>RA – 41 (7.5%)<br>PsA – 10 (14.5%)<br><br>*Discontinuation:*<br>RA – 7 (1.2%)<br>PsA – 4 (5.8%)
Kwon 2018 (Methotrexate)	Cytopenia	*Cytopenia:*<br>Haemoglobin level decreased by ≥2 g/dl or platelet count of <	Incidence	*Study duration:* 2005-2017<br><br>*Timing:* N/R	Laboratory tests	Cytopenia:<br>9/175 (5.1%) of which 3/9 anaemia, 4/9 leukopenia, 2/9 thrombocytopenia

Study (Drug(s)	Outcome	Definition of outcome	Incidence, adjustment or discontinuation	Timing of tests	Details of tests	Number (%) with outcome
N = 175		150,000/mm^3 or WBC of < 3500/mm^3				
**SLE, RA, Ps, IBD, AS**						
Nakafero 2022a (Mycophenolate) n=992	Drug discontinuation associated with abnormal or severely abnormal blood-test results	Defined as a prescription gap of 90 days, with an abnormal or severely abnormal blood-test result or SNOMED code indicating such a result within 660 days of the last prescription date	Incidence, adjustment, discontinuation	*Follow-up:* Cohort exit was assigned as the earliest of date of the following outcomes: death, transfer out of the GP practice, last data collection from the GP practice, 5-year follow-up, or 31 December 2019<br><br>992 patients with 1885 person-years of follow-up<br><br>*Timing of tests*: NR	White blood cells (WBCs); neutrophils; platelets; ALT/ AST; kidney function; creatinine	Drug discontinuations due to:<br>**Abnormal monitoring**<br>Ever: 118 (11.9%)<br>First 12 months 86 (8.7%)<br>After 12 months: 32 (3.2%)<br><br>**Severe abnormal monitoring**<br>Ever: 20 (2%)<br>First 12 months 12 (1.2%)<br>After 12 months: 8 (0.8%)
5-ASA=5-acetyl salicylate; AKI=Acute kidney injury; ALT=alanine transaminase; AST=aspartate transaminase; ALP=alkaline phosphatase; Anti-TNF: Anti-Tumour necrosis factor; AS=Ankylosing spondylitis; AZA=Azathioprine; CD=Crohn's disease; DMARD=Disease modifying antirheumatic drugs; eGFR= estimated glomerular filtration rate; FBC=Full blood count; GGT=Gamma glutamyl transferase; IBD=Inflammatory Bowel Disease (IBD); IQR=Interquartile range; LFT=Liver function test; MMF=Mycophenolate mofetil; MP=mercaptopurine; MTX=Methotrexate; SLE=Systemic lupus erythematosus; RA=Rheumatoid arthritis; N=number; PsO=Psoriasis; PsA=Psoriatic arthritis; RCT =Randomized controlled trial; SD=Standard deviation; SLE=Systemic lupus erythematosus; Rheumatoid arthritis (RA); SZS=Sulfasalazine; UC=Ulcerative colitis; UEC=Urea and electrolyte test; ULN=Upper Limit of Normal; WBC=White blood cells						

Table S2d: Outcome characteristics and frequency of events for included studies – various drugs

Study	Outcome	Definition of outcome	Incidence, adjustment or discontinuation	Timing of tests	Details of tests	Number (%) with outcome
**IBD**						
Koller 2017<br><br>N = 251	Liver injury	Liver injury: Grade 1 – ALT 1-3 x ULN; grade 2 – ALT >3 x ULN; hepatocellular injury in ALT > 2 x ULN; cholestatic injury in simultaneous GGT and ALP elevation > ULN.	Incidence – n=measurements	*Follow-up*: 1 year<br><br>*Timings:* Baseline, then every 3 months until 1 year. Aminotransferase recorded from records for 3 months prior to study entry.	Blood sampling for serum activities of ALT, AST, GGT, ALP and serum bilirubin concentration.	*Liver injury:*<br>Grade 1 ALT – 112/917 (12.2%)<br>Grade 1 AST – 55/917 (6%)<br>ALT 2 x ULN – 26/917 (2.84%)<br>Grade 2 ALT – 6/917 (0.65%)<br>Grade 2 AST – 8/917 (0.87%)
**Psoriatic Arthritis**						
Pakchotanon 2020<br><br>N = 1061	Liver abnormalities	Liver biochemical abnormalities: elevated transaminases ≥ 1.5 x ULN	Incidence and prevalence	*Timings*: 6 and 12 month intervals	Laboratory tests for liver enzymes	*Liver abnormalities*:<br><br>Prevalence 343/1061 (32%)<br>Incidence 39/1000 patient years
**Rheumatoid arthritis**						

Fragoulis 2018<br><br>N = 771	Neutropaenia	*Neutropenia:*<br>Grade 1 - < 2.0 x 109/L; Grade 2 - <1.5 x 109/L; Grade 3 - <1.0 x 109/L; Grade 4 - <0.5 x 109/L	Incidence, discontinuation	*Follow-up:* median 18 months (range 6-48 months)<br><br>*Timings:*<br>Baseline then every 6 months	Laboratory tests: full blood count, biochemistry profile, acute phase reactants, rheumatoid factor, anti-Cyclic Citrullinated Peptide	*Neutropaenia:*<br><br>All drugs - 58/771 (7.5%), at a median 12 months (range 0-120 months) after diagnosis<br><br>Grade 1 n=42, grade 2 n=14, grade 3 n=1, grade 4 n=1<br><br>Methotrexate: 52/58 (89.6%)<br>Sulfasalasine: 33/58 (56.9%)<br>Hydroxychloroquine: 31/58 (53.4%<br>Leflunomide: 3/58 (5.2%)<br>Azathioprine: 1/58 (1.7%)<br>Prednisone: 4/58 (7.0%)<br>Corticosteroids: 48/58 (82.8%)<br>Anti-TNF: 5/58 (8.6%)
**RA and IBD**						
Fraser, 2022	Lack of blood test abnormality	Within specified NICE thresholds and tests (test thresholds: WBC ≥3.5×10 9/L; MCV ≤105 fL; neutrophils ≥1.6×10 9/L; platelets ≥140×10 9/L; eosinophils ≤0.5×10 9/L; ALT ≤100 U/L; AST ≤100 U/L; albumin ≥30 g/L; and eGFR ≥60 ml/min/1.73 m2).	Incidence	*Follow-up:*<br>2 years (study duration)<br><br>Timing of tests: 3 months	ALT, WBC, and eGFR	No abnormalities of any NICE-specified blood tests over 2 years<br><br>RA/methotrexate: 1585/3001 (52.8%)<br><br>IBD/azathioprine: 657/1162 (56.5%)
5-ASA=5-acetyl salicylate; AKI=Acute kidney injury; ALT=alanine transaminase; AST=aspartate transaminase; ALP=alkaline phosphatase; Anti-TNF: Anti-Tumour necrosis factor; AS=Ankylosing spondylitis; AZA=Azathioprine; CD=Crohn's disease; DMARD=Disease modifying antirheumatic drugs; eGFR= estimated glomerular filtration rate; FBC=Full blood count; GGT=Gamma glutamyl transferase; IBD=Inflammatory Bowel Disease (IBD); IQR=Interquartile range; LFT=Liver function test; MMF=Mycophenolate mofetil; MP=mercaptopurine; MTX=Methotrexate; SLE=Systemic lupus erythematosus; RA=Rheumatoid arthritis; N=number; PsO=Psoriasis; PsA=Psoriatic arthritis; RCT =Randomized controlled trial; SD=Standard deviation; SLE=Systemic lupus erythematosus; Rheumatoid arthritis (RA); SZS=Sulfasalazine; UC=Ulcerative colitis; UEC=Urea and electrolyte test; ULN=Upper Limit of Normal; WBC=White blood cells						

Table S3: Summary of QUIPs ratings for included studies by drug class

Author, Year	Study participation	Study Attrition	Prognostic Factor measurement	Outcome measurement	Study Confounding	Statistical analysis/reporting	DRUG	DISEASE
**Methotrexate**								
Amital, 2009[34]	L	H	L	L	H	L	MTX	PsO +/- PsA, RA
Bologna 1996[35]							MTX	RA
Cavalli 2022[36]	M	H	L	L	H	L	MTX	RA
Curtis 2010[37]	L	L	L	L	L	L	MTX (mainly), LEF	RA
Dirven, 2013[38]	L	L	L	L	L	M	MTX	RA
Gelfand, 2021[40]	L	M	L	L	M	L	MTX	PsO +/- PsA,
Hayashi 2020[41]	L	M	M	L	L	M	MTX	RA
Hoekstra, 2003[42]	L	M	L	L	M	L	MTX	RA
Humphreys, 2017[43]	L	M	L	L	M	M	MTX	RA
Jiang, 2012[44]	L	H	M	L	M	M	MTX +/- SSZ	RA
Kent, 2004[46]	M	M	M	L	M	L	MTX	RA
Kwon, 2018[47]	M	M	L	L	M	M	MTX	AIRD
Mori, 2020[48]	L	M	L	L	L	M	MTX	RA
Sakthiswary, 2014[52]	L	M	L	L	M	H	MTX	RA
Schmajuk, 2014[53]	M	M	L	L	L	L	MTX	AIRD (RA, AS, PsA)
Sherbini 2021[54]	L	M	L	L	L	L	MTX	RA
Suzuki 2021[49]	L	H	L	L	H	M	MTX	RA
Svanstrom, 2018[56]	L	M	L	L	L	L	MTX	RA
Tilling, 2006[57]	M	M	L	L	H	H	MTX	RA, PsA
Verstappen, 2010[58]	L	M	L	L	M	M	MTX	RA
Yang 2022[60]	L	H	L	L	L	M	MTX	RA
Zhu 2022[59]	L	H	L	L	M	M	MTX	PsO
**5-Acetyl salicylate**								
Achit 2022[5]	L	H	M	L	H	H	5-ASA	IBD
Cheng 2022[10]	L	M	M	L	M	M	5-ASA	IBD
**Mycophenolate mofetil**								
Nakafero 2022a[51]	L	M	L	L	L	L	MMF	SLE, RA, PsO, IBD, AS
**Leflunomide**								
Nakafero 2022b[50]	L	M	L	L	L	L	LEF	RA
**Anti-TNFs**								
AlAskar 2020[23]	L	M	L	L	M	L	ADA, CTZ, IFX, GOL	IBD
Chiu, 2017[24]	H	M	M	L	M	L	ADA, ETA, GOL	AIRD (RA, AS, PsA)

Chiu, 2018[25]	L	M	M	L	M	M	ADA, ETA, GOL	AIRD (RA, AS, PsA)
Choi, 2020[26]	L	H	M	L	L	M	ADA, IFX, ETA, GOL	AS
Hastings, 2010[27]							ADA, ETA, IFX	AIRD (RA, AS, PsA)
Kavanaugh, 2007[28]	L	M	L	L	H	H	IFX	PsA
Madani 2022[29]	L	M	H	L	H	H	ADA, ETA, IFX	RA, AS, PsO, IBD
Parisi, 2016[30]	L	L	L	L	M	M	IFX	IBD
Shelton, 2015[31]	L	M	L	L	H	H	ADA, CTZ, IFX,	IBD
Swart 2022[32]	L	H	L	L	M	M	ADA, ETA	AS
**Thiopurines**								
Banerjee 2020[6]	L	M	L	L	L	L	AZA	IBD
Broekman, 2017[7]	L	L	L	L	M	L	Thiopurine	IBD
Calafat, 2019[8]	L	M	M	M	M	L	Thiopurine	IBD
Chaparro, 2013[9]	L	M	L	L	M	L	Thiopurine	IBD
Coenen, 2015[11]	L	L	L	L	L	L	Thiopurine	IBD
Dickson 2022[12]	L	M	M	L	M	M	AZA	SLE, RA, IBD
Fangbin, 2012[13]	L	M	L	L	H	H	Thiopurine	IBD
Houwen 2021[14]	H	M	L	L	M	L	Thiopurine	IBD
Kakuta, 2018[15]	M	M	L	L	M	M	Thiopurine	IBD
Kim, 2010[16]	M	M	L	L	H	M	Thiopurine	IBD
Kim, 2017[17]	L	M	L	L	M	L	Thiopurine	IBD
Kreijne, 2015[18]	L	M	M	L	M	L	Thiopurine	IBD
Labidi, 2020[19]	L	M	L	L	M	M	Thiopurine	IBD
Park 2016[20]	L	L	L	L	M	M	Thiopurine	CD
Ribaldone, 2019[21]	H	M	L	L	H	H	AZA	IBD
Wong, 2017[22]	M	M	L	L	M	L	Thiopurine	IBD
**Cohorts prescribed different classes of drugs**								
Fragoulis, 2018[39]	M	M	L	L	M	L	Multiple	RA
Fraser 2022[61]	L	H	M	L	M	M	MTX, AZA	RA (MTX), IBD (AZA)
Koller, 2017[62]	L	M	L	L	L	L	Thiopurine +/- 5-ASA +/- ANTI-TNF	IBD
Pakchotanon, 2020[63]	L	M	L	L	L	L	ANTI-TNF and/or DMARDs	PsA

L: low; M: moderate; H: High; 5-ASA: 5-acetyl salicylate; ADA: adalimumab or biosimilar; AIRD: autoimmune inflammatory rheumatic disease; AS: ankylosing spondylitis; AZA:azathioprine; CD: Crohn's disease; CTZ: certolizumab or biosimilar; ETA: etanercept or biosimilar; GOL: golimumab or biosimilar; IBD: inflammatory bowel disease; IFX: infliximab or biosimilar; LEF: leflunomide; MTX: methotrexate; MMF: mycophenolate mofetil; PsA: psoriatic arthritis; PsO: Psoriasis; RA: rheumatoid arthritis; SSZ: sulfasalazine.

Supplementary results: differences in outcomes.

Conventional DMARDs

The most commonly studied outcome in the 25 studies evaluating DMARDs was hepatoxicity, recorded in 15 studies (although terminology varied widely between studies). This outcome was variously defined as hepatotoxicity[20,23,27,33,43], elevation of transaminases[21,22,37], elevated liver enzymes[24,29,38], transaminitis[19,36], hepatic adverse events[28], hepatic abnormalities[30], persistent transaminitis[32], liver/hepatic toxicity[40-42], abnormal hepatic function[43] and liver disease.[18] Severity of hepatotoxicity was sometimes graded e.g. into ‘mild’, ‘moderate’ and ‘severe’. Most studies reported AST and/or ALT at various levels > ULN to record incidence of the outcome, even where the outcome was not graded by severity.

Six studies recorded haematological adverse events such as leukopenia[21,44], neutropenia[25], cytopenia[31,40], or simply haematological ‘events’[30] or ‘toxicity’.[42] One study reported change in eGFR in the previous year as the dependent variable.[26]

Twenty-six of the 27 studies reported ‘incidence’ of the outcome; six of these studies also recorded whether participants discontinued with the drug due to the recorded adverse events.[25,27,29,30,34,41] Only one study reported as the sole outcome of interest discontinuation due to abnormal blood test results.[34]

Follow-up of participants varied widely, ranging from within the first 6 months[39] to 18 years.[18]

Anti-TNFs

Six of the 10 studies evaluating anti-TNFs reported liver enzyme elevation as the outcome.[46-48,50,52,53] As with the studies evaluating thiopurines, individual studies varied in whether they recorded the outcome by severity of liver enzyme elevation, e.g. mild, moderate and severe, or whether participants were simply recorded as having the outcome if they met the reported threshold, most commonly > 2 x ULN. Two studies recorded neutropenia as the outcome, as defined by neutrophil count.[45,49] Two studies assessed predictors of abnormal laboratory results[51] and decline in renal function.[54] Nine studies reported ‘incidence’ of the outcome; the exception reported scale of decline in renal function.[54] Follow-up of participants ranged from 12 months[46,50] to 14 years.[48]

Thiopurines

The most commonly studied outcome in the 18 studies evaluating thiopurines was leukopenia, with nine studies measuring this outcome.[57,59,63-67,70,71] Leukopenia was sometimes graded by severity e.g. ‘mild’, ‘moderate’ or ‘severe’, and most studies reported the WBC threshold used to record incidence of the outcome, even where the outcome was not graded by severity. In addition to these studies that only recorded leukopenia, a further five studies included leukopenia under the broader outcome of myelotoxicity, along with other haematologic adverse events such as bone marrow suppression, lymphopenia or neutropenia.[58,62,64,68,69] Similarly, one study reported ‘haematologic adverse events’ as the outcome in their study.[61] Nine studies measured hepatotoxicity.[56,58,59,63-65,68,69,72] This was reported to be measured in most studies by either elevation of alanine transaminase and/or aspartate aminotransferase, and this was also sometimes graded by severity but sometimes not. One study reported acute kidney injury (AKI) as the outcome.[55]

Eleven of the studies reported ‘incidence’ of the outcome[55-61,64,69,70,72], three of which also reported discontinuation due to the adverse event.[56,57,59]

The remaining seven studies explicitly recorded the outcome only if it had caused discontinuation or dose adjustment.[62,63,65-68,71] Follow-up of participants varied widely, ranging from 20 weeks (e.g. Broekman et al 2017[57]) to 23 years.[70]

Combinations of classes of drug

Three studies evaluated populations taking a combination of different classes of drugs relevant to the review[73-75] and studied liver injury, graded by severity[74] and liver abnormalities.[73,75] All three studies reported ‘incidence’ of the outcome. The length of follow-up was reported by two studies and ranged from 1 year[74] to 2 years.[73]

Table S4a: Prognostic factors for cytopenia and nephrotoxicity in those prescribed methotrexate

Prognostic factor Author, year	N	Exposure	Referent	RM[1]	Point estimate (95% CI)	Other treatment	Disease
**Cytopenia**							
Age							
Bologna, 1996[2]	469	≥65 years	<65	OR	2.18 (0.70-6.84)		RA[3]
Kwon, 2018	175	Age per year increase		HR	0.96 (0.92-1.00)	cDMARD[4]	RA
Sex							
Kent, 2004	481	Female	Male	aOR	1.90 (1.10-2.70)		RA
Kwon, 2018	175	Female	Male	HR	1.25 (0.26-6.03)	cDMARD	RA
Yang, 2022*	21452	Female	Male	OR	1.41 (0.84-2.49)		RA
Comorbidity							
Kwon, 2018	175	Hypertension	None	HR	1.03 (0.21-4.97)	cDMARD	RA
Yang, 2022*	21452	Chronic obstructive lung disease		OR	1.5 (0.8-2.65)		RA
Yang, 2022*	21452	Congestive heart failure		OR	2.94 (1.45-5.47)		RA
Yang, 2022*	21452	Type 2 diabetes mellitus without complication		OR	1.47 (0.88-2.38)		RA
Yang, 2022*	21452	Hypertension		OR	1.36 (0.87-2.16)		RA
Yang, 2022*	21452	Hyperlipidemia		OR	1.58 (1.01-2.49)		RA
Liver							
Kwon, 2018	175	Chronic liver disease	None	HR	5.83 (1.21-28.06)	cDMARD	RA
Renal							
Kwon, 2018	175	Creatinine level		HR	1.14 (0.02-87.04)	cDMARD	RA
DMARDs							
Kwon, 2018	175	Methotrexate dose		HR	0.92 (0.73-1.15)	cDMARD	RA
Kwon, 2018	175	Leflunomide	None	HR	5.77 (0.72-46.19)	cDMARD	RA
Kwon, 2018	175	Mycophenolate	None	HR	3.02 (0.38-24.24)	cDMARD	RA
Other drugs							
Kwon, 2018	175	NSAIDs	None	HR	0.54 (0.14-2.00)	cDMARD	RA
Kwon, 2018	175	Aspirin	None	HR	2.08 (0.26-16.66)	cDMARD	RA
Kwon, 2018	175	Antibiotics	None	HR	2.43 (0.30-19.45)	cDMARD	RA
Kwon, 2018	175	TMP-SMX[5]	None	HR	1.72 (0.35-8.37)	cDMARD	RA

Svanstrom, 2018	21536	NSAIDs	None	aHR	1.35 (1.01-1.81)		RA
Bone marrow function							
Kwon, 2018	175	Mean corpuscular volume		HR	0.95 (0.82-1.13)	cDMARD	RA
**Nephrotoxicity**							
Other drugs							
Svanstrom, 2018	21536	NSAID	None	aHR	2.04 (1.14-3.66)		RA

[1]RM: Risk measure;[2]Haematological, includes leucopenia, thrombopenia and macrocytosis; [3]RA: Rheumatoid arthritis; [4]cDMARD: conventional disease modifying anti-rheumatic drug; [5]TMP-SMX: trimethoprim-sulfamethoxazole; [6]b/cDMARD: biologic/conventional disease modifying anti-rheumatic drug; * calculated from crude data; Yang 2022 outcome is leucopenia;

Table S4b. Summary of GRADE judgements: Prognostic factors for cytopenia and nephrotoxicity in those prescribed methotrexate

Prognostic factor	Summary of findings	Quality of evidence	Reason for grading up or down
Cytopenia			
Age	No evidence of increased risk with increasing age. Evidence from two studies (Bologna, 1996; Kwon, 2018) with 644 participants.	Very low	Imprecision
Sex	Conflicting evidence for increased risk for either gender. Evidence from three studies (Kent, 2004; Kwon, 2018; Yang, 2022) with 22,108 participants.	Very low	Inconsistency
Comorbidities	No evidence from two studies for increased risk with comorbid hypertension (Kwon, 2018; Yang, 2022) with 21,627 participants. Evidence from one study (Yang, 2022), with 21,452 participants of increased risk of cytopenia with congestive heart failure or hyperlipidemia. No evidence of increased risk with type 2 diabetes from one study with 21,452 participants (Yang, 2022).	Very low	Inconsistency
Liver disease, elevated liver enzymes	Evidence for increased risk (Kwon, 2018) with 175 participants.	Low	Large effect upgrade two levels, downgrade one level each due to imprecision and single study
Renal	No evidence for increased risk (Kwon, 2018) with 175 participants.	Very low	Imprecision, single study
Other immune-suppressing treatments	No evidence of increased risk with MMF and leflunomide Kwon, 2018) with 175 participants.	Very low	Imprecision, single study

Other drugs	No evidence of increased risk with co-prescription of aspirin, antibiotics, but increased risk with co-prescription of NSAIDs from two studies (Kwon, 2018; Svanstrom, 2018) with 21711 participants.	Very low	Imprecision
Marrow function	Evidence of reduced risk with increasing baseline neutrophil count. Evidence from one study (Kwon, 2018), including 175 participants.	Very low	Single study
**Nephrotoxicity**			
Other drugs	Evidence of increased risk with co-prescription of NSAIDs from one study (Svanstrom, 2018), including 21536 participants.	Moderate	Large effect upgrade two, single study downgrade one.

Table S5a: Demographic prognostic factors for hepatotoxicity in those prescribed methotrexate

Prognostic factor Author, year	N	Exposure	Referent	RM[1]	Point estimate (95% CI)	Other treatment	Disease
Age							
Amital, 2009	809	Age per year increase		aHR	1.00 (0.99-1.01)		RA[2], PsO[3]
Bologna, 1996	469	≥65 year	<65 year	OR	0.93 (0.38-2.30)		RA
Cavalli, 2022*	346	Age at RA diagnosis		SMD	-0.01 (-0.37-0.34)		RA
Dirven, 2013	498	Age per year increase		OR	0.99 (0.98-1.01)	c/bDMARD[4]	RA
Karlsson, 2019	210	Age per year increase		aOR	1.00 (0.90-1.00)		RA
Mori, 2020	289	Age per year increase		HR	1.00 (0.98-1.02)		RA
Sherbini, 2021	1069	Age per year increase		aOR	0.99 (0.97-1)		RA
Suzuki[b], 2021	2860	≥ 65 years	<65 years	aOR	0.63 (0.42-0.96)*		RA
Sakthiswary, 2014	138	Age per year increase		SMD	0.13 (-0.48-0.23)		RA
Verstappen, 2010	289	Age per year increase		aOR	1.01 (0.98-1.03)	conventional[5] +/- ciclosporin	RA
Verstappen, 2010	289	Age per year increase		aOR	1.02 (0.99-1.04)	Intensive[5] +/- ciclosporin	RA
Zhu[a], 2022	146	Age per year increase		aOR	0.98 (0.96-0.99)		PsO
Zhu[b], 2022	146	Age per year increase		aOR	0.98 (0.95-0.99)		PsO
Sex							
Cavalli, 2022	346	Female	Male	OR	4.36 (1.3-22.78)		RA
Gelfand, 2021	40237	Male	Female	aHR[6]	1.05 (0.88-1.24)		PsO +/- PsA[10]
Gelfand, 2021	40237	Male	Female	aHR[7]	1.37 (1.00-1.88)		
Gelfand, 2021	40237	Male	Female	aHR[8]	1.36 (0.89-2.06)		
Gelfand, 2021	40237	Male	Female	aHR[9]	0.93 (0.70-1.24)		
Dirven, 2013	498	Female	Male	OR	1.21 (0.73-1.99)	c/bDMARD	RA
Karlsson, 2019	210	Female	Male	aOR	5.70 (1.70-19.70)		RA
Mori, 2020	289	Male	Female	HR	0.68 (0.32-1.44)		RA
Sakthiswary, 2014	138	Female	Male	OR	1.14 (0.44-2.08)		RA

Sherbini, 2021	1069	Female	Male	aOR	0.79 (0.55-1.15)		RA
Verstappen, 2010	289	Female	Male	aOR	0.60 (0.28-1.29)	conventional +/- ciclosporin	RA
Verstappen, 2010	289	Female	Male	aOR	1.16 (0.58-2.35)	intensive +/- ciclosporin	RA
Tilling, 2006	619	Male	Female	OR	0.88 (0.49-1.57)		RA, PsA
Amital, 2009	809	Female	Male	aHR	1.46 (1.16-1.84)		RA, PsO
Body mass index (BMI) (kg/m^2)							
Cavalli, 2022*	346	BMI per kg/m^2 increase		SMD	0.21 (-0.15-0.57)		RA
Curtis, 2010	2104	25-30 kg/m^2	<25	aOR	2.97 (0.77-11.41)	Leflunomide	PsA
Curtis, 2010	2104	>30 kg/m^2	<25	aOR	3.09 (0.84 11.45)	Leflunomide	PsA
Curtis, 2010	2104	25-30 kg/m^2	<25	aOR	1.21 (0.90-1.61)	Leflunomide	RA
Curtis, 2010	2104	>30 kg/m^2	<25	aOR	1.22 (0.92-1.62)	Leflunomide	RA
Mori, 2020	289	>25 kg/m^2	=<25	aHR	2.68 (1.37-5.25)		RA
Schmajuk, 2014	659	≥30 kg/m^2	<30	aOR	1.90 (1.00-3.60)		RA, AS[11], PsA
Hoekstra, 2018	411	High	Low	aOR	1.09 (1.01-1.17)		RA
Karlsson, 2019	210	BMI per kg/m^2 increase		aOR	1.10 (1.00-1.20)		RA
Dirven, 2013	498	BMI per kg/m^2 increase		OR	1.01 (0.95-1.06)	c/bDMARD	RA
Sakthiswary, 2014	138	BMI per kg/m^2 increase		SMD	0.18 (-0.17-0.54)		RA
Sherbini, 2021	1069	BMI per kg/m^2 increase		aOR	1.02 (0.99-1.05)		RA
Suzuki[a], 2021	2860	BMI ≥ 25kg/m^2		aOR	2.08 (1.22-3.54)*		RA
Verstappen, 2010	289	BMI per kg/m^2 increase		aOR	1.12 (1.00-1.25)	conventional +/- ciclosporin	RA
Verstappen, 2010	289	BMI per kg/m^2 increase		aOR	1.08 (0.97-1.21)	intensive +/- ciclosporin	RA
Zhu[a], 2022	146	BMI per kg/m^2 increase		aOR	1.15 (1.05-1.25)		PsO
Zhu[b], 2022	146	BMI per kg/m^2 increase		aOR	1.11 (1.03-1.20)		PsO
Body Surface Area (BSA)							
Zhu, 2021	146	BSA at baseline		aOR	1.02 (1.00-1.03)		PsO
Disease duration							

Dirven, 2013	498	RA duration		OR	0.99 (0.98-1.01)	RA
Mori, 2020	289	RA duration		HR	1.00 (0.98-1.02)	RA
Sakthiswary,2014*	138	RA duration (months)		SMD	0.15 (-0.2-0.51)	RA
Ethnicity						
Sakthiswary, 2014*	138	Chinese	Malay	OR	0.82 (0.32-2.21)	RA
Cavalli, 2022*	346	At least one Swedish parent	Both parents Swedish	OR	1.79 (0.61-4.57)	RA

[1]RM: Risk measure; [2]RA: Rheumatoid Arthritis; [3]PsO: Psoriasis; [4]c/b DMARD: conventional/biologic disease modifying anti-rheumatic drug; conventional/intensive[5]: methotrexate dosing strategy; [6]mild liver disease is the outcome; [7]moderate liver disease is the outcome; [8] hospitalisation for liver disease is the outcome; [9]cirrhosis is the outcome; [10]PsO +/- PsA: psoriasis and/or psoriatic arthritis; [11]AS: ankylosing spondylitis; *rounded to 2 decimal places; Suzuki[a] MTX ≥ 1 year 24 week follow-up period; Suzuki[b] MTX < 1 year 24 week follow-up period; Suzuki[c] MTX ≥ 1 year 52 week follow-up period; Susuki[d] MTX < 1 year 52 week follow-up period; Zhu[a] outcome is ALT elevation; Zhu[b] outcome is abnormal liver function; *calculated from crude data

Table S5b. Summary of GRADE judgements: Demographic prognostic factors for hepatotoxicity in those prescribed methotrexate

Prognostic factor	Summary of findings	Quality of evidence	Reason for grading up or down
Age	No evidence of increased risk with increasing age. Evidence from eleven studies (Amital, 2009; Bologna, 1996; Dirven, 2013; Karlsson, 2019; Mori, 2020, Verstappen 2010; Cavalli, 2022; Sherbini, 2021; Suzuki, 2021; Sakthiwary, 2014; Zhu, 2022) with 7,123 participants.	Low	
Sex	Evidence for increased risk for either sex. Evidence from ten studies (Gelfand, 2021; Dirven, 2013; Karlsson, 2019; Mori, 2020; Verstappen, 2010; Tilling, 2006; Amital, 2009; Cavalli, 2022; Sakthiwary, 2014; Sherbini, 2021) with 84,367 participants.	Very low	Inconsistency
Disease duration	No evidence for increased risk for RA duration from three studies (Dirven, 2013; Mori, 2020; Sakthiswary, 2014), with 925 participants	Low	
Body mass index	Evidence for increased risk in seven studies (Curtis, 2010; Mori, 2020; Schmajuk, 2014; Hoekstra, 2018; Karlsson, 2019; Dirven, 2013; Verstappen, 2010) with 8,730 participants.	Very Low	Inconsistency
Body surface area	Evidence from one study (Zhu, 2021) with 146 participants of increased risk of hepatotoxicity with increasing body surface area.	Very low	Single study
Ethnicity	No evidence from two studies (Sakthiswary, 2014, Cavalli, 2022), with 484 participants that ethnicity is associated with increased risk of hepatotoxicity.	Very low	Imprecision

Table S6a: Lifestyle prognostic factors for hepatotoxicity in those prescribed methotrexate

Prognostic factor Author, year	N	Exposure	Referent	RM[1]	Point estimate (95% CI)	Other treatment	Disease
Alcohol							
Cavalli, 2022*	346	Alcohol glasses/week		SMD	0.11 (-0.47-0.25)		RA
Gelfand, 2021	40237	Abuse	None	aHR[2]	3.62 (2.86-4.59)		PsO +/- PsA[6]
Gelfand, 2021	40237	Abuse	None	aHR[3]	5.49 (3.68-8.19)		
Gelfand, 2021	40237	Abuse	None	aHR[4]	6.50 (3.95-10.72)		
Gelfand, 2021	40237	Abuse	None	aHR[5]	5.94 (4.24-8.31)		
Curtis, 2010	2104	1-3 drinks/week	None/ occasional	aOR	1.22 (0.46-3.24)	Leflunomide	PsA
Curtis, 2010	2104	≥ 1 drink/day	None/ occasional	aOR	2.18 (0.52-9.15)	Leflunomide	PsA
Curtis, 2010	2104	1-3 drinks/week	None/ occasional	aOR	0.97 (0.72-1.31)	Leflunomide	RA[7]
Curtis, 2010	2104	1-2 drinks/day	None/ occasional	aOR	1.97 (1.18-3.28)	Leflunomide	RA
Dirven, 2013	498	Alcohol use	None	OR	1.29 (0.81-2.03)	c/bDMARD[8]	RA
Humpreys, 2017	11839	1-7 units/week	Non-drinker	aHR	1.03 (0.82-1.28)		RA
Humpreys, 2017	11839	8-14 units/week	Non-drinker	aHR	1.01 (0.73-1.40)		RA
Humpreys, 2017	11839	15-21 units/week	Non-drinker	aHR	1.35 (0.85-2.14)		RA
Humpreys, 2017	11839	>21 units/week	Non-drinker	aHR	1.85 (1.17-2.93)		RA
Humpreys, 2017	11839	Alcohol drinker	Non-drinker	aHR	1.06 (0.86-1.30)		RA
Sherbini, 2021	1069	Alcohol drinker	Non-drinker	aOR	1.15 (0.79-1.69)		RA
Caffeine intake							
Sherbini, 2021	1069	Caffeine cups per day	None	aOR	1.03 (0.96-1.1)		RA
Smoking							
Cavalli, 2022*	346	Ever smoker	Never smoked	OR	1.34 (0.61-2.96)		RA
Gelfand, 2021	40237	Smoker	Not current	aHR[2]	0.90 (0.68-1.20)		PsO +/- PsA
Gelfand, 2021	40237	Smoker	Not current	aHR[3]	0.72 (0.42-1.24)		

Gelfand, 2021	40237	Smoker	Not current	aHR[4]	0.58 (0.26-1.28)		
Gelfand, 2021	40237	Smoker	Not current	aHR[5]	0.80 (0.51-1.25)		
Curtis, 2010	2104	Smoker	Not current	aOR	1.35 (0.40-4.55)	Leflunomide	PsA
Curtis, 2010	2104	Smoker	Not current	aOR	0.58 (0.40-0.82)	Leflunomide	RA
Dirven, 2013	498	Smoker	Not current	aOR	1.60 (0.98-2.70)	c/bDMARD	RA
Mori, 2020	289	Smoker	Not current	HR	0.65 (0.33-1.28)		RA
Sherbini, 2021	1069	Former smoker	Never smoked	aOR	0.93 (0.63-1.36)		RA
Sherbini, 2021	1069	Smoker	Never smoked	aOR	1.14 (0.7-1.85)		RA
Zhu[a], 2022	146	Smoker	Not current	aOR	1.15 (1.05-1.25)		PsO
Zhu[b], 2022	146	Smoker	Not current	aOR	1.11 (1.03-1.20)		PsO

[1]RM: Risk measure; [2]mild liver disease is the outcome; [3]moderate liver disease is the outcome; [4]hospitalisation for liver disease is the outcome; [5]cirrhocis is the outcome; [6]PsO +/- PsA: cutaneous psoriasis and/or psoriatic arthritis; [7]RA: Rheumatoid Arthritis; [8]c/b DMARD: conventional/biologic disease modifying anti-rheumatic drug; Zhu[a] outcome is ALT elevation; Zhu[b] outcome is abnormal liver function; *calculated from crude data

Table S6b. Summary of GRADE judgements. Lifestyle prognostic factors for hepatotoxicity in those prescribed methotrexate

Prognostic factor	Summary of findings	Quality of evidence	Reason for grading up or down
Alcohol	Evidence from six studies with 56,093 participants of increased risk with excessive alcohol consumption. Evidence of increased risk from four studies (Gelfand, 2021; Curtis, 2010; Dirven, 2013; Humpreys, 2017); but no evidence of association from two studies (Cavalli, 2022, Sherbini 2021)	Moderate	Large effect size and dose response. Downgrade one for inconsistency.
Smoking	Evidence for no increased risk for smoking. Evidence from seven studies (Gelfand, 2021; Dirven, 2013; Mori, 2020; Curtis, 2010; Cavalli, 2022; Sherbini, 2021; Zhu, 2022) with 44,801 participants.	Low	

Table S7a: Comorbid prognostic factors for hepatotoxicity in those prescribed methotrexate

Author, year	N	Exposure	Referent	RM	Point estimate (95% CI)	Other treatment	Disease
Comorbidity							
Cavalli, 2022*	346	Diabetes		OR	1.26 (0.23-4.55)		RA

Gelfand, 2021	40237	Diabetes	None	aHR[2]	1.89 (1.43-2.50)		
Gelfand, 2021	40237	Diabetes	None	aHR[3]	2.24 (1.40-3.57)		PsO +/- PsA[6]
Gelfand, 2021	40237	Diabetes	None	aHR[4]	1.56 (0.77-3.14)		
Gelfand, 2021	40237	Diabetes	None	aHR[5]	2.50 (1.67-3.72)		
Mori, 2020	289	Diabetes	None	aHR	2.76 (1.02-7.48)		RA[7]
Sakthiswary, 2014	138	Fasting blood sugar		aOR	0.56 (0.27-1.18)	+/- cDMARDs	RA
Schmajuk, 2014	659	Diabetes	None	OR	1.10 (0.60-2.20)		RA, AS, PsA
Sherbini, 2021	1069	Diabetes	None	aOR	1.19 (0.67-2.14)		
Gelfand, 2021	40237	Hyperlipidemia	None	aHR[2]	1.01 (0.78-1.31)		
Gelfand, 2021	40237	Hyperlipidemia	None	aHR[3]	1.01 (0.65-1.58)		PsO +/ PsA
Gelfand, 2021	40237	Hyperlipidemia	None	aHR[4]	0.92 (0.62-1.38)		
Gelfand, 2021	40237	Hyperlipidemia	None	aHR[5]	0.97 (0.51-1.82)		
Kent, 2004	481	Hyperlipidemia	None	aOR	3.40 (1.50-5.30)		RA
Schmajuk, 2014	659	Cholesterol (total) > 240	≤ 240	aOR	5.80 (2.20-15.20)		RA, AS, PsA
Mori, 2020	289	Hypertension	None	HR	1.49 (0.83-2.67)		RA
Gelfand, 2021	40237	CCI[9]		aHR[2]	1.12 (1.01-1.24)		
Gelfand, 2021	40237	CCI		aHR[3]	1.13 (0.97-1.32)		PsO +/- PsA
Gelfand, 2021	40237	CCI		aHR[4]	1.10 (0.87-1.40)		
Gelfand, 2021	40237	CCI		aHR[5]	1.24 (1.09-1.40)		
Schmajuk, 2014	659	CCI> 3	CCI ≤ 3	aOR	1.90 (1.00-3.60)		RA, PsA, AS[10],
Drugs							
Cavalli, 2022	210	Statin	None	aOR	7.80 (1.40-42.60)		RA
Sakthiswary, 2014*	138	Statin		OR	1.68 (0.65-4.24)		RA
Mori, 2020	289	NSAID	None	HR	1.51 (0.84-2.72)		RA
Sherbini, 2021	1069	NSAID	None	aOR	0.94 (0.66-1.33)		
Sherbini, 2021	1069	Oral steroids	None	aOR	1 (0.68-1.47)		
Sherbini, 2021	1069	csDMARDs	None	aOR	0.61 (0.39-0.94)		
Svanstrom, 2018	21536	NSAIDs	None	aHR	0.46 (0.11-1.82)		RA

[1]RM: Risk measure; [2]mild liver disease is the outcome; [3]moderate liver disease is the outcome; [4]hospitalisation for liver disease is the outcome; [5]cirrhocis is the outcome; [6]PsO +/- PsA: cutaneous psoriasis and/or psoriatic arthritis; [7]RA: Rheumatoid Arthritis; [8]cDMARD: conventional disease modifying anti-rheumatic drug;[9]CCI:Charlson comorbidity index; [10]AS: ankylosing spondylitis; *calculated from crude data

Table S7b. Summary of GRADE judgements: Comorbid prognostic factors for hepatotoxicity in those prescribed methotrexate

Prognostic factor	Summary of findings	Quality of evidence	Reason for grading up or down
Diabetes	Evidence of increased risk. Evidence from five studies (Gelfand, 2021; Mori, 2010; Schmajuk, 2013; Cavalli, 2022; Sherbini, 2021) with 42,600 participants.	Moderate	Large effect size up two, down one for inconsistency

Fasting blood sugar	No evidence from one study (Sakthiswary, 2014), with 138 participants of increased risk of hepatotoxicity associated with fasting blood sugar.	Very low	Single study
Hyperlipidaemia	Evidence of increased risk. Evidence from three studies (Gelfand, 2021; Kent, 2004; Schmajuk, 2014) with 41377 participants.	Very low	Inconsistency
Comorbidity composite score	Evidence of increased risk. Evidence from two studies (Gelfand, 2021; Schmajuk, 2014) with 42237 participants.	Low	
NSAIDs	No evidence for increased risk. Evidence from three studies (Mori, 2020; Svanstrom, 2018; Sherbini, 2021) with 22,894 participants.	Very low	Imprecision
Statin	Evidence of increased risk. Evidence from two studies (Cavalli, 2022*; Sakthiswary, 2014) with 348 participants.	Very low	Inconsistency
Hypertension	No evidence from one study (Mori, 2020), with 289 participants, of increased risk with hypertension	Very low	Single study
Oral steroids	No evidence from one study (Sherbini, 2021), with 1069 participants, of increased risk with oral steroids	Very low	Single study
csDMARDs	Evidence from one study (Sherbini, 2021), with 1069 participants, of reduced risk with csDMARDs	Very low	Single study

*From Karlsson Sundbaum 2019

Table S8a: Liver, renal and serologic prognostic factors for hepatotoxicity in those prescribed methotrexate

Prognostic factor Author, year	N	Exposure	Referent	RM[1]	Point estimate (95% CI)	Other treatment	Disease
Liver							
Curtis, 2010	2104	Liver disease	None	aOR	4.96 (0.95-25.90)	Leflunomide	PsA[2]
Curtis, 2010	2104	Liver disease	None	aOR	2.07 (1.38-3.08)	Leflunomide	RA[3]
Dirven, 2013	498	Liver disease	None	OR	0.57 (0.07-4.61)	c/bDMARD[4]	RA
Mori, 2020	289	Mild fat deposition	None	aHR	1.85 (0.94-3.67)		RA
Mori, 2020	289	Mod - Severe fat deposition	None	aHR	7.69 (3.10-19.10)		RA
Suzuki[a], 2021	2860	Hepatic function disorder	None	aOR	2.84 (1.34-6.02)*		RA
Suzuki[b], 2021	2860	Hepatic function disorder	None	aOR	2.45 (1.31-4.60)*		RA
Suzuki[c], 2021	2860	Hepatic function disorder	None	aOR	7.16 (1.32-39.00)*		RA
Suzuki[d], 2021	2860	Hepatic function disorder	None	aOR	6.80 (1.99-23.21)*		RA
Cavalli, 2022*	346	History of ALT elevation prior to MTX		OR	4.36 (1.3-22.78)		
Dirven, 2013	498	ALT[5] > Normal		aOR	3.10 (1.60-6.20)	c/bDMARD	RA
Schmajuk, 2014	659	ALT > Normal	Normal	aOR	3.20 (1.20-8.40)		RA, AS[6], PsA
Sherbini, 2021	1069	ALT, units/litre		aOR	1.04 (1.03-1.05)		RA
Suzuki[b], 2021	2860	ALT level ≥40 IU/L before administration		aOR	2.50 (1.30-4.78)*		RA
Verstappen,2010	289	ALT, units/litre		aOR	1.08 (1.04-1.12)	conventional +/- ciclosporin	RA
Verstappen, 2010	289	ALT, units/litre		aOR	1.04 (0.99-1.09)	conventional +/- ciclosporin	RA
Verstappen, 2010	289	ALT, units/litre		aOR	1.03 (1.00-1.06)	intensive +/- ciclosporin	RA
Suzuki[c], 2021	2860	AST level ≥40IU/L pre-treatment		aOR	10.25 (1.11-94.72)		RA
Verstappen, 2010	289	AST[7], units/litre		aOR	1.01 (0.97-1.06)	intensive +/- ciclosporin	RA
Renal							
Mori, 2020	289	Creatinine-low-CKD	High	HR	0.55 (0.20-1.51)		RA
Sakthiswary, 2014*	138	Creatinine µmol/L		SMD	0.14 (-0.21-0.5)		RA
Verstappen, 2010	289	Creatinine µmol/L		aOR	1.03 (1.00-1.07)	conventional +/- ciclosporin	RA
Verstappen, 2010	289	Creatinine µmol/L		aOR	0.99 (0.97-1.02)	intensive +/- ciclosporin	RA
Serology							
Dirven, 2013	498	Anti-CCP[8] +ve	None	aOR	1.80 (1.10-3.10)	c/bDMARD	RA
Mori, 2020	289	Anti-CCP +ve	None	HR	2.04 (0.50-8.39)		RA
Cavalli, 2022*	346	RF+ve		OR	1.07 (0.48-2.54)		RA
Dirven, 2013	498	RF[9] +ve	None	OR	1.68 (1.00-2.81)	c/bDMARD	RA
Mori, 2020	289	RF +ve	None	HR	0.80 (0.38-1.67)		RA
Cavalli, 2022*	346	ACPA positivity		OR	1.51 (0.65-3.81)		RA
Sherbini, 2021	1069	ACPA positivity	None	aOR	0.85 (0.49-1.47)		RA

Sherbini, 2021	1069	RF positivity	None	aOR	0.88 (0.5-1.52)		RA
Sherbini, 2021	1069	eGFR	None	aOR	0.99 (0.98-1.01)		RA
Sakthiswary, 2014*	138	RF +ve		OR	0.96 (0.44-2.08)		RA
Verstappen, 2010	289	RF +ve	None	aOR	1.04 (0.46-2.34)	conventional +/- ciclosporin	RA
Verstappen, 2010	289	RF positive +ve	None	aOR	1.20 (0.58-2.49)	intensive +/- ciclosporin	RA
Inflammatory markers							
Mori, 2020	289	CRP[10] > 1.5 mg/dl		HR	1.33 (0.73-2.42)		RA
Dirven, 2013	498	ESR[11], mm/hr		OR	1.00 (0.99-1.01)	c/bDMARD	RA
Mori, 2020	289	ESR >28 mm/h		HR	1.96 (1.00-3.89)		RA
Verstappen, 2010	289	ESR, mm/hr		aOR	1.00 (0.99-1.02)	conventional +/- ciclosporin	RA
Genotype							
Zhu, 2021	163	T allele	C allele	aOR	1.96 (1.19-3.21)		PsA
Zhu, 2021	163	CT	CC	aOR	2.49 (1.19-5.35)		PsA
Zhu, 2021	163	TT	CC	aOR	3.31 (1.25-5.37)		PsA
Zhu, 2021	163	CT or TT	CC	aOR	2.67 (1.31-5.37)		PsA
Zhu, 2021	163	TT	CC or CT	aOR	1.99 (0.78-4.63)		PsA
Zhu, 2021	146	T allele	C allele	aOR	0.7 (0.38-1.28)		PsO
Zhu, 2021	146	CT	CC	aOR	0.49 (0.19-1.28)		PsO
Zhu, 2021	146	TT	CC	aOR	0.56 (0.19-1.81)		PsO
Zhu, 2021	146	CT or TT	CC	aOR	0.51 (0.21-1.2)		PsO
Zhu, 2021	146	TT	CC or CT	aOR	0.85 (0.33-2.31)		PsO
Zhu, 2021	146	rs1801130		aOR	0.46 (0.22-0.96)		PsO

[1]RE: Risk estimate; [2]PsA: psoriatic arthritis; [3]RA: rheumatoid arthritis; [4]c/bDMARD: conventional or biologic disease modifying anti-rheumatic drug; [5]ALT: alanine transaminase; [6]AS: ankylosing spondylitis; [7]AST: aspartate transaminase; [8]Anti-CCP: anti-CCP antibody; [9]RF: rheumatoid factor; irrhocis is the outcome; [10]CRP: C-reactive protein; [11]ESR: erythrocyte sedimentation rate; *rounded down to 2 decimal places; Suzuki[a] MTX ≥ 1 year 24 week follow-up period; Suzuki[b] MTX < 1 year 24 week follow-up period; Suzuki[c] MTX ≥ 1 year 52 week follow-up period; Susuki[d] MTX < 1 year 52 week follow-up period; *calculated from crude data.

Table S8b: Liver, renal and serologic prognostic factors for hepatotoxicity in those prescribed methotrexate

Prognostic factor	Summary of findings	Quality of evidence	Reason for grading up or down
Elevated liver enzymes	Evidence of increased risk. Evidence from six studies (Cavalli, 2022; Dirven, 2013; Schmajuk, 2014; Verstappen, 2010; Sherbini, 2021; Suzuki, 2021) with 5,931 participants.	High	Large effect size, upgrade two
Liver disease	Evidence from four studies (Curtis, 2010; Dirven, 2013; Mori, 2020; Suzuki, 2021) with 5,751 participants that liver disease is associated with increased risk of hepatotoxicity.	High	Increase for dose response and large effect size, remain high even though down one for inconsistency.
High creatinine	Evidence of increased risk. Evidence from four studies (Mori, 2020; Verstappen, 2010; Sakthiswary, 2014; Sherbini 2021) with 1785 participants.	Very low	Inconsistent

Serology	No evidence of increased risk. Evidence from six studies (Mori, 2020; Verstappen, 2010; Dirven 2013; Cavalli, 2022; Sherbini, 2021; Sathiswary, 2014) with 2,629 participants.	Low	
Inflammatory markers	No evidence of increased risk from three studies (Mori, 2020; Verstappen, 2010; Dirven 2013) with 1,076 participants.	Low	
Genotype	Evidence from one study (Zhu, 2021), with 146 participants, that specific genotypes are associated with increased risk of hepatotoxicity.	Very low	Single study and within study inconsistency

Table S9a: Disease severity and activity related prognostic factors for hepatotoxicity in those prescribed methotrexate

Prognostic factor<br>Author, year	N	Exposure	Referent	RM[1]	Point estimate (95% CI)	Other treatment	Disease
Disease severity and activity							
Dirven, 2013	498	Disease activity score		OR	0.89 (0.68-1.17)	c/bDMARD[2]	RA[3]
Sakthiswary, 2014*	138	Disease activity score (DAS 28)		SMD	0.32 (-0.04-0.68)		RA
Dirven, 2013	498	HAQ-DI[4]		OR	0.74 (0.52-1.04)	c/bDMARD	RA
Verstappen, 2010	289	HAQ-DI		aOR	1.21 (0.67-2.20)	conventional +/- ciclosporin	RA
Verstappen, 2010	289	HAQ-DI		aOR	0.88 (0.51-1.15)	intensive +/- ciclosporin	RA
Mori, 2020	289	Steinbrocker stages III/IV	I/II	HR	1.39 (0.72-2.68)		RA
Verstappen, 2010	289	Swollen joint count		aOR	1.03 (0.96-1.09)	conventional +/- ciclosporin	RA
Verstappen, 2010	289	Swollen joint count		aOR	1.04 (0.99-1.10)	intensive +/- ciclosporin	RA
Verstappen, 2010	289	Tender joint count		aOR	1.00 (0.95-1.06)	conventional +/- ciclosporin	RA
Verstappen, 2010	289	Tender joint count		aOR	1.02 (0.97-1.07)	intensive +/- ciclosporin	RA
Verstappen, 2010	289	Visual analogue scale (VAS) well-being, mm		aOR	1.01 (0.99-1.03)	conventional +/- ciclosporin	RA
Verstappen, 2010	289	VAS well-being, mm		aOR	1.00 (0.99-1.02)	intensive +/- ciclosporin	RA
Verstappen, 2010	289	VAS pain, mm		aOR	1.00 (0.98-1.01)	conventional +/- ciclosporin	RA
Verstappen, 2010	289	VAS pain, mm		aOR	1.00 (0.98-1.01)	intensive +/- ciclosporin	RA
Inflammatory disease type							
Gelfand, 2021	40237	PsA[5]	RA	aHR[6]	1.27 (1.01-1.60)		
Gelfand, 2021	40237	PsA	RA	aHR[7]	0.93 (0.58-1.50)		PsO[10] +/- PsA
Gelfand, 2021	40237	PsA	RA	aHR[8]	1.17 (0.64-2.16)		
Gelfand, 2021	40237	PsA	RA	aHR[9]	1.63 (1.10-2.42)		
Tilling, 2006	619	PsA	RA	OR	2.38 (1.17-5.26)		RA, PsA
Gelfand, 2021	40237	PsO	RA	aHR[6]	2.22 (1.81-2.72)		

Gelfand, 2021	40237	PsO	RA	aHR[7]	1.56 (1.05-2.31)	
Gelfand, 2021	40237	PsO	RA	aHR[8]	2.25 (1.37-3.69)	
Gelfand, 2021	40237	PsO	RA	aHR[9]	3.38 (2.44-4.68)	PsO +/- PsA
Schmajuk, 2014	659	PsO	RA, AS[11]	OR	1.20 (0.60-2.40)	RA, AS, PsA
Amital, 2009	809	RA	PsO	aHR	0.85 (0.62-1.17)	RA, PsO

[1]RE: risk estimate; [2]c/bDMARD: conventional or biologic disease modifying anti-rheumatic drug; [3]RA: rheumatoid arthritis; [4]HAQ-DI: health assessment questionnaire (disability index); [5]PsA: Psoriatic arthritis; [6]aHR: mild liver disease is the outcome; [7]aHR: moderate liver disease is the outcome; [8]aHR: liver disease requiring hospitalisation is the outcome; [9]aHR: cirrhosis is the outcome; [10]PsO: cutaneous psoriasis; [11]AS: ankylosing spondylitis.; *calculated from crude data

Table S9b. Summary of GRADE judgments: Disease severity and activity related prognostic factors for hepatotoxicity in those prescribed methotrexate

Prognostic factor	Summary of findings	Quality of evidence	Reason for grading up or down
Disease severity	No evidence of increased risk. Evidence from four studies (Mori, 2020; Verstappen, 2010; Dirven 2013; Sakthiswary, 2014) with 1,214 participants.	Low	
Disease type	Evidence of increased risk with psoriasis compared to RA. Evidence from four studies (Gelfand, 2021; Tilling, 2006; Schmajuk, 2014; Amital, 2009) with 42,324 participants. One of the studies (Amital 2009) found no evidence of association for RA compared to PsO.	Moderate	Large effect size, upgrade two. Inconsistency downgrade one.

Table S10a: Treatment related prognostic factors for hepatotoxicity or nephrotoxicity in those prescribed methotrexate

Prognostic factor Author, year	N	Exposure	Referent	RM[1]	Point estimate (95% CI)	Other treatment	Disease
**Hepatotoxicity**							
Anti-TNF[2]							
			Strategy				
Dirven, 2013	498	Infliximab combo	Mono	OR	1.10 (0.59-2.06)	c/bDMARD[3]	RA[4]
Dirven, 2013	498	Time on infliximab		OR	0.72 (0.54-0.95)	c/bDMARD	RA
Curtis, 2010	2104	Anti-TNF	None	aOR	0.82 (0.31-2.17)	Leflunomide	PsA
Curtis, 2010	2104	Anti-TNF	None	aOR	1.15 (0.90 -1.47)	Leflunomide	RA
Mori, 2020	289	Anti-TNF	None	HR	0.38 (0.05-2.86)		RA
Schmajuk, 2014	659	Anti-TNF	None	aOR	2.40 (0.90-6.60)		RA, AS[5], PsA
Other DMARDs							
Dirven, 2013	498	Cyclosporin (time on)		OR	1.08 (0.63-1.86)	c/bDMARD	RA
Dirven, 2013	498	Hydroxychloroquine (time on)		OR	0.59 (0.26-1.35)	c/bDMARD	RA
Curtis, 2010	2104	Leflumonide	None	aOR	0.83 (0.63-1.77)	Leflunomide	RA
Dirven, 2013	498	No. of concurrent DMARDs		OR	0.71 (0.57-0.90)	c/bDMARD	RA
Sakthiswary, 2014*	138	Prednisolone	None	OR	1.15 (0.52-2.5)		RA
Sakthiswary, 2014*	138	Prednisolone dose (mg/day)		SMD	0.35 (0.002-0.71)		RA
Sherbini, 2021	1069	Oral steroids	None	aOR	1 (0.68-1.47)		
Mori, 2020	289	Prednisolone	None	aHR	1.96 (1.09-3.51)		RA
Dirven, 2013	498	Prednisolone (time on)		OR	0.49 (0.28-0.84)	c/bDMARD	RA
Dirven, 2013	498	Prednisone as combo	Strategy mono	OR	0.58 (0.29-1.17)	c/bDMARD	RA
Dirven, 2013	498	Sulfasalazine (time on)		OR	0.70 (0.52-0.94)	c/bDMARD	RA
Sakthiswary, 2014*	138	Sulfasalazine		OR	1.55 (0.68-3.46)		RA
Sakthiswary, 2014*	138	Sulfasalazine dose (g/day)		SMD	0.27 (-0.09-0.62)		RA
Sakthiswary, 2014*	138	Hydroxychloroquine		OR	0.87 (0.38-1.93)		RA

Sakthiswary, 2014*	138	Hydroxychloroquine dose (mg/day)		SMD	0.21 (-0.15-0.56)		RA
			Strategy				
Dirven, 2013	498	Step up therapy	mono	OR	1.27 (0.68-2.38)	c/bDMARD	RA
Curtis, 2010	2104	MTX[6] (2.5 – 7.5 mg/wk) + LEF[7].	None	aOR	2.15 (0.79-5.85)		RA
Curtis, 2010	2104	MTX (10 – 17.5 mg/wk) + LEF.	None	aOR	2.91 (1.23-6.90)		RA
Curtis, 2010	2104	MTX (≥ 20 mg/wk) + LEF.	None	aOR	3.98 (1.72-9.24)		RA
Sakthiswary, 2014*	138	Leflunomide		OR	0.84 (0.33-2.03)		RA
Sakthiswary, 2014*	138	Leflunomide dose (mg/day)		SMD	1.68 (0.65-4.24)		RA
Other Drugs							-
Hoekstra, 2018	411	Folate Yes	No	aOR	0.10 (0.04-0.21)		RA
Kent, 2004	481	Folate No	Yes	aOR	3.90 (1.80-6.00)		RA
Schmajuk, 2014	659	Folate No	Yes	aOR	2.20 (1.20-4.20)		RA,AS, PsA[8]
Methotrexate dose							
Amital, 2009	809	Cumulative dose		aHR	1.07 (1.02-1.12)		RA, PsO[9]
Cavalli, 2022*	346	MTX maximum weekly dose		SMD	-0.02 (-0.38-0.34)		RA
Sakthiswary, 2014	138	Cumulative dose		aOR	1.04 (0.09-1.05)	cDMARDs	RA
Mori, 2020	289	Dose per 1.0mg /wk		HR	1.11 (0.96-1.29)		RA
Sakthiswary, 2014	138	Current dose		aOR	0.94 (0.84-1.06)	cDMARDs	RA
Sakthiswary, 2014*	138	Duration on MTX		SMD	0.11 (-0.24-0.47)		RA
Dirven, 2013	498	Mean dose		OR	1.08 (1.02-1.13)	c/bDMARD	RA
Curtis, 2010	2104	2.5-7.5 mg/wk	No MTX	aOR	1.23 (0.25-5.90)		PsA
Curtis, 2010	2104	10 – 17.5 mg/wk	No MTX	aOR	3.04 (0.70-13.29)		PsA
Curtis, 2010	2104	≥ 20 mg/wk	No MTX	aOR	2.37 (0.81-6.92)		PsA
Curtis, 2010	2104	2.5-7.5 mg/wk	No MTX	aOR	1.33 (0.88-1.99)		RA
Curtis, 2010	2104	10 – 17.5 mg/wk	No MTX	aOR	1.11 (0.76-1.62)		RA
Curtis, 2010	2104	≥ 20 mg/wk	No MTX	aOR	1.41 (0.96-2.08)		RA
Schmajuk, 2014	659	>15 mg/wk	< 15 mg/wk	OR	1.50 (0.80-2.80)		RA,AS, PsA

**Nephrotoxicity**						
MTX dose						
Hayashi, 2022	502	MTX < 8mg/week	8 ≤ MTX < 12mg/week	aβ	0.86 (-0.65-2.36)	RA
Hayashi, 2022	502	MTX ≥ 12mg/week	8 ≤ MTX < 12mg/week	aβ	-2.46 (-4.3-0.62)	RA

[1]RM: risk measure;[2]TNF: tumour necrosis factor; [3]c/bDMARD: conventional or biologic disease modifying anti-rheumatic drug; [4]RA: rheumatoid arthritis; [5]AS: ankylosing spondylitis; [6]MTX: methotrexate; [7]LEF: leflunomide; [8]PsA: Psoriatic arthritis; [9]PsO: cutaneous psoriasis; *reviewer calculated from crude data

Table S10b. Summary of GRADE judgements: Treatment related prognostic factors for hepatotoxicity or nephrotoxicity in those prescribed methotrexate

Prognostic factor	Summary of findings	Quality of evidence	Reason for grading up or down
Folate supplementation	Evidence of reduced risk. Evidence from three studies (Hoekstra, 2018; Kent, 2004; Schmajuk, 2014) with 1,551 participants.	High	Large effect size, upgrade two
Leflunomide	Evidence of increased risk with leflunomide in combination with Methotrexate. Evidence from two studies (Curtis, 2010; Sakthiswary 2014) with 2,740 participants.	Moderate	Leflunomide plus one for dose-response and large effect size each, minus one for inconsistency.
Anti-TNF	No evidence of increased risk with anti-TNF combined with methotrexate. Evidence from four studies (Dirven, 2013; Curtis, 2010; Mori, 2020; Schmajuk, 2014) 3550 patients.	Low	
HCQ/SASP	Evidence for no association between hydroxychloroquine or sulfasalazine co-prescription from two studies (Dirven, 2013; Sakthiswary, 2014) with 636 patients.	Very low	Imprecision, inconsistency
Oral corticosteroid	No evidence of association between prednisolone co-prescription from four studies with 1994 patients (Dirven, 2013; Sakthiswary, 2014; Mori 2020; Sherbini, 2021).	Very low	Imprecision and inconsistency
Methotrexate dose	No evidence of increased risk with methotrexate dose. Evidence from seven studies (Schmajuk 2014, Dirven 2013, Amital 2009, Mori 2020, Sakthiswary 2014, Curtis 2010; Cavalli, 2022) with 4,843 participants.	Low	
**Nephrotoxicity**			
Methotrexate dose	No evidence of increased risk of nephrotoxicity with methotrexate dose. Evidence from one study (Hayashi, 2022), with 502 participants.	Very low	Single study

Table S11a: Prognostic factors for hepatotoxicity and renal function in those prescribed anti-TNF alpha

Prognostic factor Author, year	N	Exposure	Referent	RM[1]	Point Estimate (95% CI)	Other treatment	Disease
**Hepatotoxicity**							
Age							
Shelton, 2015*	96	Age per year increase		SMD	0.09 (-0.31-0.49)		IBD
Chiu, 2017	368	Age per 20-year increase		aOR	0.47 (0.24-0.91)	cDMARD[2]	RA[3], PsA[4]
Sex							
Choi, 2020	363	Male	Female	aHR	4.62 (1.43-15.01)		AS[5]
Chiu, 2017	368	Female	Male	aOR	0.91 (0.34-2.54)	cDMARD	RA, PsA
Shelton, 2015*	96	Female	Male	OR	1.71 (0.69-4.3)	cDMARD	IBD[6]
Body mass index (BMI)							
Choi, 2020	363	BMI per kg/m^2 increase		HR	1.03 (1.01-1.06)		AS
Pakchotanon, 2020	1061	BMI per kg/m^2 increase		aOR	1.07 (1.02-1.12)	cDMARD	PsA
Lifestyle							
Choi, 2020	363	Hazardous alcohol	None	HR	1.23 (0.49-3.12)		AS
Pakchotanon, 2020	1061	Daily alcohol	None	aOR	4.46 (1.30-15.28)	cDMARD	PsA
Choi, 2020	363	Smoker	None	HR	1.34 (0.87-2.08)		AS
Comorbidity							
Choi, 2020	363	Diabetes	None	HR	2.40 (1.11-5.20)		AS
Pakchotanon, 2020	1061	Diabetes	None	OR	1.64 (0.85-3.19)	cDMARD	PsA
Choi, 2020	363	Hyperlipidemia	None	aHR	2.53 (1.38-4.64)		AS
Pakchotanon, 2020	1061	Hyperlipidemia	None	OR	2.50 (1.45-4.32)	cDMARD	PsA
Choi, 2020	363	Hypertension	None	HR	1.26 (0.76-2.10)		AS
Pakchotanon, 2020	1061	Hypertension	None	OR	3.33 (2.05-5.43)	cDMARD	PsA
Liver							
Choi, 2020	363	NAFLD[7]	None	aHR	4.06 (2.11-7.84)		AS

Choi, 2020	363	Bilirubin level		HR	2.10 (0.86-5.15)		AS
Choi, 2020	363	ALT[8] (IU/L)		aHR	1.14 (1.09-1.19)		AS
Chiu, 2017	368	ALT elevated	None	aOR	13.71 (4.32-45.75)	cDMARD	RA, PsA
Parisi, 2016	305	ALT elevated	None	OR	3.85 (1.80-8.25)	5-ASA, AZA	
Renal							
Choi, 2020	363	Creatine level		HR	3.25 (0.98-10.75)		AS
Serology							
Choi, 2020	363	Positive ANA[9]	None	HR	0.63 (0.20-2.07)		AS
Pakchotanon, 2020	1061	Positive RF[10]	None	OR	2.50 (0.49-12.89)	cDMARD	PsA
Pakchotanon, 2020	1061	Positive ANA	None	OR	1.18 (0.67-2.09)	cDMARD	PsA
Disease type							
Shelton, 2015*	96	UC	CD	OR	0.91 (0.35-2.33)	cDMARD	IBD
Disease duration, disease activity							
Pakchotanon, 2020	1061	PsA, duration		OR	1.08 (1.05-1.11)	cDMARD	PsA
Pakchotanon, 2020	1061	PsO[11], duration		OR	1.02 (1.01-1.04)	cDMARD	PsA
Pakchotanon, 2020	1061	Psoriasis activity score		OR	1.01 (0.98-1.04)	cDMARD	PsA
Pakchotanon, 2020	1061	Damaged joint count		aOR	1.04 (1.01-1.08)	cDMARD	PsA
Pakchotanon, 2020	1061	Swollen joint count		OR	1.03 (0.99-1.06)	cDMARD	PsA
Pakchotanon, 2020	1061	Employmen t	None	OR	0.34 (0.22-0.55)	cDMARD	PsA
Choi, 2020	363	CRP[12] (mg/L)		HR	1.08 (1.00-1.17)		AS
Pakchotanon, 2020	1061	CRP elevated		aOR	2.00 (1.04-3.85)	cDMARD	PsA
Pakchotanon, 2020	1061	ESR[13] (mm/hr)		OR	1.02 (1.00-1.03)	cDMARD	PsA
Anti-Tumour Necrosis Factor (TNF)							

Choi, 2020	363	Adalimumab	Other	HR	1.47 (0.96-2.24)		AS
Choi, 2020	363	Etanercept		HR	1.01 (0.64-1.60)		AS
Choi, 2020	363	Golimumab		HR	1.08 (0.52-2.23)		AS
Choi, 2020	363	Infliximab		HR	0.40 (0.20-0.88)		AS
Shelton, 2015*	96	Infliximab		OR	1.00 (0.13-7.87)	cDMARD	
Shelton, 2015*	96	Infliximab dose mg/kg		SMD	0.48 (0.07-0.89)		IBD
Shelton, 2015*	96	Infliximab frequency (weeks)		SMD	0.23 (-0.17-0.63)		IBD
Pakchotanon, 2020	1061	Anti-TNF	None	aOR	10.56 (3.63-30.69)	cDMARD	PsA
Parisi, 2016	305	Duration of infliximab		aOR	1.03 (1.01-1.05)	5-ASA, AZA	IBD
DMARDs							
Choi, 2020	363	cDMARDs	None	HR	1.08 (0.71-1.65)		AS
Choi, 2020	363	Prednisolone	None	HR	0.66 (0.38-1.13)		AS
Pakchotanon, 2020	1061	MTX[16]/LEF[17]	None	aOR	4.39 (1.67-11.54)		PsA
Shelton, 2015*	96	Thiopurines	None	OR	0.60 (0.19-1.82)	cDMARD	IBD
Shelton, 2015*	96	MTX	None	OR	0.49 (0.01-9.77)		IBD
Other drugs							
Choi, 2020	363	NSAID[18]	None	HR	0.96 (0.42-2.20)		AS
Choi, 2020	363	TB[19] prophylaxis	None	HR	1.2 (0.78-1.83)		AS
Choi, 2020	363	Statin	None	HR	0.72 (0.37-1.39)		AS
Pakchotanon, 2020	1061	NSAID	None	OR	0.63 (0.40-0.99)	cDMARD	PsA
Chiu, 2017	368	No folic acid with MTX	Yes	aOR	11.6 (2.52-56.46)	cDMARD	RA, PsA
HBV infection							
Chiu, 2017	368	HBsAg+[20]	-ve	aOR	7.91 (2.16-31.31)	cDMARD	RA, PsA
Chiu, 2018	368	HBsAg+	-ve	aOR	3.30 (1.30-8.20)	cDMARD	RA,PsA, PsO[22], AS
Chiu, 2018	368	HBsAg-/HBcAb+[21]	-ve	aOR	1.00 (0.40-2.30)	cDMARD	RA,PsA, PsO, AS
Chiu, 2017	368	HBsAg-/HBcAb+	-ve	aOR	1.00 (0.33-3.25)	cDMARD	RA, PsA

Hastings, 2017	367	Neutropenia (previous)	None	aHR	2.97 (1.69-5.25)	MTX	RA, AS, PsA
Hastings, 2017	367	Neutrophil count per 1 x 10^9/litre		aHR	0.60 (0.48-0.73)	MTX	
**Renal function**							
Swart, 2022	211	Risk factor for renal function decline		aβ	-0.029		AS

[1]RM: risk measure; [2]cDMARD: conventional DMARD; [3]RA: rheumatoid arthritis; [4]PsA: psoriatic arthritis; [5]AS: ankylosing spondylitis;[6]IBD: inflammatory bowel disease; [7]NAFLD: non-alcoholic fatty liver disease; [8]ALT: alanine transaminase; [9]ANA: anti-nuclear antibody; [10]RF: rheumatoid factor; [11]PsO: psoriasis; [12]CRP: C-reactive protein; [13]ESR: erythrocyte sedimentation rate; [14]5-ASA: 5-acetyl salicylic acid; [15]AZA: azathioprine; [16]MTX: methotrexate; [17]LEF: leflunomide; [18]NSAID: non-steroidal anti-inflammatory drug; [19]TB: tuberculosis; [20]HBsAg: hepatitis B surface antigen; [21]HBcAb: hepatitis B core antibody; [22]PsO: psoriasis; * calculated from crude data.

Table S11b. Summary of GRADE judgements: prognostic factors for hepatotoxicity and renal function in those prescribed anti-TNF alpha

Prognostic factor	Summary of findings	Quality of evidence	Reason for grading up or down
**Hepatotoxicity**			
Age	Reduced risk with increasing age. Evidence from two studies (Chiu, 2017; Shelton, 2015) with 464 participants.	Very low	Inconsistency
Sex	Evidence for increased risk for men. Evidence from three studies (Choi, 2020; Chiu, 2017; Shelton, 2015) with 731 participants.	Very low	Inconsistency
BMI	Evidence for increased risk. Evidence from two studies (Choi, 2020; Pakchotanon, 2020) with 1424 participants.	Low	
Smoking	No evidence for increased risk from one study Choi 2020 with 363 participants.	Very low	Single study
Alcohol	Evidence for increased risk from two studies (Choi, 2020; Pakchotanon, 2020) with 1424 participants.	Very low	Inconsistency
Comorbidities	Evidence for increased risk from two studies (Choi, 2020; Pakchotanon, 2020) with 1424 participants.	Moderate	Inconsistency downgrade one, large effect size upgrade two.
Liver disease, elevated liver enzymes	Evidence for increased risk from three studies (Choi, 2020; Chiu, 2017; Chiu 2018) with 731 participants.	High	Large effect
Serology	No evidence for risk from two studies (Choi, 2020 Pakchotanon, 2020) with 1424 participants.	Low	
Disease activity	Evidence for increased risk on some parameters. Evidence from two studies (Choi, 2020; Pakchotanon, 2020) with 1424 participants.	Very low	Inconsistency

Disease duration	Evidence for increased risk from one study Pakchotanon, 2020 with 1061 participants.	Very low	Single study
Disease type	No evidence for increased risk for any form of IBD. Evidence from one study of 96 patients (Shelton 2015)	Very low	Imprecision, single study
Individual anti-TNF	Evidence from two studies (Choi, 2020; Shelton, 2015), including459 participants that one anti-TNF is associated with elevated liver enzyme compared to other.	Very low	Inconsistency, imprecision
Other immune-suppressing treatments	Evidence of increased risk with methotrexate and leflunomide but not with azathioprine or prednisolone. From three studies (Choi, 2020; Pakchotanon, 2020; Shelton, 2015), including 15,923 participants.	Very low	Inconsistency, imprecision
Other drugs	No evidence of increased risk with co-prescription of NSAIDs, Statins, TB prophylaxis from three studies (Choi, 2020; Pakchotanon, 2020, Chui 2017) with 1424 participants.	Low	
**Nephrotoxicity**			
Risk factor for renal function decline	Evidence from one study (Swart, 2022) with 211 participants that risk factor for renal function decline is associated with risk of reduced renal function	Very low	Single study

Table S12a. Prognostic factors for cytopenia

Prognostic factor Author, year	N	Exposure	Referent	RM[1]	Point Estimate (95% CI)	Other treatment	Disease
Cytopenia							
Hastings, 2017	367	Neutropenia (previous)	None	aHR	2.97 (1.69-5.25)	MTX	RA, AS, PsA
Hastings, 2017	367	Neutrophil count per 1 x 10^9/litre		aHR	0.60 (0.48-0.73)	MTX	
**Neutropenia**							
Age							
AlAskar, 2020	281	Age ≥60 years		aOR	0.99 (0.97-1.02)		IBD
Hastings, 2010*	367	Age, mean years		SMD	0.06 (-0.2-0.32)		
Gender							
AlAskar, 2020	281	Gender		aOR	1.4 (0.82-2.4)		IBD
Hastings, 2010*	367	Female vs male sex		OR	1.43 (0.76-2.83)		RA, AS, PsA
Disease type							
Hastings, 2010*	367	AS vs RA		OR	0.81 (0.26-2.09)		
Hastings, 2010*	367	PsA vs RA					
AlAskar, 2020	281	CD vs UC		aOR	0.92 (0.41-2.09)		IBD
Disease duration							
Hastings, 2010*	367	Disease duration, mean		SMD	0.08 (-0.18-0.35)		RA, AS, PsA
First Anti-TNF agent							
AlAskar, 2020	281	First agent		aOR	0.84 (0.7-1.02)		IBD
Concomitant immune-suppressing medication							
AlAskar, 2020	281	AZA		aOR	2.32 (1.26-4.28)		IBD
AlAskar, 2020	281	MTX		aOR	0.95 (0.99-9.09)		
AlAskar, 2020	281	5-ASA		aOR	3.15 (1.55-6.39)		
Hastings, 2010*	367	MTX vs no MTX		OR	0.97 (0.52-1.75)		RA, AS, PsA
Hastings, 2010*	367	Prednisolone vs none		OR	0.68 (0.33-1.33)		RA, AS, PsA
Serology							
Hastings, 2010*	367	ANA pos vs ANA neg		OR	1.33 (0.7-2.15)		RA, AS, PsA
Hastings, 2010*	367	RF pos vs RF neg		OR	1.23 (0.7-2.15)		RA, AS, PsA
Drug type							
Hastings, 2010*	367	Etanercept vs adalimumab		OR	0.94 (0.35-2.94)		RA, AS, PsA
Hastings, 2010*	367	Infliximab vs adalimumab		OR	1.06 (0.33-3.77)		RA, AS, PsA

*calculated from crude data

Table S12b. Summary of GRADE judgements: prognostic factors for cytopenia and neutropenia in patients prescribed anti-TNF alpha.

	Summary of findings	Quality of evidence	Reason for grading up or down
Marrow function	Evidence of increased risk of cytopenia from previous neutropenia and reduced risk with increasing baseline neutrophil count. Evidence from one study (Hastings, 2017), including 367 participants.	Low	Large effect upgrade two levels, downgrade one level due to concern about study quality, downgrade one for single study
Age	Evidence from two studies (Alaskar, 2020; Hastings 2010), with 648 participants that age does not associate with increased risk of neutropenia	Low	
Gender	No evidence from two studies (Alaskar, 2020; Hastings 2010), with 648 participants that gender associates with increased risk of neutropenia	Low	
Disease type	No evidence from two studies (Alaskar, 2020; Hastings 2010), with 648 participants that disease type associates with increased risk of neutropenia	Low	
Disease duration	No evidence from one study (Hastings 2010) with 367 participants that disease duration is associated with a reduced risk of neutropenia.	Very low	Single study
First Anti-TNF agent	No evidence from one study (Alaskar, 2020), with 281 participants that first Anti-TNF agent is associated with risk of neutropenia	Very low	Single study
Concomitant immune suppressant	Evidence from two studies (Alaskar, 2020; Hastings 2010), with 648 participants that concomitant immune suppressant medication is associated with risk of neutropenia.	Very low	Inconsistent
Serology	No evidence from one study (Hastings, 2010), with 367 participants that ANA positive or RF positive is associated with risk of neutropenia	Very low	Single study
Drug type	No evidence from one study (Hastings, 2010) with 367 participants drug type is associated with risk of neutropenia	Very low	Single study

Table S13a: Prognostic factors for composite toxicity (myelotoxicity and/or hepatotoxicity) in people with inflammatory bowel disease prescribed thiopurines.

Author, year	N	Exposure	Referent	RM[1]	Point estimate (95%CI)	Other treatment
Kreijne, 2020	1132	Male	Female	aHR	1.29 (1.09-1.52)	+/- 5-ASA[2]
Kreijne, 2020	1132	Smoker	Non-smoker	HR	0.80 (0.64-1.01)	+/- 5-ASA
Kreijne, 2020	1132	Ulcerative colitis	Crohn's disease	aHR	1.25 (1.06-1.49)	+/- 5-ASA
Kreijne, 2020	1132	5-ASA	None	HR	1.12 (0.90-1.39)	+/- 5-ASA
Kreijne, 2020	1132	Mercaptopurine	Azathioprine	aHR	1.56 (1.30-1.87)	+/- 5-ASA
Kreijne, 2020	1132	Allopurinol	None	HR	1.45 (0.93-2.27)	+/- 5-ASA
Kreijne, 2020	1132	Previous surgery	None	HR	0.90 (0.75-1.09)	+/- 5-ASA

[1]RM: Risk measure; [2]5-ASA: 5-acetyl salicylic acid.

Table S13b: Summary of GRADE judgements: Prognostic factors for composite toxicity (myelotoxicity and/or hepatotoxicity) in people with inflammatory bowel disease prescribed thiopurines.

	Summary of findings	Quality of evidence	Reason for grading up or down
Sex	Evidence from one study (Kreijne, 2020) with 1132 participants, of increased risk for males	Very low	Single study
Smoking status	No evidence of increased risk from one study (Kreijne, 2020) with 132 participants for smokers	Very low	Single study
IBD type	Evidence from one study (Kreijne, 2020) with 1132 participants, of increased risk for UC over CD	Very low	Single study
5-ASA	No evidence from one study (Kreijne, 2020) with 1132 participants, of increased risk with 5-ASA	Very low	Single study
Mercaptopurin e	Evidence from one study (Kreijne, 2020) with 1132 participants, of increased risk for mercaptopurine over azathiopine	Very low	Single study
Allopurinol	No evidence from one study (Kreijne, 2020) with 1132 participants, of increased risk with allopurinal use	Very low	Single study
Previous surgery	No evidence from one study (Kreijne, 2020) with 1132 participants, of increased risk for those with a prior history of surgery	Very low	Single study

Table S14a: Prognostic factors for cytopenia in those prescribed thiopurines

Prognostic factor Author, year	N	Exposure	Referent	RM[1]	Point estimate (95% CI)	Other treatment
Age						
Calafat, 2019	19630	>=60 year	18-50 year	aHR	2.22 (1.91-2.57)	
Banerjee, 2020	935	Age per year increase		aHR	0.988 (0.97-1.007)	
Broekman 2017*	695	Age per year increase		SMD	0.1 (-0.2-0.4)	
Kim, 2017*	1098	Age per year increase		SMD	0.17 (-0.07-0.42)	
Age at disease onset						
Labidi, 2020*	210	Age per year increase		SMD	0.27 (-0.15-0.69)	
Labidi, 2020*	210	Age <20 years	Age ≥20 years	OR	0.81 (0.09-3.75)	
Sex						
Banerjee, 2020	935	Male	Female	HR	1.17 (0.75-1.83)	
Broekman 2017*	695	Male	Female	OR	1.09 (0.57-2.09)	
Chapparo, 2013	3931	Female	Male	aHR	3.60 (2.00-6.00)	
Kim, 2017	1098	Female	Male	aOR	1.65 (0.91-3.01)	
Calafat, 2019	19630	Female	Male	aHR	1.20 (1.08-1.34)	
Kreijne, 2020	1132	Male	Female	HR	1.13 (0.92-1.40)	+/- 5-ASA[2]
Park, 2016[e]	964	Female	Male	OR	1.42 (1.06-1.90)	+/- 5-ASA[2]; Anti-TNF
Park, 2016[f]	964	Male	Female	OR	0.5 (0.29-0.88)	
Park, 2016[g]	964	Male	Female	OR	0.77 (0.56-1.08)	
Labidi, 2020	210	Male	Female	OR	2.49 (0.93-7.37)	
BMI/Weight						
Kim, 2017	1098	<50 kg	≥50 kg	aOR	1.52 (0.84-2.78)	
Broekman, 2017*	695	BMI per kg/m^2 increase		SMD	0.02 (-0.28-0.32)	
Banerjee, 2020	935	BMI per kg/m^2 increase		HR	0.99 (0.97-1.02)	
Lifestyle						
Kreijne, 2020	1132	Smoker	None	HR	0.80 (0.60-1.07)	+/- 5-ASA
Banerjee, 2020	935	Smoker	None	HR	0.44 (0.16-1.22)	
Broekman, 2017	695	Smoker	None	HR	0.37 (0.13-1.03)	+/- 5-ASA, Anti-TNF
Labidi, 2020	210	Smoker	None	OR	0.33 (0.06-1.18)	
Comorbidity						
Broekman, 2017*	695	Diabetes mellitus		OR	2.04 (0.38-7.2)	
Broekman, 2017*	695	Rheumatic disease		OR	0.79 (0.9-3.27)	
Broekman, 2017*	695	Asthma/COPD		OR	0.63 (0.12-2.07)	
Calafat, 2019	19630	CV risk factors	None	aHR	1.20 (1.01-1.42)	
Calafat, 2019	19630	Hyperuricaemia	None	aHR	2.39 (1.44-3.97)	
Cytopenia						
Banerjee, 2020	935	Baseline WBC count		HR	1 (0.81-1.3)	
Broekman, 2017	695	WBC count, 10^9/L (increase by 1)		aHR	0.80 (0.71-0.89)	+/- 5-ASA +/- Anti-TNF

Broekman, 2017*	695	Wk 1 decrease WBC count, 109/L, mean (SD)		SMD	0.4 (0.1-0.7)	
Kim, 2017*	1098	Baseline WBC count		SMD	0.03 (-0.22-0.27)	
Kim, 2017*	1098	Baseline neutrophil count		SMD	0.2 (-0.04-0.44)	
Kim, 2017*	1098	Baseline lymphocyte count		SMD	0.1 (-0.15-0.34)	
Kim, 2017*	1098	Anaemia (Hb < 12 gm/dl)		aOR	1.39 (0.81-2.42)	
Kim, 2017*	1098	Baseline platelet count		SMD	0.08 (-0.17-0.32)	
Inflammatory bowel disease type						
Banerjee, 2020*	935	CD	Other IBD	HR	0.89 (0.57-1.39)	
Broekman, 2017*	695	CD		OR	1.03 (0.54-1.99)	
Calafat, 2019	19630	CD[3]	UC[4]	aHR	0.83 (0.74-0.93)	
Kreijne, 2020	1132	UC	CD	HR	1.10 (0.89-1.35)	+/- 5-ASA
Labidi, 2020*	210	IC	Other IBD	OR	0.66 (0.01-4.93)	
Labidi, 2020*	210	UC	Other IBD	OR	1.34 (0.31-4.49)	
Labidi, 2020*	210	CD	Other IBD	OR	0.97 (0.32-3.52)	
Kim, 2017*	1098	CD	Any IBD	OR	1.34 (0.79-2.27)	
Kim, 2017*	1098	UC	Any IBD	OR	0.6 (0.32-1.1)	
Kim, 2017*	1098	Intestinal Behcets Disease	Any IBD	OR	1.21 (0.58-2.5)	
IBD severity						
Broekman, 2017*	695	CD, Harvey Bradshaw Index, mean (SD)		SMD	0.03 (-0.27-0.34)	
Broekman, 2017*	695	UC, partial Mayo, mean (SD)		SMD	0.03 (-0.27-0.34)	
Kreijne, 2020	1132	Previous surgery	None	HR	0.96 (0.75-1.19)	+/- 5-ASA
Labidi, 2020*	210	Exten. Ileal dis.	None	OR	1.28 (0.29-4.26)	
Labidi, 2020*	210	Complex perianal fistulas	None	OR	1.52 (0.15-7.81)	
Labidi, 2020*	210	Corticosteroid dependence	No	OR	2.53 (0.86-6.88)	
Labidi, 2020*	210	Corticosteroid resistance	No	OR	0.76 (0.27-1.96)	
Labidi, 2020*	210	Disease duration before thiopurine (months)		SMD	0.35 (-0.07-0.77)	
Kim, 2017*	1098	Steroid sparing		OR	1.25 (0.64-2.49)	
Kim, 2017*	1098	Fistula		OR	0.61 (0.26-1.39)	
Kim, 2017*	1098	Active disease		OR	1.39 (0.44-4.4)	
Immune suppressing drugs						
Broekman, 2017	695	Anti-TNF	None	aHR	1.67 (0.80-3.49)	+/- 5-ASA +/- Anti-TNF
Park, 2016[e]	964	Anti-TNF	None	OR	0.47 (0.05-4.26)	+/- 5-ASA[2]; Anti-TNF
Park, 2016[f]	964	Anti-TNF	None	OR	2.42 (0.05-24.83)	
Labidi, 2020*	210	Anti-TNF	None	OR	0.68 (0.07-3.1)	
Broekman, 2017*	695	5-ASA		OR	1.29 (0.67-2.5)	

Broekman, 2017*	695	Corticosteroids		OR	1.13 (0.59-2.22)	
Banerjee, 2020	935	5-ASA	None	HR	0.90 (0.56-1.44)	
Banerjee, 2020	935	Corticosteroids	None	HR	0.68 (0.44-1.06)	
Banerjee, 2020	935	Biologics	None	HR	1.30 (0.47-3.54)	
Fangbin, 2012	199	5-ASA	None	OR	3.91 (1.85-8.26)	
Kreijne, 2020	1132	5-ASA	None	HR	1.04 (0.82-1.33)	+/- 5-ASA
Park, 2016[e]	964	5-ASA	None	OR	1.02 (0.66-1.58)	+/- 5-ASA[2]; Anti-TNF
Park, 2016f	964	5-ASA	None	OR	1.81 (0.64-7.05)	
Park, 2016g	964	5-ASA	None	OR	0.92 (0.57-1.5)	
Labidi, 2020*	210	5-ASA	None	OR	0.95 (0.38-2.4)	
Labidi, 2020*	210	Corticosteroids	No	OR	1.14 (0.35-4.87)	
Park, 2016[f]	964	Corticosteroids	No	OR	1.07 (0.6-1.87)	
Park, 2016[g]	964	Corticosteroids	No	OR	1.09 (0.8-1.49)	
Park, 2016[e]	964	Corticosteroids	No	OR	1.09 (0.82-1.44)	+/- 5-ASA[2]; Anti-TNF
Interacting drugs						
Houwen, 2021[a]	37360	Allopurinol	None	aHR	0.92 (0.82-1.03)	
Houwen, 2021[b]	37360	Allopurinol	None	aHR	0.96 (0.89-1.03)	
Houwen, 2021[c]	37360	Allopurinol	None	aHR	0.94 (0.81-1.09)	
Houwen, 2021[d]	37360	Allopurinol	None	aHR	1.03 (0.93-1.15)	
Houwen, 2021[e]	37360	Allopurinol	None	aHR	0.87 (0.82-0.93)	
Kreijne, 2020	1132	Allopurinol	None	HR	1.59 (1.04-2.43)	+/- 5-ASA
Broekman, 2017*	695	ACE inhibitor		OR	1.47 (0.16-6.36)	
Thiopurine type						
Chapparo, 2013	3931	Mercaptopurine	Azathioprine	aHR	5.00 (2.50-11.00)	
Calafat, 2019	19630	Mercaptopurine	Azathioprine	aHR	1.86 (1.55-2.24)	
Kreijne, 2020	1132	Mercaptopurine	Azathioprine	HR	1.02 (0.81-1.29)	+/- 5-ASA
Broekman, 2017	695	Mercaptopurine	Azathioprine	aHR	2.61 (1.39-4.88)	
Thiopurine dose						
Broekman, 2017	695	Dose (mg/kg)		HR	5.59 (0.68-46.10)	+/- 5-ASA +/- Anti-TNF
Labidi, 2020*	210	Azathioprine (mg/kg/day)		SMD	-0.21 (-0.63-0.21)	
Kim, 2017*	1098	Azathioprine (mg/kg)		SMD	0.04 (-0.2-0.29)	
Genotype and/or metaboliser status [Thiopurine methyl-transferase (TPMT), NUDT15 gene]						
Dickson, 2022	1403	Poor/intermediate metaboliser status[e]	Normal/indeterminate metaboliser status[f]	aHR	2.67 (1.44-4.94)	
Broekman, 2017	695	TPMT activity, mg/mmol Hb.h, mean (SD)		SMD	0.07 (-0.23-0.37)	
Fangbin, 2012	199	TMTP-Low	TMTP-High	OR	20.25 (2.19-187.17)	
Kim, 2017	286	TPMT *1/*3C	TPMT wild	aRR	2.84 (1.15-7.02)	+ /- 5-ASA
Kim, 2017	286	TPMT-High	TPMT-Low	aRR	0.65 (0.40-1.06)	+/- 5-ASA
Kim, 2017	286	TPMT- Intt	TPMT-Low	aRR	0.55 (0.35-0.86)	+/- 5-ASA

Fangbin, 2012	199	TPMT-Low	High TPMT	OR	34.80 (3.71-326.77)
Banerjee, 2020	935	CT genotype	CC genotype	aHR	11.111 (6.848-18.029
Banerjee, 2020	935	TT genotype	CC genotype	aHR	31.283 (14.759-66.305)

[1]RM: Risk measure; [2]5-ASA: 5-acetyl salicylic acid;[3]CD: Crohn's disease; [4]UC: Ulcerative Colitis; In the study by Park et al, early and late leucopenia were grouped into one; Banerjee 2020 outcome is leucopenia; Houwen 2021[a] outcome is neutropenia; Houwen 2021[b] outcome is myelosuppression; Houwen 2021[c] outcome is thrombocytopenia; Houwen 2021[d] outcome is erythocytopenia; Houwen 2021[e] outcome is composite myelotoxicity (leukopenia, thrombocytopenia, erythrocytopenia).
Dickson 2022 population is mixed SLE, RA and IBD.; [f]based on TPMT and NUDT15 gene activity; Broekman 2017 outcome is leucopenia; Labidi 2020 outcome is myelotoxicity; Park 2016 outcome is leucopenia; Park 2016[e] outcome is for any vs no leucopenia; Park 2016[f] outcome is for early vs no leucopenia; Park 2016[g] outcome is for late versus no leucopenia; *calculated from crude data.

Table S14b. Summary of GRADE judgements: Prognostic factors for cytopenia in inflammatory bowel disease prescribed thiopurines

Prognostic factor	Summary of findings	Quality of evidence	Reason for grading up or down
Age	Increased risk with increasing age. Evidence from four studies (Calafat, 2019; Banerjee, 2020; Broekman, 2017; Kim, 2017) with 22,358 participants.	Very low	Inconsistency
Age at disease onset	No evidence from one study (Labidi, 2020) with 210 participants that age at onset associates with increased risk of cytopenia.	Very low	Single study, inconsistency (within study)
Sex	Evidence of increased risk for female gender. Evidence from eight studies (Chaparro, 2013; Park 2016; Calafat, 2019; Kreijne, 2020; Kim 2017; Labidi 2020; Banerjee, 2020; Broekman, 2017) with 28,888 participants.	Low	Increased for large effect size, reduced for inconsistency
BMI/weight	No evidence for increased risk. Evidence from three studies (Kim, 2017; Broekman, 2017; Banerjee, 2020) with 2,728 participants.	Very low	Imprecision
Smoking	No evidence for increased risk from four studies (Kreijne, 2020; Broekman, 2017; Labidi 2020; Banerjee, 2020) with 2972 participants. Potential for protective effect.	Low	
Comorbidities	Evidence for increased risk from two studies (Calafat, 2019; Broekman, 2017) with 20,325 participants.	Very low	Inconsistency
Cytopenia	Evidence for increased risk for low WBC count from three studies (Broekman, 2017; Banerjee, 2020; Kim, 2017) with 2,728 participants. No evidence for increased risk with anaemia or thrombocytopenia.	Very low	Inconsistency
IBD type	Evidence of reduced risk with CD, evidence from four studies (Calafat, 2019; Kreijne, 2020; Labidi, 2020; Kim, 2017) with 22,280 participants.	Very low	Inconsistency
Disease activity	No evidence for increased risk. Evidence from four studies (Broekman, 2017; Kreijne, 2020; Kim, 2017; Labidi 2020) with 2340 participants.	Low	
Immune suppressing drugs	Evidence from six studies (Broekman, 2017; Fangbin, 2012; Kreijne, 2020; Labidi, 2020, Park 2016, Banerjee 2020), including 4135 participants that co-prescription of Anti-TNF, 5-ASA, prednisolone, does not increase risk of cytopenia.	Very low	Inconsistency
Mercaptopurine	Evidence of increased risk compared to azathioprine. From four studies (Chapparo, 2013; Calafat, 2019; Kreijne, 2020; Broekman, 2017), including 25,388 participants.	High	Large consistent effect in three of the four included studies, upgrade two

Allopurinol	Evidence of increased risk from two studies (Kreijne, 2020; Houwen, 2021), including 38,495 participants	Very low	Inconsistency
Poor thiopurine metaboliser based on TPMT/NUDT genotype +/- intermediate or low enzyme activity	Evidence of increased risk from four studies (Fangbin, 2012; Kim, 2017; Banerjee 2020; Dickson, 2022), including 2823 participants. Broekman 2017 (n=695) showed no evidence of increased risk but they only included patients with wild type TPMT genotype.	High	Large effect, upgrade two. Upgrade one dose response. Downgrade for Broekman 2017 due to selection bias on this exposure and outcome.

Table S15a: Prognostic factors for hepatotoxicity in those prescribed thiopurines

Prognostic factor Author, year	N	Exposure	Referent	RM[1]	Point estimate (95% CI)	Other treatment
Age						
Calafat, 2019	19630	>=60 years	18-50 years	aHR	2.07 (1.72-2.50)	
Cheng, 2022	227	Age per year increase	None	aHR	0.97 (0.84-1.11)	
Labidi, 2020*	210	Age at disease onset (years)		SMD	0.45 (-0.26-1.16)	
Wong, 2017	270	Age, >50 years		aOR	4.5 (2.2-9.3)	
Sex						
Chaparro, 2013	3931	Female	Male	aHR	0.60 (0.40-0.90)	
Calafat, 2019	19630	Female	Male	aHR	0.85 (0.74-0.97)	
Cheng, 2022	227	Male	Female	aHR	1.22 (0.84-1.78)	
Kreijne, 2020	1132	Male	Female	aHR	1.13 (0.90-1.42)	+/- 5-ASA
Labidi, 2020*	210	Male	Female	OR	2.34 (0.33-26.35)	
Wong, 2017	270	Male	Female	aOR	2.50 (1.30-5.10)	
Race						
Cheng, 2022	227	White race	Non-white	aHR	0.96 (0.66-1.41)	
Body mass index (BMI)						
Cheng, 2022	227	BMI per kg/m^2 increase		HR	1.01 (0.98-1.5)	
Wong, 2017	270	BMI >25 kg/m^2	=<25 kg/m^2	aOR	3.90 (1.90-8.10)	
Lifestyle						
Cheng, 2022	227	Current alcohol abuse	None	HR	0.88 (0.6-1.29)	
Cheng, 2022	227	Current smoker	None	HR	1.44 (0.95-2.2)	
Kreijne, 2020	1132	Current smoker	None	HR	1.12 (0.83-1.51)	+/- 5-ASA
Labidi, 2020*	210	Current smoker	None	OR	0.52 (0.01-4.82)	
Comorbidity						
Cheng, 2022	227	HIV	None	HR	1.91 (0.78-4.7)	
Cheng, 2022	227	Hypertension	None	HR	1.23 (0.74-2)	
Cheng, 2022	227	Diabetes	None	HR	1.58 (0.5-5)	
Cheng, 2022	227	Hyperlipidemia	None	HR	0.85 (0.49-1.49)	
Calafat, 2019	19630	Cardiovascular risk	None	aHR	1.41 (1.15-1.73)	
Liver						
Parisi, 2016	305	ALT elevation	None	aOR	3.85 (1.80-8.25)	
Inflammatory bowel disease (IBD) type						
Chaparro, 2013	3931	CD[3]	UC[4]	aHR	3.30 (2.50-4.50)	
Calafat, 2019	19630	CD	UC	aHR	0.66 (0.57-0.76)	
Cheng, 2022	227	Other IBD (mainly UC)	CD	aHR	0.66 (0.46-0.95)	
Kreijne, 2020	1132	UC	CD	aHR	1.07 (0.84-1.35)	+/- 5-ASA
Labidi, 2020*	210	CD		OR	1.23 (0.13-59.82)	
Labidi, 2020*	210	UC		OR	1.35 (0.03-12.79)	
Wong, 2022*	270	CD	UC	HR	0.74 (0.37-1.45)	
Disease duration						
Labidi, 2020*	210	Disease duration (months)		SMD	-0.69 (-1.40-0.015)	

Wong, 2017*	270	Disease duration (years)		SMD	0.16 (-0.16-0.47)	
Cheng, 2022	227	Disease duration (years)		aHR	0.99 (0.97-1.02)	
IBD severity						
Calafat, 2019	19630	Extra-intestinal manifestations	None	aHR	1.22 (1.05-1.43)	
Kreijne, 2020	1132	Intestinal surgery	None	HR	0.89 (0.70-1.15)	+/- 5-ASA
Cheng, 2022	227	Intestinal surgery	None	HR	1.28 (0.75-2.2)	
Cheng, 2022	227	Extensive UC	left-sided colitis or ulcerative proctitis	HR	1.88 (1.1-3.2)	
Cheng, 2022	227	Ileocolonic CD	Other distributions of CD	HR	1.04 (0.61-1.77)	
Cheng, 2022	227	Penetrating CD	No complications of CD	HR	2 (1.03-4)	
Labidi, 2020*	210	Corticosteroid dependence	No	OR	0.95 (0.02-8.89)	
Labidi, 2020*	210	Corticosteroid resistance	No	OR	3.46 (0.48-38.86)	
Immune suppressing drugs (concomitant)						
Cheng, 2022	227	5-ASA	None	HR	0.9 (0.62-1.31)	
Cheng, 2022	227	Topical medication	None	HR	0.57 (0.32-1.01)	
Cheng, 2022	227	Immunomodulato r	None	HR	0.33 (0.08-1.33)	
Cheng, 2022	227	Biologic	None	HR	0.63 (0.2-1.99)	
Cheng, 2022	227	Corticosteroid	None	HR	0.77 (0.44-1.32)	
Labidi, 2020*	210	Anti-TNF	None	OR	1.64 (0.03-15.62)	
Kreijne, 2020	1132	5-ASA	None	HR	0.83 (0.62-1.10)	
Labidi, 2020*	210	Cyclosporine	None	OR	3.47 (0.07-34.95)	
Labidi, 2020*	210	5-ASA	None	OR	1.04 (0.14-7.95)	
Wong, 2017*	270	5-ASA	None	OR	1.11 (0.56-2.24)	
Wong, 2017*	270	Corticosteroids	None	OR	1.54 (0.59-4.72)	
Wong, 2017*	270	Biologic (infliximab, adalimumab)	None	OR	1.64 (0.37-5.73)	
Thiopurine type (Mercaptopurine vs. azathioprine)						
Calafat, 2019	19630	Mercaptopurine	Azathioprine	aHR	1.71 (1.34-2.17)	
Kreijne, 2020	1132	Mercaptopurine	Azathioprine	aHR	1.46 (1.15-1.85)	+/- 5-ASA
Wong, 2017*	270	Mercaptopurine	Azathioprine	OR	2.14 (1.06-4.26)	
Thiopurine dose						
Labidi, 2020*	210	Azathioprine dose (mg/kg/day)		SMD	0.71 (-0.01-1.41)	
Wong, 2017*	270	Azathioprine dose (mg/kg/day)		SMD	0.12 (-0.19-0.44)	
Wong, 2017*	270	Mercaptopurine dose (mg/kg/day)		SMD	0.50 (0.18.-0.82)	
Other drugs(interacting)						
Houwen, 2021	37360	Allopurinol	None	aHR	0.42 (0.3-0.6)	
Kreijne, 2020	1132	Allopurinol	None	HR	1.75 (1.16-2.66)	+/- 5-ASA

Biomarkers						
Wong, 2017	270	6-MMPR[3] >3615 Pmol at week 1 of treatment	<= 3615 pmol	aOR	3.80 (1.80-8.00)	+/- 5-ASA +/- Anti-TNF
TMPT activity						
Wong, 2017*	270	TPMT enzyme activity (mg 6-MTG/mmol Hb/h)		SMD	0.06 (-0.25-0.38)	

[1]RM: Risk measure; [2]5-ASA: 5-acetyl salicylic acid;[3]CD: Crohn's disease; [4]UC: Ulcerative Colitis;[5]IBDQ: IBD questionnaire. Data (n ,%) from Labidi 2020 were not transformed to OR as the total number of people developing hepatotoxicity reported in the text did not correspond to the data reported in the tables; *calculated from crude data

Table S15b. Summary of GRADE judgements: Prognostic factors for hepatotoxicity in inflammatory bowel disease patients prescribed thiopurines

	Summary of findings	GRADE Quality of evidence	Reason for grading up or down
Age	Evidence from four studies (Calafat, 2019; Wong, 2017; and Cheng, 2022; Labidi, 2020) including 20,337 participants that age increases the risk of elevated liver enzymes.	Moderate	Large effect, upgrade two, downgrade one for inconsistency
Sex	Evidence from six studies (Chaparro, 2013; Calafat, 2019; Kreijne, 2020; Cheng, 2022; Labidi, 2020; and Wong, 2017) with 25,400 participants that male sex increases the risk of elevated liver enzymes.	Moderate	Large effect, upgrade two inconsistent downgrade one
BMI	Evidence from two studies (Cheng, 2022; Wong, 2017) with 497 participants that high BMI associates with elevated liver enzymes.	Very low	Inconsistency
Race	No evidence from one study (Cheng, 2022) with 227 participants that race associates with elevated liver enzymes	Very low	Single study - imprecision
Smoking	Evidence from three studies (Kreijne, 2020; Cheng, 2022; Labidi 2020) with 1569 participants that cigarette smoking does not associate with elevated liver enzymes.	Moderate	
Alcohol abuse	No evidence from one study (Cheng, 2022), with 227 participants, that current alcohol abuse is associated with elevated liver enzymes.	Very low	Single study
Comorbidities	Evidence from one study (Calafat, 2019) with 19,630 participants that CV risk associates with elevated liver enzymes; no evidence from one study (Cheng, 2022), with 227 participants, that hypertension, HIV, hyperlipidemia or diabetes associates with elevated liver enzymes.	Very low	Imprecision
ALT elevation	Evidence from one study (Parisi, 2016) with 305 participants that ALT elevation is prognostic for elevated liver enzymes.	Moderate	Large effect upgrade two, single study downgrade one
IBD type	Evidence from six studies (Chaparro, 2013; Calafat, 2019; Kreijne, 2020; Cheng, 2022; Labidi 2020; Wong, 2022) with 25,400 participants that IBD type is a prognostic factor for elevated liver enzymes with conflicting evidence for CD and UC.	Very low	Inconsistency
IBD severity	vidence from four studies (Calafat, 2019; Kreijne, 2020; Cheng, 2022) with 22,999 participants that IBD severity is a prognostic factor for elevated liver enzymes.	Very low	Inconsistency

Other immunosuppressant	Evidence from four studies (Cheng, 2022; Labidi, 2020; Wong, 2017 and Kreijne, 2020), including 3,181 participants that co-prescription of Anti-TNF, 5-ASA, prednisolone, and other treatments does not increase risk of elevated liver enzymes	Low	
Thiopurine type	Evidence from three studies (Calafat, 2019; Kreijne, 2020, and Wong, 2017), including 21,032 participants that mercaptopurine carries a higher risk than azathioprine.	High	Large effect size upgrade two
Azathioprine dose	No evidence from two studies (Labidi, 2020; Wong, 2017), including 480 participants, that azathioprine dosage increases the risk of elevated liver enzymes.	Low	
Mercaptopurine dose	Evidence from one study (Wong, 2017), including 270 participants, that mercaptopurine dosage increases the risk of elevated liver enzymes		
Allopurinol	Evidence from two studies (Kreijne, 2020; Houwen, 2021), including 38,492 participants that co-prescription of allopurinol reduces risk of elevated liver enzymes.	Very low	Inconsistent
Biomarkers	Evidence from one study (Wong, 2017), including 270 participants, that 6-MMPR[3] >3615pmol increases the risk of elevated liver enzymes.	Very low	Single study - imprecision
TMPT activity	Evidence from one study (Wong, 2017), including 270 participants, that TPMT enzyme activity (mg 6-MTG/mmol Hb/h) has no effect on the risk of elevated liver enzymes.	Very low	Single study - imprecision

Table S16a: Prognostic factors for either cytopenia, nephrotoxicity, or elevated liver enzymes* in people with inflammatory bowel disease prescribed azathioprine

Author, year	N	Exposure	Referent	RM[1]	Point estimate (95%CI)
Fraser, 2022	8597	40-59 years	18-39 years	aOR	1.35 (1.03-1.79)
Fraser, 2022	8597	60-79 years	18-39years	aOR	0.65 (0.45-0.93)
Fraser, 2022	8597	≥80 years	18-39 years	aOR	0.09 (0.01-0.33)
Fraser, 2022	8597	Female sex	Male sex	aOR	0.96 (0.75-1.22)
Fraser, 2022	8597	IMD[2] quintile 4	5 least deprived	aOR	1.41 (0.99-2.01)
Fraser, 2022	8597	IMD quintile 3	5 least deprived	aOR	0.90 (0.63-1.28)
Fraser, 2022	8597	IMD quintile 2	5 least deprived	aOR	0.83 (0.57-1.22)
Fraser, 2022	8597	IMD quintile 1	5 least deprived	aOR	0.85 (0.57-1.26)
Fraser, 2022	8597	CKD[3]	No CKD	aOR	0.39 (0.17-0.82)
Fraser, 2022	8597	Diabetes T1 or T2	No diabetes	aOR	0.57 (0.35-0.94)
Fraser, 2022	8597	Hypertension	No hypertension	aOR	0.91 (0.61-1.36)

[1]RM: Risk measure the outcome was normal blood-test result during azathiopurine prescription; [2]IMD: index of multiple deprivation;[3]CKD:chronic kidney disease. * within specified NICE thresholds and tests (test thresholds: WBC ≥3.5×10 9/L; MCV ≤105 fL; neutrophils ≥1.6×10 9/L;platelets ≥140×10 9/L; eosinophils ≤0.5×10 9/L; ALT ≤100 U/L; AST ≤100 U/L; albumin ≥30 g/L; and eGFR ≥60 ml/min/1.73 m2).

Table S16b: Prognostic factors for either cytopenia, nephrotoxicity, or elevated liver enzymes in people with inflammatory bowel disease prescribed azathioprine

	Summary of findings	Quality of evidence	Reason for grading up or down
Age	Evidence of increased risk for 40-59 year old but reduced risk for >60 year old compared to 18-39 year old from one study (Fraser, 2022) with 8597 participants.	Very low	Single study, inconsistent effect.
Sex	Evidence of no association between sex and increased risk of either cytopenia, nephrotoxicity, elevated liver enzymes from one study (Fraser, 2022) with 8597 participants.	Very low	Single study
Comorbidity	Evidence of increased risk for those with comorbid CKD or diabetes, but no association with comorbid hypertension from one study (Fraser, 2022) with 8597 participants.	Very low	Single study
Deprivation	Evidence of no association between deprivation level and increased risk of either cytopenia, nephrotoxicity, elevated liver enzymes from one study (Fraser, 2022) with 8597 participants.	Very low	Single study

Table S17a: Prognostic factors for acute kidney injury in inflammatory bowel disease patients prescribed thiopurines

Prognostic factor Author, year	N	Exposure	Referent	RM[1]	Point estimate (95% CI)
Age					
Achit, 2020	94363	Age per 1-year increase	None	aHR	1.01 (1-1.01)
Sex					
Achit, 2020	94363	Female	Male	aHR	2.02 (1.64-2.49)
Inflammatory bowel disease type					
Achit, 2020	94363	Crohn's Disease	Ulcerative Colitis	aHR	1.31 (1.08-1.6)
Comorbidity					
Achit 2020	94363	Diabetes		aHR	1.83 (1.33-2.5)
Achit 2020	94363	Recent stroke		aHR	2.1 (1.03-4.3)
Achit 2020	94363	Dyslipidemia		aHR	1.95 (1.41-2.7)
Achit 2020	94363	Arterial disease		aHR	3.06 (1.24-7.53)
Achit 2020	94363	Heart failure		aHR	3.31 (1.77-6.17)

[1]RM: Risk measure; [2]5-ASA: 5-acetyl salicylic acid;[3]CD: Crohn's disease; [4]UC: Ulcerative Colitis;[5]IBDQ: IBD questionnaire.

Table S17b. Summary of GRADE judgements: Prognostic factors for acute kidney injury in inflammatory bowel disease patients prescribed thiopurines

	Summary of findings	Quality of evidence	Reason for grading up or down
Age	Evidence that age increases the risk of acute kidney injury from one study (Achit, 2020) including 94,363 participants.	Very low	Single study, imprecision
Sex	Evidence that being female compared to male sex increases the risk of acute kidney injury from one study (Achit, 2020) including 94,363) participants.	Very low	Single study, imprecision
Comorbidities	Evidence that diabetes, hyperlipidaemia and cardiovascular diseases increases the risk of acute kidney injury from one study (Achit, 2020) including 94,363) participants.	Very low	Single study, imprecision
Inflammatory bowel disease type	Evidence that Crohn's Disease increases the risk of acute kidney injury from one study (Achit, 2020) including 94,363) participants.	Very low	Single study, imprecision

Table S18a: Prognostic factors for either cytopenia, acute kidney injury, or elevated liver enzymes toxicity in people with autoimmune rheumatic diseases prescribed Leflunomide

Author, year	N	Exposure	Referent	RM[1]	Point estimate (95%CI)
Nakafero, 2022b	1487	Age per year increase		aHR	1.01 (0.99-1.03)
Nakafero, 2022b	1487	Female sex	Male sex	aHR	1.24 (0.83-1.83)
Nakafero, 2022b	1487	BMI per kg/m^2 increase		aHR	0.98 (0.95-1.01)
Nakafero, 2022b	1487	Smoker	Not-smoker	aHR	0.9 (0.57-1.42)
Nakafero, 2022b	1487	Alcohol low 1-14 units/week	Alcohol none	aHR	0.96 (0.63-1.46)
Nakafero, 2022b	1487	Alcohol medium 15-21 units/week		aHR	0.86 (0.26-2.86)
Nakafero, 2022b	1487	Alcohol hazardous >21 units/week		aHR	1.12 (0.47-2.69)
Nakafero, 2022b	1487	Alcohol ex-use		aHR	0.84 (0.37-1.87)
Nakafero, 2022b	1487	PMR or GCA	Rheumatoid arthritis	aHR	1.03 (0.46-2.3)
Nakafero, 2022b	1487	Ankylosing spondylitis	Rheumatoid arthritis	aHR	1.14 (0.76-1.7)
Nakafero, 2022b	1487	Epilepsy		aHR	4.39 (1.74-11.06)
Nakafero, 2022b	1487	Diabetes		aHR	0.88 (0.48-1.6)
Nakafero, 2022b	1487	Chronic Kidney Disease		aHR	1.72 (0.96-3.06)
Nakafero, 2022b	1487	Methotrexate or 5-aminosalicylate		aHR	0.93 (0.64-1.35)
Nakafero, 2022b	1487	Statins		aHR	1.44 (0.94-2.22)
Nakafero, 2022b	1487	Paracetemol		aHR	1.45 (0.98-2.16)
Nakafero, 2022b	1487	Blood-test abnormalities in first 6 months of primary-care prescription		aHR	3.06 (2.15-4.35)

[1]RM: Risk measure;

Table S18b: Prognostic factors for either cytopenia, acute kidney injury, or elevated liver enzymes toxicity in people with autoimmune rheumatic diseases prescribed Leflunomide

	Summary of findings	Quality of evidence	Reason for grading_up or down
Age	No evidence of increased risk with age_from one study (Nakafero, 2022b) with 1487 participants.	Very low	Single study
Sex	No evidence of increased risk for female over male sex from one study (Nakafero, 2022b) with 1487 participants.	Very low	Single study
BMI	No evidence of increased risk with BMI from one study (Nakafero, 2022b) with 1487 participants.	Very low	Single study
Smoking status	No evidence of increased risk with smoking_from one study (Nakafero, 2022b) with 1487 participants.	Very low	Single study
Alcohol intake	No evidence of increased risk with alcohol intake_from one study (Nakafero, 2022b) with 1487 participants.	Very low	Single study
Disease	No evidence of increased risk with disease type_from one study (Nakafero, 2022b) with 1487 participants.	Very low	Single study
Epilepsy	Evidence of increased risk with epilepsy	Moderate	Downgrade one single study; upgrade 2 - large effect size

Diabetes, CKD-3	No evidence of increased risk with comorbid CKD-3 or diabetes from one study (Nakafero, 2022b) with 1487 participants.	Very low	Single study, inconsistency
Other immuno-suppressants	No of increased risk with co-prescription of immune supressing drugs from one study (Nakafero, 2022b) with 1487 participants.	Very low	Single study
Other drugs	No evidence of increased risk with statins or paracetamol from one study (Nakafero, 2022b) with 1487 participants,	Very low	Single study
Abnormal blood test results	Evidence of increased risk with prior blood abnormalities within first 6 months of treatment from one study (Nakafero, 2022b) with 1487 participants.	Moderate	Downgrade one - single study; upgrade two – large effect size

Table S19a: Prognostic factors for either cytopenia, acute kidney injury, or elevated liver enzymes in people with autoimmune rheumatic diseases prescribed Mycophenolate mofetil

Author, year	N	Exposure	Referent	RM[1]	Point estimate (95%CI)
Nakafero, 2022a	992	Age at prescription		aHR	1.01 (0.99-1.02)
Nakafero, 2022a	992	Female sex	Male sex	aHR	0.75 (0.5-1.12)
Nakafero, 2022a	992	BMI <18.5 kg/m^2	BMI 18.5-24.9	aHR	0.89 (0.33-2.43)
Nakafero, 2022a	992	BMI 25-29.9 kg/m^2	BMI 18.5-24.9	aHR	0.9 (0.55-1.49)
Nakafero, 2022a	992	BMI >30 kg/m^2	BMI 18.5-24.9	aHR	1.18 (0.73-1.92)
Nakafero, 2022a	992	Smoker	Not-smoker	aHR	0.71 (0.39-1.3)
Nakafero, 2022a	992	Alcohol low 1-14 units/week	Alcohol none	aHR	0.86 (0.54-1.37)
Nakafero, 2022a	992	Alcohol medium 15-21 units/week		aHR	0.57 (0.2-1.64)
Nakafero, 2022a	992	Alcohol hazardous >21 units/week		aHR	0.85 (0.43-1.68)
Nakafero, 2022a	992	Alcohol ex-use		aHR	0.53 (0.24-1.14)
Nakafero, 2022a	992	SLE	No SLE	aHR	1.28 (0.85-1.93)
Nakafero, 2022a	992	Amino-salicylates	No other drugs	aHR	0.17 (0.02-1.27)
Nakafero, 2022a	992	MTX, LEF, AZA or 6-MP		aHR	1.06 (0.26-4.42)
Nakafero, 2022a	992	Chronic kidney disease	No chronic kidney disease	aHR	2.22 (1.47-3.37)

[1]RM: Risk measure; [2]SLE: systemic lupus erythematosus; [3]MTX: methotrexate; [4]LEF:leflunomide; [5]AZA:azathioprine; [6]6-MP:6-mercaptopurine;

Table 19b: Prognostic factors for either cytopenia, acute kidney injury, or elevated liver enzymes in people with autoimmune rheumatic diseases prescribed Mycophenolate mofetil

	Summary of findings	Quality of evidence	Reason for grading up or down
Age	No evidence for association between age and toxicity from one study (Nakafero, 2022) 992 participants.	Very low	Downgrade one for single study
Sex	No evidence for association between age and toxicity from one study (Nakafero, 2022) 992 participants.	Very low	Downgrade one for single study.
Body mass index (BMI)	No evidence for association between BMI and toxicity from one study (Nakafero, 2022) 992 participants.	Very low	Downgrade one for single study
Lifestyle	No evidence for association between lifestyle factors and toxicity from one study (Nakafero, 2022) 992 participants.	Very low	Downgrade one for single study
Disease	No evidence for association between SLE and toxicity from one study (Nakafero, 2022) 992 participants.	Very low	Downgrade one for single study
Other immuno-suppressants	No evidence for association with concomitant therapy from one study (Nakafero, 2022) 992 participants.	Very low	Downgrade one for single study
Chronic kidney disease (CKD)	Evidence for association between CKD stage-3 and toxicity from one study (Nakafero, 2022) 992 participants.	Moderate	Downgrade one for single study, upgrade two for large effect size

Table S20a: Prognostic factors for hepatotoxicity (alanine transaminase increase) in inflammatory bowel disease patients prescribed different drugs*.

Prognostic factor Author, year	N	Exposure	Referent	RM[1]	Point estimate (95% CI)
Age					
Koller, 2017	251	Age per year increase		OR	1.01 (0.98-1.04)
Koller, 2017	251	Age per year increase		OR	1.02 (0.98-1.05)**
Sex					
Koller, 2017	251	Female	Male	aOR	0.22 (0.07-0.67)
Koller, 2017	251	Female	Male	OR	0.55 (0.19-1.55)**
Body mass index (BMI)					
Koller, 2017	251	BMI per kg/m^2 increase		aOR	1.15 (1.05-1.27)
Koller, 2017	251	BMI per kg/m^2 increase		aOR	1.13 (1.02-1.26)**
Liver					
Koller, 2017	251	Liver steatosis	None	aOR	31.0 (6.76-142.10)
Koller, 2017	251	Liver steatosis	None	aOR	10.61 (2.22-50.70)**
Inflammatory bowel disease (IBD) type, activity					
Koller, 2017	251	CD	UC	OR	2.03 (0.83-4.97)
Koller, 2017	251	CD	UC	OR	2.89 (0.80-10.41)**
Koller, 2017	251	C-reactive protein		OR	0.98 (0.94-1.03)
Koller, 2017	251	C-reactive protein		OR	0.98 (0.93-1.04)**
Koller, 2017	251	IBDQ[5] score		OR	1.00 (0.97-1.04)
Koller, 2017	251	IBDQ[5] score		OR	0.99 (0.95-1.04)**
Koller, 2017	251	Fecal calprotectin		OR	1.00 (0.99-1.00)
Koller, 2017	251	Fecal calprotectin		OR	1.00 (0.99-1.00)**
Koller, 2017	251	Intestinal surgery	None	OR	0.95 (0.40-2.28)
Koller, 2017	251	IBD duration		aOR	1.07 (1.00-1.15)**

[1]RM: Risk measure; [2]5-ASA: 5-acetyl salicylic acid;[3]CD: Crohn's disease; [4]UC: Ulcerative Colitis;[5]IBDQ: IBD questionnaire *Patients in this cohort were prescribed 5-ASA, antibiotics, corticosteroids, azathioprine, anti-TNF-alpha, or no immunosuppressive treatments; **outcome was ALT >2-fold upper limit of normal

Table S20b. Summary of GRADE judgements: Prognostic factors for hepatotoxicity (alanine transaminase increase) in inflammatory bowel disease patients prescribed different drugs

	Summary of findings	Quality of evidence	Reason for grading up or down
Age	No evidence for association between age and hepatotoxicity from one study (Koller, 2017) 251 participants.	Very low	Downgrade one for single study
Sex	Evidence of negative association between female sex and hepatotoxicity from one study (Koller, 2017) 251 participants.	Very low	Downgrade one for single study, upgrade one for large effect size, downgrade one for inconsistency.
Liver steatosis	Evidence of association between liver steatosis and hepatotoxicity from one study (Koller, 2017) 251 participants.	Moderate	Downgrade one for single study. Upgrade two for large effect size.
IBD type	No evidence for association between IBD type and hepatotoxicity from one study (Koller, 2017) 251 participants	Very low	Downgrade one for single study
Disease activity	No evidence for association between disease activity and hepatotoxicity from one study (Koller, 2017) 251 participants	Very low	Downgrade one for single study

Table S21a: Prognostic factors for neutropenia in Rheumatoid Arthritis (RA) patients prescribed any disease modifying anti-rheumatic drugs including biologics

Prognostic factor Author, year	N	Exposure	Referent	RM[1]	Point estimate (95% CI)
Laboratory abnormalities (haematologic)					
Fragoulis, 2018	771	Baseline neutrophil count		β	-0.491
Fragoulis, 2018*	771	Baseline Anaemia		OR	1.81 (1.01-3.23)
Fragoulis, 2018*	771	Baseline Thrombocytopenia		OR	2.48 (0.05-22.71)
Fragoulis, 2018*	771	Baseline Leucopaenia		OR	72.35 (33.98-155.46)
Fragoulis, 2018*	771	Baseline Lymphopenia		OR	1.81 (0.86-3.57)

[1]RM: Risk measure; *reviewer calculated from crude data; OR odds ratio

Table S21b. Summary of GRADE judgements: Prognostic factors for neutropenia in RA patients prescribed any DMARDs including biologics

Prognostic factor	Summary of findings	Quality of evidence	Reason for grading up or down
Cytopenia	Evidence from one study (Fragoulis, 2018) with 771 participants, that baseline leucopenia, and low neutrophil count were associated with neutropenia.	Moderate	Single study - downgrade one; large effect size – upgrade two
Anaemia	Evidence from one study (Fragoulis, 2018) with 771 participants, that baseline anaemia is associated with neutropenia.	Very low	Single study

Table S22. Summary of prognostic factors for liver, blood and kidney adverse events in patients taking immune-supressing or biologic drugs

**Hepatotoxicity**			
	**Anti-TNF**	**Methotrexate**	**Thiopurines**
**Increased risk**	Liver disease, elevated liver enzymes +++<br>Body mass index +<br>Comorbidities ++	Elevated liver enzymes +++<br>Excess alcohol ++<br>Diabetes ++<br>Psoriasis vs. RA ++<br>Pre-existing liver disease++<br>Comorbidity score +<br>Poor metaboliser +<br>Leflunomide++	Elevated liver enzymes ++<br>Mercaptopurine (vs. azathioprine)+++<br>Age ++<br>Male sex ++
**No association**	RF/ANA ++<br>Other drugs +	Age +<br>Smoking +<br>RF/ACPA +<br>Disease severity/activity +<br>Inflammatory markers +<br>Disease duration +<br>Anti-TNF +<br>Methotrexate dose +<br>Inflammatory markers +	Smoking ++<br>Disease activity +<br>Immune suppressing drugs +<br>Azathioprine +
**Reduced risk**		Folate supplementation +++	
**Cytopenia/neutropenia**			
	**Anti-TNF**	**Methotrexate**	**Thiopurines**
**Increased risk**	Previous neutropenia +	Chronic liver disease +	Mercaptopurine (vs. azathioprine) +++<br>Poor metaboliser +++
**No association**	Age+<br>Sex+<br>Disease type +		Smoking +<br>Disease activity +
**Reduced risk**	Baseline neutrophil count +		
**Inconsistent**			Female Sex +
**Nephrotoxicity**			
	**Anti-TNF**	**Methotrexate**	**Thiopurines**
**Increased risk**	Risk factor for renal function decline+	NSAIDs +	
**Cytopenia, AKI, elevated liver enzymes**			
	**Leflunomide**		
**Increased risk**	Epilepsy ++<br>Blood abnormalities in first six months of treatment ++		
**Cytopenia, acute liver injury, elevated liver enzymes**			
	**Mycophenolate mofetil**		
**Increased risk**	Chronic kidney disease stage-3 ++		

+++ high quality evidence; ++ moderate quality evidence; +low quality evidence as per GRADE
